# BMI distribution and sex- and grade-specific trends in primary school health surveillance records in Xi’an, China, 2014–2024: a repeated cross-sectional study

**DOI:** 10.3389/fpubh.2026.1942237

**Published:** 2026-10-28

**Authors:** Chengwei Xu, Han Hao, Bing Shi

**Affiliations:** 1 Faculty of Teacher Development, Shaanxi Normal University, Xi’an, China; 2 School of Physical Education, Xi’an Fanyi University, Xi’an, China; 3 School of Physical Education, Shaanxi Normal University, Xi’an, China

**Keywords:** BMI distribution, childhood obesity, China, high BMI, primary-school students, quantile regression, school health surveillance, sex-by-grade heterogeneity

## Abstract

**Background:**

Conventional childhood obesity surveillance relies mainly on mean body mass index (BMI) and overweight/obesity prevalence, which may mask unequal changes across the BMI distribution and population subgroups. Whether long-term BMI increases are concentrated in the upper tail and how high-BMI prevalence trends differ jointly by sex and school grade remain insufficiently characterized in consecutive school surveillance data.

**Methods:**

We analyzed 7,187,779 records from students aged 6.0–12.99 years across 1,394 school codes and 13 districts in Xi’an, China, during 2014–2024 using a repeated cross-sectional design. High BMI comprised overweight or obesity under national sex- and grade-specific cutoffs. Primary analyses used logistic regression to estimate high-BMI prevalence and quantile regression to estimate covariate-adjusted changes in conditional BMI quantiles at the 50th, 75th, 90th, and 95th percentiles (P50, P75, P90, P95). Models adjusted for age in months, sex, grade, and district, with school-clustered inference. Secondary analyses assessed sex-by-grade differences in prevalence change; exploratory analyses examined P5, P10, and P25.

**Results:**

Between 2014 and 2024, observed high-BMI prevalence increased from 17.01 to 21.11%. Adjusted prevalence peaked at 22.75% in 2020; its 2014–2024 increase was 3.35 percentage points (95% confidence interval [CI] 2.69–4.01). Covariate-adjusted changes in conditional BMI quantiles were 0.134 kg/m2 at P50 and 0.735 kg/m2 at P95; the P95 − P50 difference in 2014–2024 change was 0.601 kg/m2 (95% CI 0.468–0.734; *p* < 0.001). Exploratory estimates were 0.097 kg/m2 at P5 (pointwise 95% CI 0.027–0.167), 0.047 at P10 (−0.008–0.103), and 0.048 at P25 (−0.002–0.097). The increase in adjusted high-BMI prevalence was 1.65 percentage points greater among boys than girls (95% CI 1.17–2.14); the difference within Grades 4–6 was 2.59 percentage points (95% CI 1.95–3.23).

**Conclusion:**

Among participating-school surveillance records, adjusted high-BMI prevalence increased between 2014 and 2024, with greater increases among boys than girls overall and within Grades 4–6. BMI increases were greater at the upper conditional quantiles than at the median. Joint reporting of high-BMI prevalence, upper-tail BMI changes, and sex- and grade-specific estimates may provide a more complete picture of BMI trends.

## 1 Introduction

Worldwide surveillance shows that childhood overweight and obesity have increased over recent decades (1). These conditions are important public-health concerns because they are associated with adverse health outcomes (2), can persist into adulthood (3), and have been linked to morbidity and premature mortality later in life (4). Schools are an important setting for childhood-obesity prevention and health promotion among school-aged children (5). In this study, body mass index (BMI) is used for screening and surveillance. The term ‘high-BMI burden’ refers to the prevalence of overweight or obesity under the national sex- and grade-specific cut-points; it does not directly measure adiposity, body composition, metabolic risk, or individual health or disease burden.

Routine childhood-obesity surveillance commonly reports mean BMI and the prevalence of overweight and obesity. These summaries may conceal unequal changes across the BMI distribution and population subgroups, limiting the information available to identify groups for further assessment. Reporting changes at lower quantiles, the median, and upper quantiles can provide a fuller description of BMI trends. In covariate-adjusted analyses, these conditional quantiles describe distributional positions rather than clinical weight-status categories.

Chinese evidence makes this distinction empirically relevant. National analyses covering 1985–2010 and 1997–2011 documented rightward BMI shifts, wider distributions, and larger increases at higher percentiles (6, 7). A two-wave study of urban children in Changsha similarly found larger changes at P85 and P95 than at P5 and P50 between 2002 and 2018 (8).

Nutrition-transition research provides a broader context for the coexistence of undernutrition and excess weight (9–11). In China, national school surveys document changes in nutritional status and urban–rural differences, while recent estimates and projections indicate a continuing need for surveillance of childhood overweight and obesity (12–17).

Previous Chinese studies have reported sex- and age-related differences in obesity prevalence and trends. National surveys from 1985 to 2010 found a stronger obesity trend among boys than girls (18), whereas a 2011–2015 study found a significant increase in obesity prevalence among adolescents aged 12–18 years but not younger children (19). These findings do not establish how annual changes in high-BMI prevalence vary jointly by sex and primary-school grade. We therefore examined sex-by-grade heterogeneity in changes in high-BMI prevalence, using grade as the school-stage indicator and age in months to account for within-grade age variation.

The Xi’an surveillance system provides 11 consecutive annual waves of participating primary-school records across 13 districts. These records support comparisons over time within a common municipal reporting system. Stable school codes also permit school-clustered inference and fixed-school sensitivity analyses. District and school analyses can describe variation that overall surveillance summaries may conceal; school resource information was available for 2024. The findings apply to participating-school records; municipal and national population representativeness have not been established.

What remains insufficiently characterized in consecutive annual school surveillance is whether BMI increases are concentrated at the upper conditional quantiles and how changes in high-BMI prevalence vary jointly by sex and school grade. Examining these questions together may provide a more informative account of childhood BMI trends than either prevalence or distributional summaries alone.

The primary objectives were to estimate adjusted annual high-BMI prevalence and its 2014–2024 change, estimate covariate-adjusted changes in conditional BMI quantiles at P50, P75, P90, and P95, and test whether upper-quantile changes exceeded the median change. Secondary analyses assessed sex- and grade-specific heterogeneity in changes in high-BMI prevalence and district variation, while exploratory analyses examined lower conditional BMI quantiles, school burden patterns, and associations with physical-education (PE) resources in 2024. WHO classification was evaluated in sensitivity analyses.

## 2 Materials and methods

### 2.1 Study design and data sources

We conducted a repeated cross-sectional study using annual primary-school records submitted through the National Student Physical Health Testing system from 13 districts in Xi’an between 2014 and 2024. Previous national research showed that coverage of school-health monitoring components varied by geography and school type (20). We followed the Strengthening the Reporting of Observational Studies in Epidemiology (STROBE) and Reporting of Studies Conducted Using Observational Routinely Collected Health Data (RECORD) guidelines (21, 22). Under the national framework, schools are required to organize annual physical-health testing of enrolled students and submit the results to the national data system after review by education authorities (23–25). Students entered the database through this routine school-organized testing and reporting process; the investigators did not recruit or sample students for the present study. However, the supplied extract did not include an independently audited roster of all eligible schools and students or documentation of compliance with the national reporting requirement. We therefore could not verify complete participation or reporting or calculate annual school- or student-level coverage and changes in coverage during 2014–2024. Each record contained year, district, stable school code, sex, grade, date of birth, height, weight, BMI, and weight-status category. School-code counts and testing records were obtained from the annual reporting system of the National Student Physical Health Standards Data Management Center. The national framework also links physical-health testing, reporting, pass rates, excellence rates, and health trends to school monitoring and evaluation (24, 25).

We included records from Grades 1–6. High BMI was defined as overweight or obesity according to sex- and grade-specific thresholds in the National Student Physical Health Standard (2014 Revision) (23). Grade was selected as the school-stage stratum because surveillance reporting and the national BMI classification are organized by grade. Age was retained as a separate covariate to account for age variation within grades. Because exact individual measurement dates were unavailable, we defined standardized-reference age as age on September 1 of each survey year. September 1 was used as a common school-year reference to apply the same age definition across all records, years, and grades; it was not treated as the individual measurement date. Field testing occurred annually between October 15 and November 15, so the reference date preceded measurement by 44–75 days. We retained this definition to preserve a consistent age scale while recognizing that it underestimated age at measurement. Eligibility was restricted to ages 6.0–12.99 years on the basis of standardized-reference age. For modeling, standardized-reference age in days was converted to monthly intervals using 365.25 days per year and represented by the corresponding interval midpoint. Restricted cubic spline knots were derived from the same distribution of interval midpoints. The basis functions were centered and whitened over a fixed grid of 72–155 months.

### 2.2 Participants, sample construction, and quality control

The source data comprised 143 district-year files and 7,278,755 records. This secondary analysis used all available surveillance records meeting the eligibility criteria; no *a priori* sample-size calculation was performed. We excluded 2,731 non-primary-grade records. Among the remaining 7,276,024 primary-grade records, 265 had missing or invalid height or weight and 4,155 additional records had another major anthropometric quality-control (QC) flag, leaving 7,271,604 QC-valid records before the administrative exclusion. Four school codes each showed a single change in the recorded district between 2019 and 2020. To avoid uncertain cross-year district attribution, we excluded all 71,532 QC-valid records associated with these codes across 2014–2024. This school-code-level exclusion was separate from anthropometric QC; the records had passed those checks. The available study records did not establish the cause of the district-field changes. We evaluated the effect of this exclusion by repeating the primary models with these schools included (Supplementary material SM7; Supplementary Table SM6). The final cleaned sample contained 7,200,072 records and 1,394 school codes. Annual exclusions for missing/invalid anthropometry or an anthropometric QC flag ranged from 0.043 to 0.105% of primary-grade records; across the 143 district-year files, the corresponding proportions ranged from 0.006 to 0.547%. The primary and secondary record-level analyses used records complete for the variables required by their respective models after the exclusions described above; no model-based or multiple imputation was performed. The 992 source weight-status entries that were missing or unmapped did not create a missing outcome because weight status was rederived from height, weight, sex, and grade. For the primary analysis, we additionally excluded one record with an unparseable date of birth and 12,292 records outside the standardized-reference age range. The primary analysis sample therefore comprised 7,187,779 complete records from students aged 6.0–12.99 years. The complete selection process is shown in Figure 1 and Supplementary Tables S1, S2.

**Figure 1:**
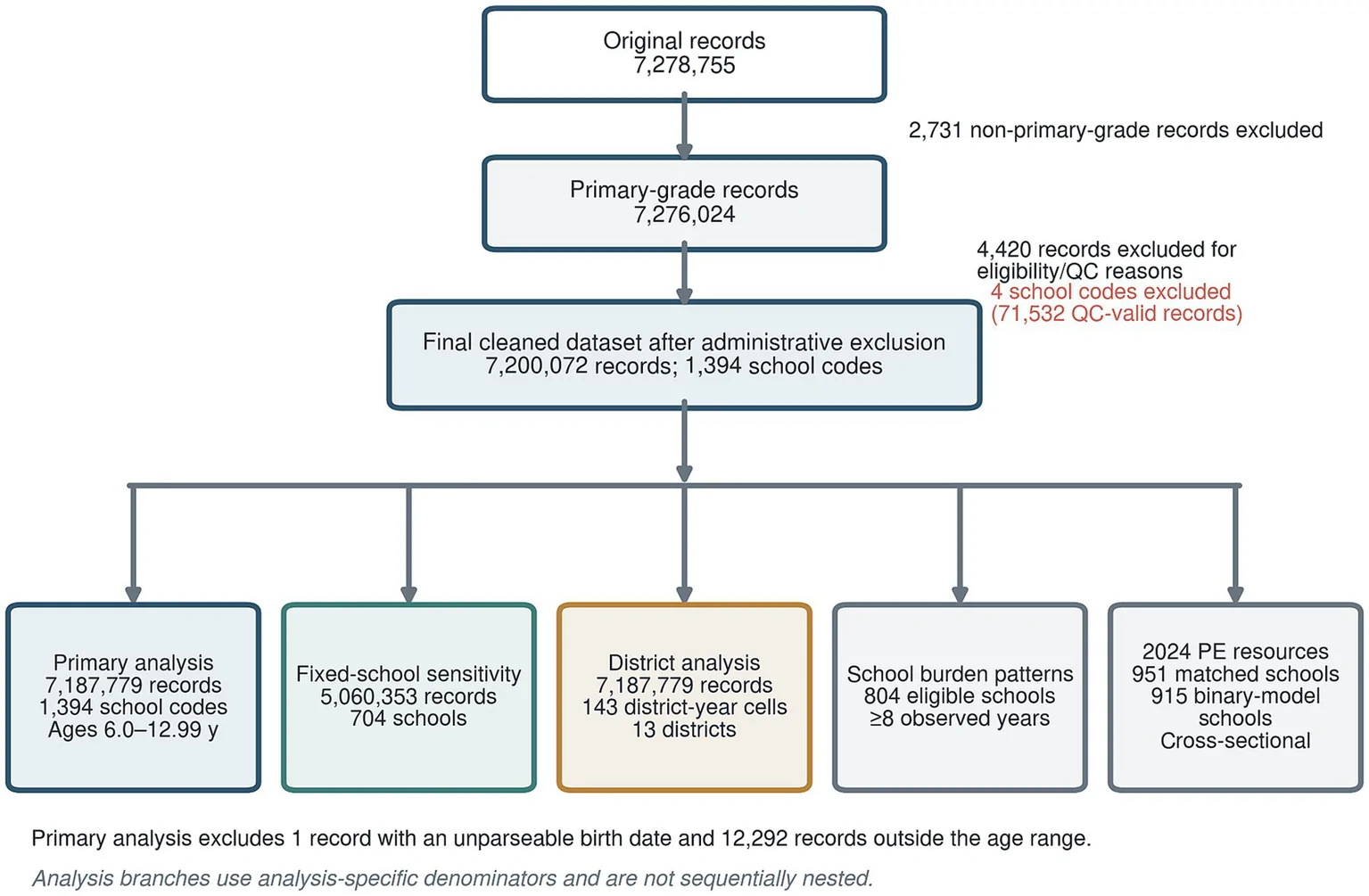
Study flow, analytic denominators, and analysis-specific sample construction.

The primary analysis sample was used for adjusted high-BMI models, conditional quantile regression, and subgroup interaction analyses. The fixed-school panel comprised 704 schools observed in all 11 years. The school burden-pattern analysis included 804 classifiable schools. PE-resource descriptions and weighted linear models included 951 matched schools; binary high-burden models included 915 because 36 had fewer than 50 records in 2024. This reduction reflected the outcome-classification threshold rather than missing model variables. Figure 1 summarizes the analysis-specific samples.

The flowchart begins with 7,278,755 records and shows the exclusion of 2,731 non-primary-grade records, 4,420 records for eligibility or quality-control reasons, and 71,532 QC-valid records from four school codes whose recorded district changed between 2019 and 2020. These exclusions yielded 7,200,072 final cleaned records and 1,394 school codes. The primary age restriction excluded one record with an unparseable birth date and 12,292 records outside 6.0–12.99 years. Analysis branches use distinct denominators and are not sequentially nested: primary analysis, 7,187,779 records; age-restricted fixed-school sensitivity, 5,060,353 records in 704 schools; district analysis, 7,187,779 records forming 143 district-year cells across 13 districts; school-level adjusted high-BMI burden patterns, 804 eligible schools; and PE-resource matching and weighted linear analysis, 951 schools, with 915 schools in binary high-burden logistic models. Navy indicates the primary analysis, green the fixed-school sensitivity, ochre the secondary district analysis, and gray the exploratory analyses. QC, quality control; PE, physical education.

Quality-control procedures included verification of the year and district recorded in each file, field standardization, investigator recalculation of BMI, reclassification of weight status, and screening for implausible values. The research team calculated the BMI outcome used in all analyses directly from recorded height and weight. Before anthropometric exclusions, stored BMI equaled weight (kg)/height (m)^2^ for all 7,275,759 recalculable primary-grade records. The reporting system applied student-registration and testing-record rules to check for multiple records from the same student within a year. We also conducted an exact-match record-signature audit using key fields. Because unique longitudinal student identifiers were unavailable, this audit identified record collisions rather than individual-level duplication. Supplementary Table S2 reports the raw-file structure before exclusion of the four schools with district-field changes, the BMI recalculation audit, and the audit of year-to-year changes within schools. Analyses of recording precision and terminal-digit stability are described in Section 2.3 and Supplementary Tables S3, S11.

### 2.3 Anthropometric measurements and outcome definitions

Anthropometric measurements were organized each year through the National Student Physical Health Testing reporting system and followed the National Student Physical Health Standard (2014 Revision) (23). Members of the research team participated in examiner training, field organization, physical fitness testing, and data compilation as part of the annual routine surveillance program. These activities were undertaken to implement the established surveillance program rather than specifically for the present research. The present study used authorized de-identified records after the annual surveillance records had been generated and did not involve additional recruitment, intervention, measurement, or participant contact for research purposes. Field testing was conducted annually within a standardized window from October 15 to November 15. Examiners were physical education or health professionals; newly appointed examiners received standardized training and were required to pass an assessment. Students were measured barefoot and wore shorts and a short-sleeved top or other close-fitting light clothing. Height was measured with a mechanical stadiometer or a combined mechanical standing- and sitting-height meter. Before testing, the upright column, connections, and zero point were checked, and the scale was verified with a standard steel tape measure. The smallest scale division was no greater than 0.1 cm, measurement error over 100 cm was no greater than 0.1 cm, and height was recorded to the nearest 0.1 cm. Weight was measured with an electronic scale. Before testing, sensitivity was checked with a 100-g standard weight, and accuracy was checked with 10-, 20-, and 30-kg standard weights. Error did not exceed 0.1%, and weight was recorded to the nearest 0.1 kg. We recalculated BMI as weight (kg)/height (m)^2^. For national-standard classification, recalculated BMI was rounded to one decimal using numpy.round(BMI, 1) in NumPy 2.3.5, and the published one-decimal cut-points were applied. The complete sex- and grade-specific underweight, normal-weight, overweight, and obesity ranges are reported in Table 1; high BMI comprised the overweight and obesity categories.

**Table 1:** Sex- and grade-specific BMI classification cut-points from the National Student Physical Health Standard (2014 revision).

Sex	Grade	Underweight	Normal weight	Overweight	Obesity
Boys	1	≤13.4	13.5–18.1	18.2–20.3	≥20.4
Boys	2	≤13.6	13.7–18.4	18.5–20.4	≥20.5
Boys	3	≤13.8	13.9–19.4	19.5–22.1	≥22.2
Boys	4	≤14.1	14.2–20.1	20.2–22.6	≥22.7
Boys	5	≤14.3	14.4–21.4	21.5–24.1	≥24.2
Boys	6	≤14.6	14.7–21.8	21.9–24.5	≥24.6
Girls	1	≤13.2	13.3–17.3	17.4–19.2	≥19.3
Girls	2	≤13.4	13.5–17.8	17.9–20.2	≥20.3
Girls	3	≤13.5	13.6–18.6	18.7–21.1	≥21.2
Girls	4	≤13.6	13.7–19.4	19.5–22.0	≥22.1
Girls	5	≤13.7	13.8–20.5	20.6–22.9	≥23.0
Girls	6	≤14.1	14.2–20.8	20.9–23.6	≥23.7

BMI is expressed in kg/m^2^. Categories reproduce the sex- and grade-specific one-decimal cut-points in the National Student Physical Health Standard (2014 Revision) (23). In the analysis, BMI was recalculated as weight (kg)/height (m)^2^ and classified after numpy.round(BMI, 1) in NumPy 2.3.5. High BMI was defined as overweight or obesity.

We used the national standard in the primary analysis to retain the sex- and grade-specific classification used by the school surveillance system. To assess sensitivity to classification and aid international interpretation, we added the WHO 2007 BMI-for-age reference (26). WHO-defined high BMI comprised overweight including obesity (BMI-for-age Z score > + 1); obesity was defined as Z score > + 2. WHO classification used unrounded recalculated BMI and sex- and age-specific reference values.

To assess whether recording precision affected inference, we rounded height and weight in two sensitivity analyses: first to the nearest 0.5 cm and 0.5 kg and then to the nearest 1 cm and 1 kg. We recalculated BMI and weight status and repeated the primary high-BMI and P50/P95 models. We also constructed fixed-school subsets using empirical P90 and P95 thresholds for changes in the proportion of integer terminal digits between adjacent school-years. These analyses assessed sensitivity to recording precision and year-to-year changes within schools; the original measurements were not altered.

Terminal-digit stability screens were empirical sensitivity analyses, not primary-sample exclusion criteria or externally validated quality-control standards. We applied P90 and P95 limits for adjacent-year changes in integer-valued height and weight reporting within the fixed-school panel; the algorithm, thresholds, sample sizes, and absence of preregistration are described in Supplementary material SM1 and Supplementary Table S3.

### 2.4 Record-level statistical analyses

For each year, we summarized record and school-code counts, sex and grade composition, age, height, BMI, and weight status in the final cleaned sample. Age, height, and BMI were reported as means and standard deviations (SDs); weight status included underweight, normal weight, overweight, and obesity. Age was summarized among records with a calculable September 1 age. Annual high-BMI prevalence was calculated as the number of records classified as overweight or obese divided by the number of classifiable records. The summaries presented in Table 2 are unadjusted descriptive values and may partly reflect changes in the age and grade composition of participating students across years. Prevalence and conditional quantiles summarize different aspects of BMI and were reported as complementary measures. P50 represented the distributional center, P90 the upper decile, and P95 the more extreme upper tail. We reported observed BMI at P50, P75, P90, and P95 and the observed P95 − P50 difference.

**Table 2:** Annual sample composition and observed anthropometric and nutritional status.

Panel A. Sample composition											
Year	Records	School codes	Boys, %	Girls, %	Age, mean (SD), y	Grade 1, %	Grade 2, %	Grade 3, %	Grade 4, %	Grade 5, %	Grade 6, %
2014	470,204	1,092	53.97	46.03	8.80 (1.75)	18.72	17.97	16.68	16.18	15.84	14.61
2015	503,275	1,176	53.59	46.41	8.84 (1.75)	18.08	18.01	17.28	15.98	15.54	15.12
2016	510,439	1,137	53.28	46.72	8.82 (1.73)	19.12	17.60	17.24	16.40	15.05	14.58
2017	555,416	1,111	53.11	46.89	8.82 (1.72)	19.21	18.40	16.56	16.19	15.40	14.23
2018	612,173	1,023	52.83	47.17	8.79 (1.74)	20.50	18.21	17.04	15.27	14.91	14.07
2019	661,865	1,013	52.61	47.39	8.79 (1.74)	19.70	19.30	17.04	15.92	14.21	13.82
2020	712,573	1,011	52.46	47.54	8.80 (1.75)	19.65	18.46	18.03	15.86	14.80	13.20
2021	749,051	997	52.36	47.64	8.90 (1.72)	17.24	18.79	17.62	17.17	15.13	14.07
2022	777,826	998	52.28	47.72	8.95 (1.75)	17.19	16.80	18.06	16.95	16.49	14.50
2023	820,252	983	52.13	47.87	8.95 (1.78)	18.63	16.65	15.95	17.12	16.05	15.60
2024	826,998	963	52.06	47.94	9.01 (1.76)	16.06	18.54	16.54	15.80	17.11	15.96
Overall	7,200,072	1,394	52.68	47.32	8.87 (1.75)	18.44	18.04	17.10	16.30	15.56	14.56

Panel B. Anthropometric and nutritional status.							
Year	Height, mean (SD), cm	BMI, mean (SD), kg/m^2^	Underweight, %	Normal weight, %	Overweight, %	Obesity, %	High BMI, %
2014	134.00 (11.55)	17.06 (3.20)	8.06	74.92	10.30	6.73	17.03
2015	135.04 (11.57)	17.22 (3.22)	6.54	75.42	10.74	7.30	18.05
2016	135.33 (11.62)	17.23 (3.24)	6.66	74.76	11.09	7.49	18.57
2017	135.67 (11.75)	17.34 (3.32)	6.27	74.10	11.47	8.15	19.62
2018	135.75 (11.91)	17.35 (3.36)	6.30	73.67	11.55	8.48	20.03
2019	136.17 (11.99)	17.38 (3.43)	6.11	73.70	11.61	8.58	20.19
2020	136.74 (12.06)	17.60 (3.50)	5.61	71.67	12.88	9.83	22.71
2021	137.52 (12.14)	17.66 (3.46)	5.20	72.20	12.81	9.80	22.60
2022	138.16 (12.28)	17.58 (3.48)	5.91	72.36	12.43	9.30	21.73
2023	138.16 (12.50)	17.51 (3.55)	6.57	72.29	11.80	9.35	21.14
2024	138.61 (12.45)	17.47 (3.54)	7.21	71.68	11.77	9.34	21.11
Overall	136.73 (12.13)	17.43 (3.42)	6.35	73.11	11.79	8.75	20.54

Values are unadjusted summaries from the final cleaned sample (7,200,072 records; 1,394 school codes), before the additional age restriction used in the primary analysis. Age was defined on September 1 of each survey year and was available for 7,200,071 records; one 2015 record had an unparseable birth date. Weight-status categories were recalculated using the national sex- and grade-specific cut-points in Table 1; high BMI comprises overweight or obesity. Records are repeated cross-sectional observations, not unique longitudinally followed children. Percentages may not sum exactly because of rounding. BMI, body mass index; SD, standard deviation.

The primary high-BMI analysis used a categorical-year logistic model adjusted for an age-in-months restricted cubic spline, sex, grade, and district. We estimated school-cluster-robust covariance with a finite-cluster correction, grouping all years from the same school code in one cluster (27, 28). Annual prevalence was marginally standardized to the pooled 2014–2024 covariate distribution. Sensitivity analyses used the fixed 2014 distribution, an equal-year standard, and the fixed-school panel standardized to its own pooled distribution. A 9-df school-clustered Wald test assessed departure from a single linear log-odds trend. Standardization and inference details are provided in Supplementary material SM2.

Age in months was modeled with a four-knot restricted cubic spline at 75.5, 95.5, 116.5, and 139.5 months, contributing 3 degrees of freedom. The knots corresponded to the 5th, 35th, 65th, and 95th percentiles of the primary-sample age distribution. Centering and whitening preserved the spline function space and improved numerical conditioning. The full construction is described in Supplementary material SM1.

The primary quantile-regression models estimated covariate-adjusted 2014–2024 changes in conditional BMI quantiles at P50, P75, P90, and P95, using categorical year, the same age spline, sex, grade, and district (29, 30). These estimates describe conditional rather than marginal population quantiles. Custom grouped iteratively reweighted least squares retained record-level BMI outcomes while combining computations for identical covariate patterns. School-level scores and a pooled residual-density approximation were used to estimate clustered covariance and joint cross-quantile contrasts, with 19,999 school-cluster multiplier replicates as an inferential check (31). In a balanced internal sample of 51,480 records, fits at all four quantiles were compared with HiGHS linear-programming solutions. Sensitivity to the pooled-density approximation was assessed for all four quantile changes and the three upper-quantile versus P50 contrasts using observation-level kernel-weighted bread matrices. Complete school-cluster refits were also performed in a separate internal sample. Supplementary material SM3 (Supplementary Tables SM1, SM2) and Supplementary Table S11 report the implementation and validation results. These analytical choices were not preregistered.

Exploratory quantile-regression models at P5, P10, and P25 were added during peer review using the same sample, covariates, and school clustering. We report pointwise 95% confidence intervals for these exploratory estimates. The lower-quantile results and their numerical and covariance checks are reported in Supplementary Table S15 and Supplementary material SM8.

The WHO sensitivity analysis retained the primary sample, model covariates, school clustering, and standardization weights. We evaluated classification age at September 1 and at the testing-window endpoints, 44 and 75 days later, while holding eligibility and model age covariates fixed. An additional analysis excluded WHO extreme-Z-score flags. WHO annual estimates and the original three targeted subgroup contrasts are reported in Supplementary Tables S16, S17; implementation details are in Supplementary material SM9.

The analysis excluding 2020 and 2021 was specified in the analysis workflow before the peer-review revision, but it was not a preregistered primary analysis. Both years were removed together to test whether the endpoint upper-tail contrast depended on observations collected during potentially atypical schooling or testing conditions; the model was otherwise unchanged. This robustness analysis was not used to identify a pandemic-related mechanism.

Secondary subgroup analyses used the same record-level sample and tested year × sex, year × grade, and year × sex × grade interactions. The omnibus interaction tests were the principal tests of subgroup heterogeneity; individual subgroup estimates and targeted endpoint contrasts described the pattern. We report changes in adjusted high-BMI prevalence from 2014 to 2024 and between-group differences in these changes. We used 9,999 school-cluster multiplier bootstrap replicates to assess the robustness of the interval estimates. As a sensitivity analysis of numerical stability, we replaced the age spline with a linear age-in-months term derived from age in days. We then re-estimated the boys−girls contrast, the boys−girls contrast within Grades 4–6, and the targeted Grade 6 − Grade 5 contrast (Supplementary Table S4).

In an additional sensitivity analysis, we included the four excluded school codes using their recorded district in each survey year. We retained the primary age restriction, BMI classification, age-spline knots, model specifications, and school-cluster inference. Standardized prevalence and subgroup contrasts used the original primary-sample reference distribution in both analyses (Supplementary material SM7).

Primary analyses used Python 3.12.13 and custom routines for the logistic, conditional-quantile, school-cluster covariance, and bootstrap procedures. The Supplementary material reports the software versions and computational specifications.

### 2.5 Secondary district analyses

The secondary district analyses used the 7,187,779 primary-analysis records for ages 6.0–12.99 years. District high-BMI prevalence was directly standardized to the sex × grade distribution of the pooled 2014–2024 records in this analytic branch. This estimand differed from that of the primary record-level model, which included an age spline. We described between-district differences using the maximum−minimum difference, P90 − P10 difference, coefficient of variation, difference between the highest and lowest tertiles, and difference between central urban districts and peripheral suburban/county districts. Regional contrasts primarily used means weighted by the number of tested records in each district; equally weighted district means were examined as a sensitivity measure. The 12-cell standard followed the national sex- and grade-specific classification and provided a common composition for district comparisons without fitting a separate district-outcome model. The standardized-reference age restriction limited the sample to 6.0–12.99 years, but no continuous standardization for standardized-reference age in months was applied. Consequently, these district estimates are not age-spline-adjusted equivalents of the primary model, and residual within-grade age composition may remain.

We obtained pointwise 95% confidence intervals for district prevalence and 2014–2024 changes by resampling schools within districts, retaining each school’s observed years together. The pooled sex–grade reference weights were held fixed. Supplementary material SM5 describes the bootstrap procedure and reports endpoint intervals in Supplementary Table SM5.

District analyses were limited to surveillance-derived prevalence and record counts. Contextual-indicator and socioeconomic-gradient analyses were excluded because their source data and linkage could not be verified. Broadband exposure was not analyzed: the available administrative series described Xi’an as a whole rather than comparable annual values for all 13 districts (32, 51). Details of the scope of the retained district analyses are provided in Supplementary material SM5.

### 2.6 Exploratory school-level adjusted high-BMI burden-pattern and PE-resource analyses

School-level high-BMI burden was the observed-minus-expected prevalence in percentage points. The original expected-prevalence model was a grouped binomial model with categorical year, district, sex, and grade, fitted to 7,200,071 records with calculable reference ages. This model standardized for the sex–grade categories used in the national classification but did not adjust for age variation within grades. We summed expected events across each school-year’s sex–grade cells and divided by its record count. High burden was defined relative to the annual P75 of residuals among school-years with at least 50 records. Schools meeting the observation-history criteria were classified as persistent, emerging, declining, fluctuating/intermittent, or stable low burden. These classes describe contemporaneous relative burden. Full rules are provided in Supplementary material SM4 and Supplementary Table S5.

To assess within-grade age sensitivity, we repeated the classification in the primary age range, first without age adjustment and then with the same age-in-months spline as in the primary analysis. Classification rules were retained and annual P75 thresholds recalculated for each model (Supplementary material SM4; Supplementary Tables SM3, SM4).

The 2024 PE-resource report contained 996 unique school codes. After the four-school administrative exclusion, 963 school codes were available in the 2024 BMI analysis, and 951 schools matched by stable school code. Resource indicators were average class size, PE teachers per 1,000 students, partial recorded sports area per student, and a facility-count index. The four final exposures, log school size, district, adjusted burden residual, and record count were complete in the 951-school analysis table; consequently, no school was excluded from the 951-school models because of missing final model variables, and no model-based or multiple imputation was performed. In the upstream 996-school resource report, average class size was undefined for one school (0.10%) because its class count was zero; that school was not in the matched model sample. Football-field count was blank for four schools (0.40%), and the unused current-year new-PE-teacher field was blank for 12 schools (1.20%). Under the archived composite-construction rule, blank or nonnumeric facility and partial-area components were assigned a value of zero before summation. This was deterministic zero coding rather than model-based or multiple imputation; because blank entries could not be independently distinguished from true zero values, some exposure misclassification remains possible. Supplementary Table S6 reports the complete sample flow and model denominators.

The continuous outcome was the adjusted high-BMI burden residual in 2024. We fitted school-level linear models weighted by the number of physical-fitness testing records, with heteroskedasticity-consistent type 3 robust standard errors (33). The binary outcome was P75 high-burden status and was analyzed with logistic models. Both models included all four standardized resource exposures, log school size, and district indicators. Weighted linear models used all 951 matched schools. Binary models included 915 schools with at least 50 records, of which 230 were classified as high burden. The remaining 36 schools did not meet the record-count threshold; outcome, exposure, and covariate values were complete. The four resource exposures and two outcomes yielded eight tested associations. We applied both the Benjamini–Hochberg false discovery rate (BH-FDR) correction and the Holm correction (34, 35). Complete specifications, QC samples, and sensitivities are reported in Supplementary Table S6.

### 2.7 Ethics

The Ethics Committee of Shaanxi Normal University reviewed and approved this retrospective secondary analysis (approval no. SN-NU202516076). Because the study used existing de-identified records, involved no participant contact, and posed minimal risk, the Committee waived the requirements for individual assent and parental or legal guardian informed consent. All analyses were conducted using de-identified information, and public outputs do not disclose school names, original school codes, addresses, or precise geographic coordinates.

## 3 Results

### 3.1 Study population and annual high-BMI patterns

After the exclusions shown in Figure 1, the final cleaned sample comprised 7,200,072 records, of which 7,187,779 met the age criterion for the primary analysis.

The sex- and grade-specific BMI categories used in the following summaries are defined in Table 1.

Annual sample composition and observed anthropometric and nutritional-status summaries in the final cleaned sample are presented in Table 2. The sample included 7,200,072 records from 1,394 school codes; records from boys accounted for 52.68% and records from girls for 47.32%. Annual records increased from 470,204 in 2014 to 826,998 in 2024, while the numbers of retained participating school codes from 2014 through 2024 were 1,092, 1,176, 1,137, 1,111, 1,023, 1,013, 1,011, 997, 998, 983, and 963. Mean standardized-reference age increased from 8.80 (SD 1.75) years in 2014 to 9.01 (SD 1.76) years in 2024. Over the same endpoints, Grade 1 representation decreased from 18.72 to 16.06%, whereas Grade 5 and Grade 6 representation increased from 15.84 to 17.11% and from 14.61 to 15.96%, respectively. One record in 2015 lacked a calculable date-of-birth age but had valid sex, grade, and weight-status data; age summaries therefore included 7,200,071 records. Overall mean height was 136.73 cm (SD 12.13), mean BMI was 17.43 kg/m^2^ (SD 3.42), and 20.54% of records were classified as high BMI. The proportions of height measurements recorded as whole-number values ranged from 86.28 to 91.94%; the corresponding proportions for weight ranged from 71.16 to 84.65%. Results from the conservative precision-reduction sensitivity analysis are reported in Supplementary Table S3 and Supplementary Figure S1.

Table 2 describes the final cleaned sample before the additional date-of-birth and age-range exclusions used in the primary analysis. Between 2014 and 2024, unadjusted mean height increased from 134.00 cm (SD 11.55) to 138.61 cm (SD 12.45), and mean BMI increased from 17.06 kg/m^2^ (SD 3.20) to 17.47 kg/m^2^ (SD 3.54). Observed high-BMI prevalence increased from 17.03 to 21.11%. It rose from 20.19% in 2019 to 22.71% in 2020 and then declined to 21.11% in 2024 (Supplementary Table S7).

Figure 2A shows adjusted annual high-BMI prevalence in the primary analysis sample of 7,187,779 records after the additional date-of-birth and age-range exclusions. In this sample, observed prevalence increased from 17.01% in 2014 to 21.11% in 2024, a change of 4.09 percentage points based on unrounded values. Adjusted prevalence increased from 17.41 to 20.75%, an endpoint change of 3.35 percentage points (95% CI 2.69–4.01; Figure 2B; Table 3, Panel A). It peaked at 22.75% in 2020 (95% CI 22.30–23.21) and declined by 2.00 percentage points by 2024. The categorical-year pattern departed from a single linear log-odds trend (*F*(9,1,393) = 26.14; *p* < 0.001; Supplementary Table S8).

**Figure 2:**
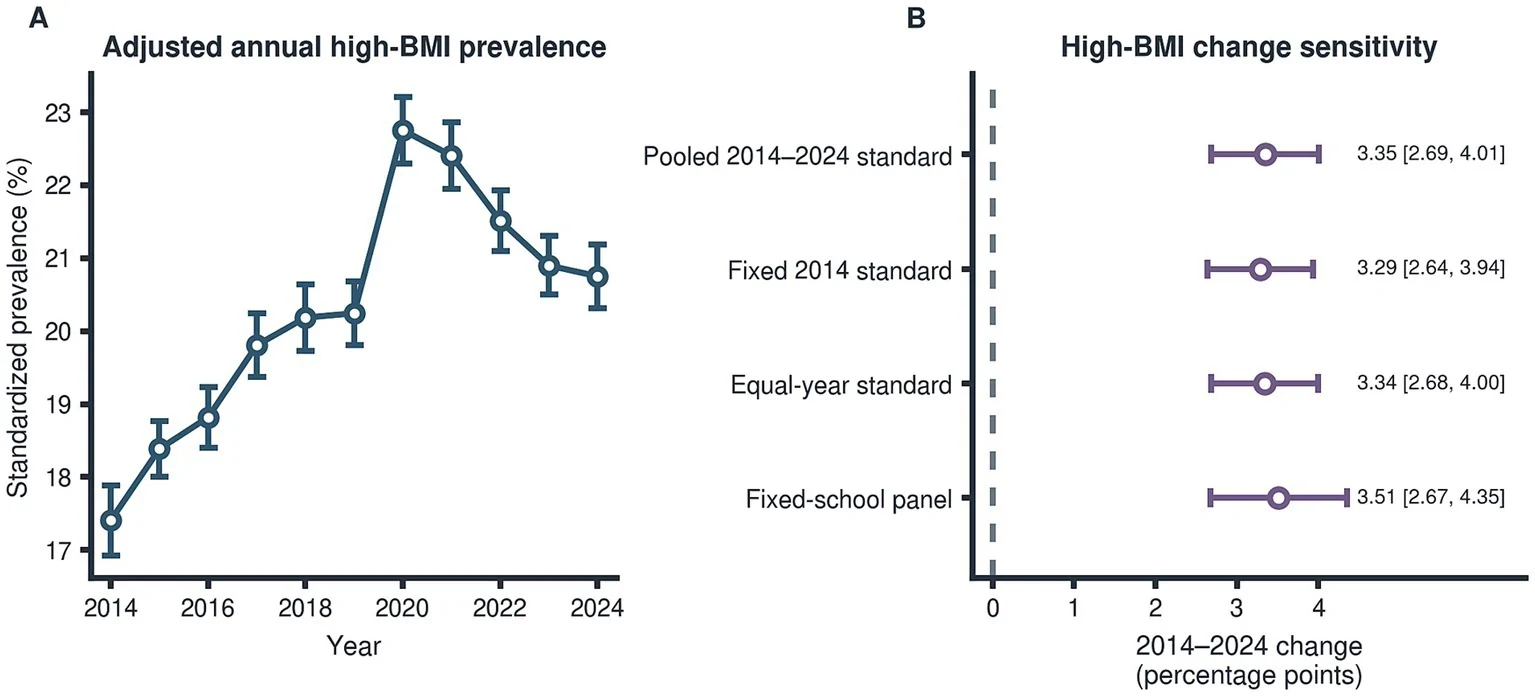
Adjusted annual high-BMI prevalence, assessment of nonlinearity and 2014–2024 sensitivity contrasts. Panel **(A)** shows year-specific high-BMI prevalence in the primary sample (7,187,779 records; 1,394 school codes), standardized to the pooled 2014–2024 age-in-months spline × sex × grade × district distribution. In both panels, error bars show school-cluster-robust 95% confidence intervals. The categorical-year pattern departed from a single linear log-odds trend (*F*(9,1,393) = 26.14; *p* < 0.001). Panel **(B)** compares the 2014–2024 endpoint change under the pooled standard, fixed 2014 standard, equal-year standard and fixed 704-school panel (5,060,353 records). For the fixed-school sensitivity, estimates were standardized to the panel’s own pooled 2014–2024 distribution of the age-in-months spline, sex, grade and district. Numerical annotations in Panel **(B)** report estimates [95% CI]. High BMI denotes overweight or obesity.

**Table 3:** Adjusted primary and subgroup findings, 2014–2024.

Section	Outcome / contrast	Estimate (95% CI)	*p* value	Unit
Panel A. Overall adjusted high-BMI prevalence change				
Overall	Adjusted high-BMI prevalence change, 2014–2024	3.346 (2.685 to 4.007)	<0.001	percentage points
Panel B. Conditional BMI distribution				
BMI distribution	Conditional P50 change	0.134 (0.079 to 0.188)	<0.001	kg/m^2^
BMI distribution	Conditional P75 change	0.345 (0.265 to 0.425)	<0.001	kg/m^2^
BMI distribution	Conditional P90 change	0.620 (0.498 to 0.742)	<0.001	kg/m^2^
BMI distribution	Conditional P95 change	0.735 (0.573 to 0.896)	<0.001	kg/m^2^
BMI distribution	P75 − P50 difference in 2014–2024 change	0.211 (0.171 to 0.252)	<0.001	kg/m^2^
BMI distribution	P90 − P50 difference in 2014–2024 change	0.486 (0.397 to 0.575)	<0.001	kg/m^2^
BMI distribution	P95 − P50 difference in 2014–2024 change	0.601 (0.468 to 0.734)	<0.001	kg/m^2^
Panel C. Subgroup changes and contrasts				
Sex	Boys change	4.109 (3.401 to 4.817)	<0.001	percentage points
Sex	Girls change	2.456 (1.756 to 3.157)	<0.001	percentage points
Sex × grade	Boys, Grade 1 change	−0.714 (−1.958 to 0.530)	0.260	percentage points
Sex × grade	Boys, Grade 2 change	1.533 (0.436 to 2.630)	0.006	percentage points
Sex × grade	Boys, Grade 3 change	3.266 (2.284 to 4.248)	<0.001	percentage points
Sex × grade	Boys, Grade 4 change	5.813 (4.846 to 6.781)	<0.001	percentage points
Sex × grade	Boys, Grade 5 change	7.365 (6.331 to 8.398)	<0.001	percentage points
Sex × grade	Boys, Grade 6 change	8.004 (6.877 to 9.130)	<0.001	percentage points
Sex × grade	Girls, Grade 1 change	−0.836 (−2.062 to 0.391)	0.182	percentage points
Sex × grade	Girls, Grade 2 change	0.645 (−0.543 to 1.832)	0.287	percentage points
Sex × grade	Girls, Grade 3 change	2.758 (1.781 to 3.734)	<0.001	percentage points
Sex × grade	Girls, Grade 4 change	3.028 (1.974 to 4.083)	<0.001	percentage points
Sex × grade	Girls, Grade 5 change	4.726 (3.719 to 5.733)	<0.001	percentage points
Sex × grade	Girls, Grade 6 change	5.681 (4.641 to 6.722)	<0.001	percentage points
Grade	Grade 1 change	−0.673 (−1.841 to 0.495)	0.259	percentage points
Grade	Grade 2 change	1.148 (0.075 to 2.222)	0.036	percentage points
Grade	Grade 3 change	3.040 (2.165 to 3.914)	<0.001	percentage points
Grade	Grade 4 change	4.486 (3.599 to 5.372)	<0.001	percentage points
Grade	Grade 5 change	6.099 (5.191 to 7.006)	<0.001	percentage points
Grade	Grade 6 change	6.892 (5.913 to 7.871)	<0.001	percentage points
Contrast	Boys minus girls	1.652 (1.169 to 2.136)	<0.001	percentage points
Contrast	Grade 6 − Grade 5	0.793 (−0.061 to 1.647)	0.069	percentage points
Contrast	Boys versus girls within Grades 4–6	2.591 (1.953 to 3.229)	<0.001	percentage points

Panel A reports the adjusted change in high-BMI prevalence; Panel B reports covariate-adjusted changes in conditional BMI quantiles and formal differences in 2014–2024 change; Panel C reports sex-specific summaries, all 12 sex-by-grade changes, all six grade-specific summaries, and formal contrasts. These analyses included 7,187,779 records from students aged 6.0–12.99 years and 1,394 school codes. High-BMI estimates are 2014–2024 changes in marginally standardized prevalence or differences between these changes. The categorical-year models adjusted for age in months, sex, grade, and district. Age in months was represented by the interval midpoint derived from age in days and modeled using a centered and whitened restricted cubic spline. Quantile estimates describe changes in conditional BMI quantiles rather than changes in marginal population quantiles. Inference used school-cluster-robust covariance; cross-quantile contrasts used joint school-score covariance and were checked with 19,999 school-cluster multiplier replicates. All differences and contrasts were calculated from unrounded model estimates and may differ slightly from values obtained by subtracting displayed rounded estimates. High BMI denotes overweight or obesity; BMI, body mass index; CI, confidence interval.

Figure 2B compares the endpoint sensitivity estimates. Changes in adjusted high-BMI prevalence were 3.29 percentage points under the fixed 2014 standard, 3.34 under the equal-year standard, and 3.51 in the fixed-school panel (95% CI 2.67–4.35; 5,060,353 records from 704 schools), compared with 3.35 in the primary analysis (95% CI 2.69–4.01; Supplementary Tables S4, S9; Supplementary Figure S2).

Including the four school codes yielded 7,259,258 age-eligible records in 1,398 school clusters. With the original standard population held fixed, the 2014–2024 change in adjusted high-BMI prevalence was 3.32 percentage points (95% CI 2.66–3.97), compared with 3.35 (2.69–4.01) in the primary analysis (Supplementary material SM7; Supplementary Table SM6).

Under WHO classification at the September 1 reference age, adjusted high-BMI prevalence increased from 27.36% in 2014 to 30.59% in 2024, a change of 3.23 percentage points (95% CI 2.47–3.98). The increase remained positive in the classification-age and extreme-Z-score sensitivity analyses (Supplementary Tables S16, S17).

### 3.2 Observed and conditional BMI quantile changes

All four reported observed BMI quantiles increased from 2014 to 2024 in the primary analysis sample, with larger increases in the upper tail than at the median. BMI P50 increased from 16.39 kg/m^2^ in 2014 to 16.60 kg/m^2^ in 2024. P75, P90, and P95 increased by 0.76, 1.09, and 1.26 kg/m^2^, respectively. The P95 − P50 difference widened from 6.75 kg/m^2^ to 7.80 kg/m^2^ (Figure 3A; Supplementary Table S10).

**Figure 3:**
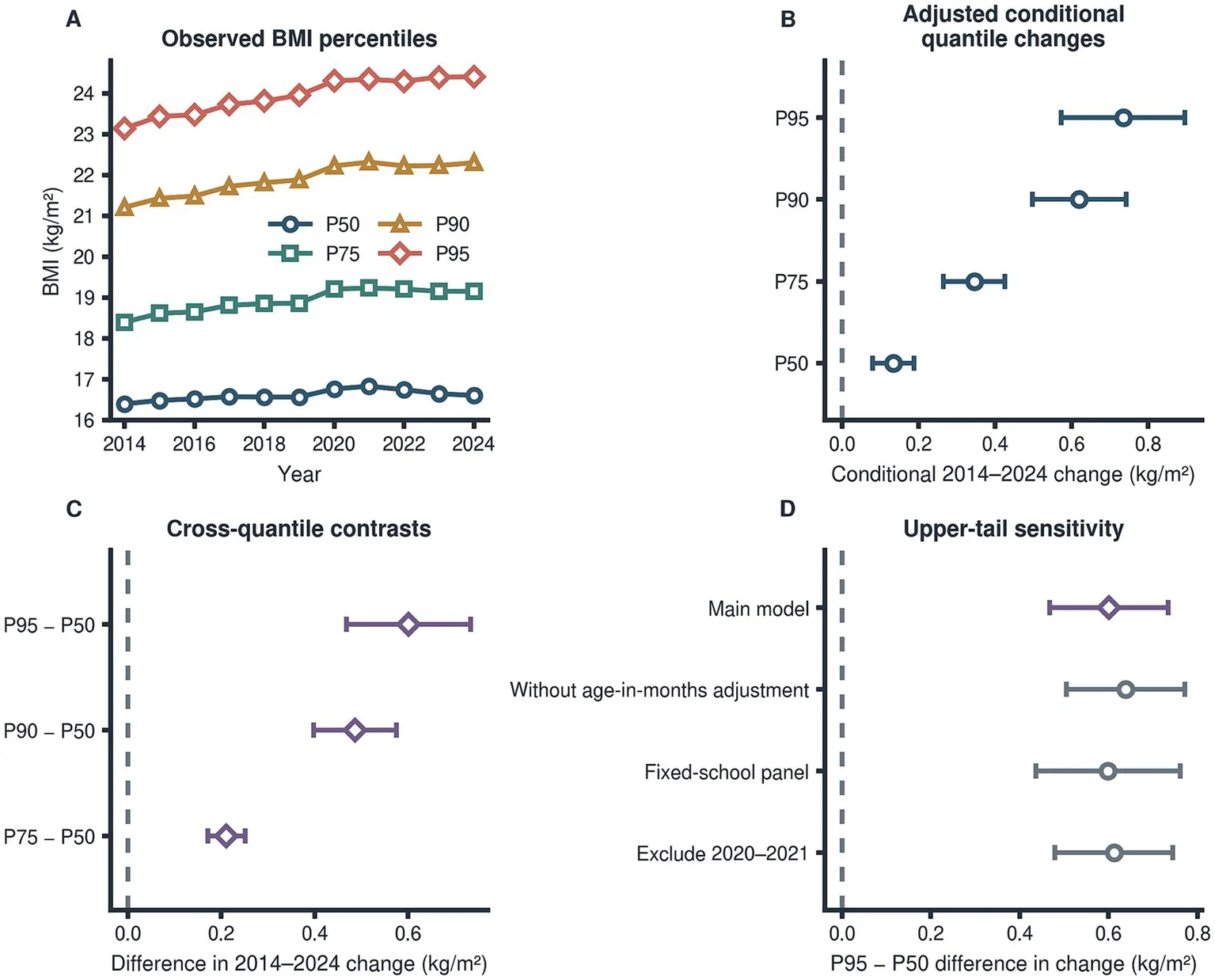
Observed BMI percentiles and covariate-adjusted changes in conditional BMI quantiles, 2014–2024. Panel **(A)** shows observed percentiles in the primary sample (7,187,779 records; 1,394 school codes). Panels **(B,C)** show covariate-adjusted 2014–2024 changes in conditional BMI quantiles and cross-quantile differences in change. Models included categorical year, the age-in-months spline, sex, grade, and district; cross-quantile inference used joint school-score covariance. Panel **(D)** compares the P95 − P50 contrast from the primary model (purple diamond) with sensitivity estimates (gray circles) omitting the age spline, using the fixed-school panel, or excluding 2020–2021. Points are estimates and error bars are school-cluster-robust 95% confidence intervals. Conditional estimates are not marginal population quantiles. BMI, body mass index.

Covariate-adjusted changes in conditional BMI quantiles between 2014 and 2024 were progressively larger from P50 to P95. The estimated changes were 0.134 kg/m^2^ at P50 (95% CI 0.079–0.188), 0.345 kg/m^2^ at P75 (95% CI 0.265–0.425), 0.620 kg/m^2^ at P90 (95% CI 0.498–0.742), and 0.735 kg/m^2^ at P95 (95% CI 0.573–0.896) (Figure 3B; Table 3; Supplementary Table S11). Formal differences in change were 0.211 kg/m^2^ for P75 − P50 (95% CI 0.171–0.252), 0.486 kg/m^2^ for P90 − P50 (95% CI 0.397–0.575), and 0.601 kg/m^2^ for P95 − P50 (95% CI 0.468–0.734); all *p* < 0.001 (Figure 3C; Table 3; Supplementary Table S11). After 2020 and 2021 were jointly excluded, the P95 − P50 difference in 2014–2024 change was 0.612 kg/m^2^ (95% CI 0.479–0.745; *p* < 0.001; 5,727,161 records; 1,391 school codes), compared with 0.601 kg/m^2^ (95% CI 0.468–0.734) in the primary model. The corresponding school-cluster multiplier-bootstrap interval was 0.483–0.744 (Figure 3D; Supplementary Table S4).

The P95 − P50 difference in 2014–2024 change was 0.639 kg/m^2^ (95% CI 0.506–0.772) without age-in-months adjustment and 0.599 kg/m^2^ (95% CI 0.437–0.761) in the fixed-school panel (Figure 3D; Supplementary Tables S4, S11).

In the exploratory lower-quantile analysis, covariate-adjusted changes in conditional BMI quantiles between 2014 and 2024 were 0.097 kg/m^2^ at P5 (95% CI 0.027–0.167), 0.047 kg/m^2^ at P10 (95% CI − 0.008–0.103), and 0.048 kg/m^2^ at P25 (95% CI − 0.002–0.097). The P10 and P25 intervals included zero. The same interval pattern was obtained with observation-level kernel-weighted covariance at all three bandwidth multipliers (Supplementary Table S15; Supplementary material SM8).

The custom quantile-regression fits at all four primary quantiles converged in the 51,480-record internal sample. The maximum relative check-loss difference from HiGHS was 4.02 × 10^−10^, and the maximum absolute difference in the 2024 coefficients was 0.002195 kg/m^2^ (Supplementary material SM3; Supplementary Table SM1). In the separate 200,000-record validation sample, the pairs-refit to linearized standard-error ratio for P95 − P50 was 0.966. In the full-sample kernel-weighted sensitivity analysis, confidence intervals for all four quantile changes and three upper-quantile versus P50 contrasts remained above zero at each bandwidth multiplier (0.75, 1.00, and 1.25; Supplementary material SM3; Supplementary Table SM2). Precision-reduction and terminal-digit sensitivity results are reported in Supplementary Tables S3, S11 and Supplementary Figure S1.

With the four school codes included, the covariate-adjusted changes in conditional BMI quantiles at P50 and P95 were 0.133 and 0.729 kg/m^2^, respectively. The P95 − P50 difference in change was 0.596 kg/m^2^ (95% CI 0.465–0.728); all three upper-quantile versus P50 contrasts remained positive (Supplementary material SM7; Supplementary Table SM6).

### 3.3 Sex- and grade-specific changes in adjusted high-BMI prevalence

Figure 4 summarizes sex- and grade-specific changes in adjusted high-BMI prevalence and targeted contrasts. Categorical-year interaction tests showed that annual changes varied by sex (*F*(10,1,393) = 10.01; *p* < 0.001) and grade (*F*(50,1,393) = 5.50; *p* < 0.001). The year × sex × grade interaction also indicated heterogeneity (*F*(50,1,393) = 2.26; *p* < 0.001; Supplementary Tables S12, S13).

**Figure 4:**
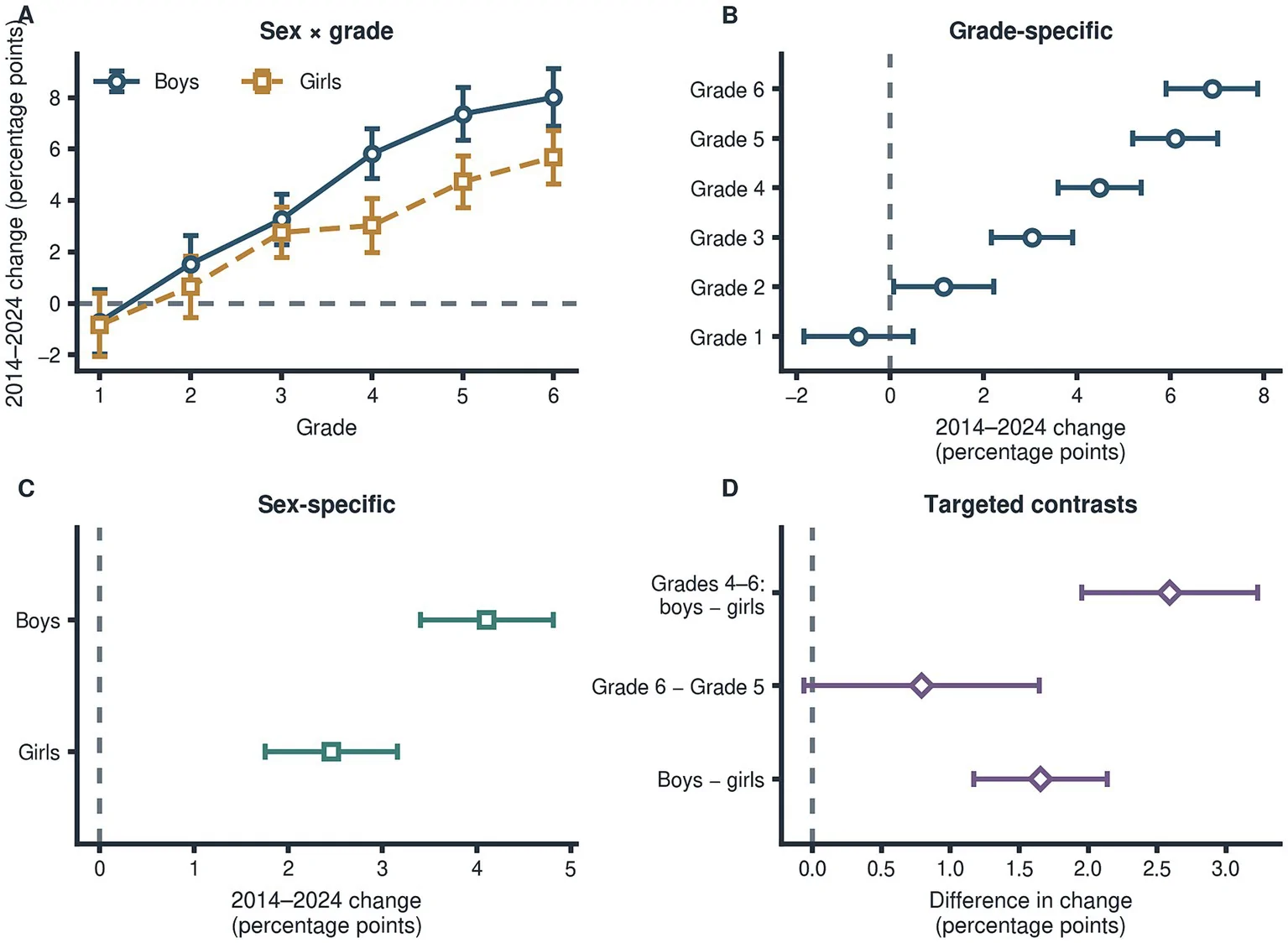
Sex- and grade-specific changes in adjusted high-BMI prevalence, 2014–2024. All panels use the primary analysis sample (7,187,779 records; 1,394 school codes) and categorical-year models adjusted for the centered and whitened age-in-months spline, sex, grade, and district, with school-cluster-robust 95% confidence intervals. Panel **(A)** shows all 12 sex-by-grade changes in adjusted high-BMI prevalence; exact values are also reported in Table 3 and Supplementary Table S12. Panel **(B)** shows grade-specific standardized changes from 2014 to 2024, Panel **(C)** shows sex-specific changes, and Panel **(D)** shows targeted formal differences in change. Grade 6 had the largest point estimate, but the Grade 6 − Grade 5 contrast was 0.793 percentage points (95% CI − 0.061 to 1.647; *p* = 0.069). High BMI denotes overweight or obesity.

The change in adjusted high-BMI prevalence from 2014 to 2024 was 4.11 percentage points among boys (95% CI 3.40–4.82) and 2.46 percentage points among girls (95% CI 1.76–3.16). The increase was 1.65 percentage points greater among boys than among girls (95% CI 1.17–2.14). Within Grades 4–6, the increase was 2.59 percentage points greater among boys than among girls (95% CI 1.95–3.23) (Figures 4C,D; Table 3; Supplementary Tables S12, S13).

Figure 4A displays all 12 sex-by-grade changes, with estimates and 95% confidence intervals reported in Table 3 and Supplementary Table S12.

Combining boys and girls, estimated changes in adjusted high-BMI prevalence for Grades 1–6 were −0.67, 1.15, 3.04, 4.49, 6.10, and 6.89 percentage points, respectively (Figure 4B; Table 3; Supplementary Table S12). The Grade 6 − Grade 5 difference was 0.793 percentage points (95% CI − 0.061 to 1.647; *p* = 0.069), providing no clear evidence of a difference between these adjacent grades (Figure 4D; Supplementary Table S13).

Including the four school codes gave boys−girls differences in the change in adjusted high-BMI prevalence of 1.62 percentage points overall (95% CI 1.14–2.11) and 2.57 within Grades 4–6 (1.93–3.21). The Grade 6 − Grade 5 interval still included zero (Supplementary material SM7; Supplementary Table SM6).

In the WHO sensitivity analysis, the boys−girls difference in the 2014–2024 change in adjusted high-BMI prevalence was 1.38 percentage points overall (95% CI 0.78–1.97) and 2.90 within Grades 4–6 (2.11–3.69). The Grade 6 − Grade 5 difference was 0.44 percentage points (−0.58 to 1.46; Supplementary Table S16).

In the model that represented age in months as a linear term, the boys−girls difference in the change in adjusted high-BMI prevalence was 1.476 percentage points (95% CI 0.994–1.958; *p* < 0.001), and the boys-versus-girls contrast within Grades 4–6 was 2.638 percentage points (95% CI 1.999–3.277; *p* < 0.001). The Grade 6 − Grade 5 contrast was 0.665 percentage points (95% CI − 0.187 to 1.517; *p* = 0.126), so its confidence interval included zero (Supplementary Table S4).

### 3.4 District variation in high-BMI prevalence

Sex-by-grade-standardized high-BMI prevalence varied across districts and over time from 2014 to 2024. District-year estimates and the 2014 and 2024 distributions are reported in Table 4, Supplementary Table S14, and Supplementary Figure S3. District prevalence ranged from 10.75 to 20.25% in 2014 (maximum−minimum difference, 9.50 percentage points) and from 17.05 to 23.30% in 2024 (difference, 6.25 percentage points). Pointwise 95% confidence intervals for endpoint prevalence and changes are reported in Supplementary material SM5 (Supplementary Table SM5). Change intervals included zero for Beilin, Lianhu, Yanliang, and Yanta.

**Table 4:** District-specific sex-by-grade-standardized high-BMI prevalence at the study endpoints.

District	2014 standardized high BMI	2024 standardized high BMI	Change	Observed N (2014; 2024)
Lintong	16.72%	22.57%	+5.85 pp	33,441; 34,908
Zhouzhi	10.75%	17.05%	+6.29 pp	30,681; 30,772
Xincheng	19.19%	22.06%	+2.88 pp	32,549; 36,765
Weiyang	17.83%	23.30%	+5.47 pp	52,741; 152,922
Baqiao	16.16%	21.43%	+5.27 pp	35,899; 88,267
Beilin	19.30%	21.65%	+2.35 pp	34,768; 50,133
Lianhu	19.06%	18.82%	−0.25 pp	42,541; 58,726
Lantian	12.95%	19.28%	+6.33 pp	26,831; 18,486
Huyi	13.54%	19.08%	+5.54 pp	27,180; 32,335
Chang’an	17.40%	21.36%	+3.95 pp	54,644; 122,607
Yanliang	20.25%	21.31%	+1.06 pp	13,398; 19,120
Yanta	18.49%	19.11%	+0.62 pp	67,219; 152,898
Gaoling	18.18%	22.63%	+4.44 pp	13,661; 28,155

District prevalence was directly standardized to the pooled 2014–2024 sex × grade distribution in the primary standardized-reference age sample of 6.0–12.99 years and was not continuously adjusted for standardized-reference age in months. Observed N is the number of eligible surveillance records contributing to each district-year estimate. Changes were calculated from the unrounded standardized estimates and may differ slightly from differences between displayed values. Pointwise school-cluster bootstrap 95% confidence intervals for endpoint prevalence and changes are provided in Supplementary material SM5 (Supplementary Table SM5). The table describes geographic variation only; it does not apply a socioeconomic ranking or support a causal contextual interpretation. pp, percentage points.

### 3.5 Exploratory school-level adjusted high-BMI burden patterns and PE-resource findings

The school-level adjusted burden-pattern analysis classified 43 schools as persistent high burden, 106 as emerging high burden, 93 as declining high burden, 313 as fluctuating/intermittent burden, and 249 as stable low burden. Supplementary Table S5 and Supplementary Figure S4 report category counts under alternative specifications for the minimum number of observed years, persistence proportion, trend threshold, anomalous-year exclusion, and annual P70/P80 high-burden thresholds. Within the age-restricted sample, adding the monthly-age spline changed 24 of 804 school classifications (agreement, 97.01%; κ = 0.959). After both age restriction and monthly-age adjustment, agreement with the original classification was 96.27% (Supplementary material SM4; Supplementary Tables SM3, SM4).

In the 2024 PE-resource analysis, a 1-SD higher average class size was associated with a 1.44-percentage-point higher adjusted burden residual in the weighted linear model (95% CI 0.23 to 2.65; BH q = 0.155), and the corresponding high-burden odds ratio (OR) was 1.50 (95% CI 1.01 to 2.24; q = 0.166). Across all eight tested resource-exposure associations, BH q values ranged from 0.155 to 0.647 and Holm-adjusted *p* values from 0.155 to 1.000; none met the 0.05 threshold under either correction. The confidence intervals are unadjusted for multiple testing. Model-specific denominators, event counts, and QC sensitivity results are reported in Supplementary Table S6 and Supplementary Figure S5.

## 4 Discussion

### 4.1 Principal findings and contribution

Across 11 consecutive years of surveillance in participating primary schools in Xi’an, observed high-BMI prevalence in the primary sample increased by 4.09 percentage points. The increase in adjusted high-BMI prevalence was 3.35 percentage points (95% CI 2.69–4.01), and the fixed-school adjusted increase was 3.51 percentage points (95% CI 2.67–4.35). The adjusted estimates compare years at a common measured covariate distribution; their difference from the observed change does not quantify the causal contribution of sample composition. Covariate-adjusted changes in conditional BMI quantiles were 0.735 kg/m^2^ at P95 and 0.134 kg/m^2^ at P50, yielding a P95 − P50 difference in 2014–2024 change of 0.601 kg/m^2^. Changes in adjusted high-BMI prevalence varied by sex and grade. The increase was greater among boys than girls overall and within Grades 4–6.

Together, the prevalence, conditional-quantile, and subgroup findings describe complementary aspects of BMI change across 11 consecutive annual surveillance waves. Formal comparisons of changes at P75, P90, and P95 with the change at P50 quantify how much larger the increases were in the upper conditional distribution, while sex-by-grade estimates locate differences in prevalence trends within the participating-school records.

### 4.2 Interpretation of upper-tail and sex-by-grade differences

Earlier Chinese research documented secular rightward shifts and changes in BMI-distribution dispersion during 1985–2010 (6). A study of urban children in Changsha compared 2002 with 2018 and reported larger changes in upper percentiles than near the distributional center (8). Those studies support nonuniform change but differ from the present analysis in geography, study period, and number of observation years. The conditional quantile findings in Xi’an were directionally consistent with this earlier evidence: covariate-adjusted changes in conditional BMI quantiles were 0.134 kg/m^2^ at P50, 0.620 kg/m^2^ at P90, and 0.735 kg/m^2^ at P95. These changes in conditional BMI quantiles differ from changes in observed marginal population percentiles. The exploratory lower-quantile estimates indicated a small increase at P5; changes at P10 and P25 remained uncertain because their intervals included zero.

National studies provide useful context, although their estimates are not directly comparable with ours. Song et al. reported an age-standardized combined overweight-and-obesity prevalence of 21.5% (95% CI 21.3–21.7) among Chinese Han students aged 7–18 years in 2019 (17), whereas observed high-BMI prevalence in the present Xi’an primary-school sample was 20.19% in 2019. The latter is unadjusted, covers a narrower grade and age range, and is municipal rather than nationally representative. Among Chinese adults aged 18–69 years, nationally representative surveys from 2004 to 2018 showed an increase in standardized mean BMI from 22.7 to 24.4 kg/m^2^ and in obesity defined as BMI ≥ 30 kg/m^2^ from 3.1 to 8.1% (36). Global estimates also show increasing obesity prevalence in both school-aged populations and adults (1). Together, these sources indicate increases in BMI-based population measures in both age groups, but differences in periods, ages, geography, classification thresholds, and standardization preclude a valid claim that childhood obesity increased faster than adult obesity.

From 2014 to 2024, mean age increased slightly, the proportion of boys declined, and the grade composition shifted toward Grades 5–6 (Table 2). The adjusted endpoint increase in high-BMI prevalence was 0.75 percentage points smaller than the unadjusted primary-sample increase. The fixed-school adjusted increase was 3.51 percentage points (95% CI 2.67–4.35), compared with 3.35 percentage points (95% CI 2.69–4.01) in the primary analysis. The adjusted and fixed-school endpoint estimates remained positive after accounting for measured covariates or restricting the school set. However, these comparisons do not determine the contribution of unmeasured changes in participating students, reporting, migration, or other aspects of sample composition and should not be interpreted as a causal decomposition.

High-BMI prevalence and conditional quantiles answer different surveillance questions. The adjusted 3.35-percentage-point prevalence increase describes the change in the proportion classified as overweight or obese under fixed national cut-points. The covariate-adjusted conditional quantile estimates show larger BMI increases at P90 and P95 than at P50, with a P95 − P50 difference in change of 0.601 kg/m^2^. These are distributional positions rather than additional weight-status thresholds. An omnibus distributional test addresses whether distributions differ, whereas the reported contrasts quantify adjusted changes at specified quantiles with school-clustered uncertainty. Prevalence changes quantify shifts in classification frequency, whereas conditional quantile changes quantify shifts in BMI; their magnitudes cannot be compared directly. BMI remains a screening measure rather than a direct measure of adiposity or metabolic status (37).

Adjusted high-BMI prevalence increased by 4.11 percentage points among boys and 2.46 percentage points among girls, a difference in change of 1.65 percentage points (95% CI 1.17–2.14). Within Grades 4–6, the boys−girls difference was 2.59 percentage points (95% CI 1.95–3.23). This direction is consistent with Chinese National Surveys on Students’ Constitution and Health data from 1985 to 2010: the sex-specific trend rate ratio for obesity was 1.59 (95% CI 1.58–1.60) among boys and 1.49 (95% CI 1.48–1.50) among girls, with the fastest increase among rural boys (18).

A faster increase among boys should not, however, be treated as universal across Chinese settings or periods. Using criteria from the Working Group on Obesity in China, a 15-province survey in 2015 reported higher overweight and obesity prevalence among boys (14.0 and 10.5%) than among girls (9.7 and 7.1%). Its 2011–2015 trend analysis across 12 provinces found no significant overall change in overweight or obesity prevalence, although obesity increased among adolescents aged 12–18 years (19). Differences in study period, population, and classification criteria may account for these age patterns. The Xi’an difference in change cannot be ranked directly against national trend rate ratios or cross-sectional prevalence gaps because the study periods, age ranges, sampling frames, classification rules, and estimands differ. Developmental stage, physical activity, sedentary behavior, and school environments are potential explanations for the sex and grade differences (38–40). These factors were not measured in this study, so their roles remain hypotheses. Because the design was repeated cross-sectional, the findings describe changes across annual student populations, not within-child trajectories.

WHO classification yielded higher high-BMI prevalence than the national standard, reflecting differences between the sex- and age-specific WHO reference and the sex- and grade-specific national cut-points. The overall absolute prevalence increases were similar, and the boys−girls differences in prevalence change remained positive under both classifications. Thus, classification affected the prevalence level without changing the direction of these findings. This consistency does not establish equivalence between the standards. Grade-specific estimates describe school-stage differences; adjustment for age in months does not turn them into age-stratified analyses or individual growth trajectories.

Future studies could examine broadband access alongside individual internet use, diet, and physical activity, building on previous research (41–43); these exposures were not measured in the present surveillance records.

The annual pattern was nonlinear rather than a steady decade-long increase. Adjusted high-BMI prevalence increased from 20.25% in 2019 to 22.75% in 2020, a change of 2.51 percentage points calculated from the unrounded estimates. It remained elevated at 22.40% in 2021 and then declined to 20.75% in 2024, 2.00 percentage points below the 2020 peak. Other Chinese studies provide context, although their populations and designs differ. In a two-year longitudinal study of 6,047 Shanghai adolescents aged 11–16 years examined in September–November 2019 and 2020, obesity prevalence increased from 14.2 to 15.4% and mean BMI from 20.3 to 21.2 kg/m^2^. The proportion reporting at least 60 min of moderate-to-vigorous physical activity on all seven days decreased from 14.4 to 11.7%, while mobile screen time increased (44). A retrospective online survey of 10,082 older Chinese youths (mean age 19.8 years) reported an increase in mean BMI from 21.8 to 22.1 kg/m^2^ and in overweight/obesity prevalence from 21.4 to 24.6%. The same survey reported less physical activity and more sedentary and screen time after lockdown (45).

These external studies differed from the present analysis in age, location, sampling, outcome definition, and design and therefore cannot identify the cause of the Xi’an pattern. The surveillance records did not measure school-closure or online-schooling exposure, diet, physical activity, screen time, infection, or contemporaneous changes in testing and reporting procedures. After 2020 and 2021 were jointly excluded, the P95 − P50 difference in 2014–2024 conditional BMI change was 0.612 kg/m^2^ (95% CI 0.479–0.745), compared with 0.601 kg/m^2^ (95% CI 0.468–0.734) in the primary model. The upper-tail contrast persisted after excluding 2020 and 2021. This sensitivity analysis neither confirms nor rules out pandemic-related mechanisms; the annual pattern alone does not establish a pandemic effect.

### 4.3 Secondary district and exploratory school findings

Standardized high-BMI prevalence differed across districts at both endpoints. The maximum–minimum difference was 9.50 percentage points in 2014 and 6.25 percentage points in 2024. We did not formally test the change in this difference. All 13 district-specific endpoint estimates are reported in Table 4 and Supplementary Table S14, with confidence intervals for endpoint prevalence and changes in Supplementary material SM5 (Supplementary Table SM5). Because verified and comparable district-level socioeconomic indicators were unavailable, these geographic differences were not interpreted as changes in socioeconomic concentration or health equity.

The district analysis describes geographic variation without identifying its causes. The school-level adjusted high-BMI burden-pattern analysis described relative contemporaneous burden over time, but its categories depended on an annual percentile threshold and should not be interpreted as forecasts or diagnoses. The 2024 PE-resource analysis was cross-sectional and exploratory. Resources measured in 2024 may not represent cumulative exposure during 2014–2024. Administrative counts do not capture actual availability or use, facility quality or accessibility, PE lesson content or delivery, or individual physical activity. Residual confounding and exposure misclassification therefore remain likely. None of the eight PE-resource associations met the 0.05 threshold after either BH-FDR or Holm correction. This indicates uncertainty under the fitted models, not evidence that school PE environments are unrelated to childhood BMI. Future studies should measure both resources and their use repeatedly and link them to behavior.

The present analyses do not determine whether increases in BMI were greater in schools or districts that were already heavier in 2014. Addressing this question would require a prespecified baseline definition and methods that account for mathematical coupling and regression to the mean. It should therefore be examined in a dedicated future analysis.

### 4.4 Limitations

The repeated cross-sectional design estimates changes among annual tested populations, not within-child trajectories or causal effects. Unique longitudinal student identifiers were unavailable, so repeated observations of the same child across years could not be identified. Participation and reporting were not independently audited, and the lack of comparable eligible-school and eligible-student denominators prevented estimation of annual coverage. Standardization accounts for measured covariate composition, and the fixed-school analysis assesses sensitivity to changes in the participating school set. Selection bias from incomplete reporting, differential testing participation, or unmeasured composition may remain. The findings therefore apply to surveillance records from participating primary schools in Xi’an and should not be assumed to represent all children in Xi’an, children outside school, or other settings.

Anthropometry followed the national protocol and BMI was recalculated from recorded height and weight by the research team, but site- and year-specific calibration, transcription, rounding, digit preference, and protocol adherence could not be independently audited. Coarsened-measurement and terminal-digit sensitivity analyses were reassuring but cannot exclude all measurement or reporting error. BMI is a screening measure rather than a direct measure of adiposity, body composition, pubertal maturation, or metabolic risk. The national framework places physical-health results in student and school monitoring records and incorporates testing, reporting, pass rates, excellence rates, and health trends into school monitoring and evaluation (24, 25). These evaluation requirements may create non-financial institutional incentives concerning reported physical-health performance. We identified no rule allocating funding according to BMI levels and did not assess actual strategic testing or reporting behavior. Recalculating BMI cannot correct selective testing or reporting, or bias in the recorded height and weight; such bias therefore cannot be excluded.

Exact individual test dates were unavailable. The September 1 reference therefore underestimated age at measurement by 44–75 days and may have misclassified some records near the 6.0–12.99-year eligibility boundaries or left residual age-at-measurement variation within grades. The WHO classification scenarios assessed sensitivity to the two testing-window endpoints with eligibility and model covariates held fixed; they could not recover actual measurement ages or quantify eligibility misclassification. Individual socioeconomic circumstances, diet, physical activity, screen time, sleep, pubertal status, migration, internet exposure, and pandemic-related schooling were also unavailable. These omissions limit the assessment of mechanisms and control of residual confounding; temporal and subgroup associations should not be interpreted as effects of any specific exposure.

The district, school-pattern, and PE-resource analyses were secondary or exploratory. District prevalence was standardized by sex and grade but not continuously by standardized-reference age in months, and only 13 ecological units were available; geographic differences cannot be interpreted at the individual level or as causal contextual effects. School burden-pattern classes depend on thresholds for minimum records, observation history, early and late periods, and 70% persistence. School classifications also depended on monthly-age adjustment, as shown by the sensitivity analysis. The original PE-resource models remained unadjusted for within-grade age composition. Finally, PE resources were measured only in 2024, and administrative counts do not measure historical exposure, actual use, quality, accessibility, lesson content, or student activity. Residual confounding and multiple testing remain, and the absence of multiplicity-adjusted associations is not evidence that school PE environments are unrelated to childhood BMI. The sensitivity analysis including the four district-switch school codes assessed primary and subgroup estimates; it did not reassess district summaries, school burden classifications, or PE-resource associations.

### 4.5 Implications for surveillance and public health practice

School health surveillance could report high-BMI prevalence alongside covariate-adjusted changes in conditional BMI quantiles at P90 and P95 relative to P50, together with sex- and grade-specific prevalence changes. These complementary measures can show whether a rising prevalence is accompanied by larger increases in the upper conditional distribution and how prevalence trends differ across population subgroups. Estimates should be accompanied by confidence intervals.

Repeated district- and school-level summaries could supplement sex- and grade-specific surveillance and help identify areas and schools that require further assessment. Policies addressing food and nutrition environments generally require coordination across multiple levels (46). Linking surveillance to health-promoting school standards aligns with existing global frameworks (47), and a systematic review provides some evidence for whole-school health promotion (48). Reviews of school-based physical activity and childhood obesity prevention show that intervention effects are not consistent across all outcomes and settings (49, 50). The present surveillance findings can help identify groups for further assessment and provide comparative baselines. They do not establish intervention effectiveness.

## 5 Conclusion

Among annual surveillance records from participating primary schools in Xi’an, adjusted high-BMI prevalence increased by 3.35 percentage points (95% CI 2.69–4.01) between 2014 and 2024. Covariate-adjusted changes in conditional BMI quantiles were greater at P95 than at P50 (0.735 versus 0.134 kg/m^2^), with a difference in change of 0.601 kg/m^2^ (95% CI 0.468–0.734). Adjusted high-BMI prevalence increased more among boys than girls overall and within Grades 4–6. Joint reporting of high-BMI prevalence, changes in upper conditional BMI quantiles, and sex- and grade-specific estimates may provide a more complete picture of BMI trends in participating schools. District comparisons were descriptive, and the school burden-pattern and 2024 PE-resource analyses were exploratory.

## Code availability statement

Custom analysis scripts and non-identifying validation outputs are provided in the supplementary code archive, Supplementary_Analysis_Code_R3_Complete.zip. The archive documents the grouped quantile-regression, school-cluster covariance, validation, and school burden-classification procedures and their software requirements. The archive separates recovered analyses from the additional quantile validation, covariance sensitivity, school-classification age sensitivity, district bootstrap analyses, and the four-school inclusion sensitivity analysis. Full record-level reproduction requires authorized access to the restricted surveillance data and protected school-level inputs described in the archive README; these inputs are not included in the public materials. The lower-quantile and WHO classification extensions, validation outputs, and reference sources are documented in README_REVIEWER3_COMPLETE.md.

## Data Availability

Aggregate data underlying the main tables and figures, data-cleaning summaries, model outputs, and figure source values are provided in the Supplementary Material and Supplementary Source Data. These files contain no student-level records or school identifiers. Individual-level surveillance records and school-level analytic data are not publicly available because they contain sensitive health information about minors and potentially identifying information. Requests for access to the restricted de-identified data should be directed to Bing Shi (18092713165@163.com). Access requires authorization from the relevant data custodian, compliance with applicable ethics requirements, and a data-use agreement prohibiting re-identification and onward sharing.
