# Supplementary material for: BMI distribution and sex- and grade-specific trends in primary school health surveillance records in Xi’an, China, 2014–2024: a repeated cross-sectional study
Source: Front Public Health. 2026 Oct 28;14:1942237. doi: 10.3389/fpubh.2026.1942237 (PMC13752098; doi:10.3389/fpubh.2026.1942237)
Supplement: Supplementary file 1 [file Data_Sheet_1.PDF]

Supplementary Material

Tables S1–S17, Figures S1–S5, Supplementary Methods SM1–SM9, and Tables SM1–SM7

Source-data workbook: Supplementary_Source_Data_Stage7.xlsx; additional four-school inclusion outputs are provided as CSV files in Supplementary_Analysis_Code_R3_Complete.zip (stage8_inclusion_outputs). Lower-quantile estimates and validation outputs are provided as CSV files in the same archive (reviewer3_lower_outputs). WHO classification outputs are in reviewer3_who_outputs.

All analytical values use the result set after exclusion of the four schools with district-field switches except for the explicitly labeled pre-exclusion raw-file quality-control audit and the four-school inclusion sensitivity analysis in SM7 and Table SM6. Tables and figures are grouped by type; analyses added during revision are appended to the relevant table and methods series. This reduced-scope revision excludes the former source-dependent socioeconomic-gradient and ecological analyses, the split-sample baseline-versus-change analysis, previously reported alternative age-reference outputs, and fixed-threshold or empirical-distribution outputs because their primary-source or executable evidence chains were incomplete. The retained Supplementary Material and Supplementary Source Data contain only the synchronized evidence used in the reduced-scope manuscript. Supplementary Methods SM1–SM9 and Tables SM1–SM7 provide the statistical specifications and additional validation and sensitivity analyses for this revision. The new WHO classification-age scenarios in SM9 were computed from the verified primary sample and are separate from those historical outputs.

Contents

Supplementary Table S1. Sample construction and analytic branches
Supplementary Table S2. Raw-file measurement consistency and protocol audit
Supplementary Table S3. Terminal-digit and conservative measurement-resolution sensitivity
Supplementary Table S4. Primary-model sensitivity and diagnostics, including joint exclusion of 2020–2021
Supplementary Table S5. School-level adjusted high-BMI burden-pattern classification and stability
Supplementary Table S6. 2024 school PE-resource exploration
Supplementary Table S7. Annual observed nutritional status
Supplementary Table S8. Adjusted annual high-BMI prevalence and assessment of nonlinearity
Supplementary Table S9. Fixed-panel representation of retained records and school codes
Supplementary Table S10. Annual observed BMI percentiles
Supplementary Table S11. Conditional quantile results and internal validation
Supplementary Table S12. Adjusted high-BMI prevalence changes for all sex, grade, and sex-by-grade groups
Supplementary Table S13. Interaction tests and targeted prevalence-change contrasts
Supplementary Table S14. District-year standardized high-BMI prevalence
Supplementary Table S15. Exploratory changes in lower conditional BMI quantiles, 2014–2024.

Supplementary Table S16. WHO classification sensitivity: endpoint changes and targeted contrasts.

Supplementary Table S17. Annual high-BMI prevalence under national and WHO classifications.

Supplementary Figure S1. Conservative measurement-resolution sensitivity for the change in adjusted high-BMI prevalence and the conditional P95−P50 difference in 2014–2024 change.
Supplementary Figure S2. Fixed-panel representation and observed outcomes in all schools and the fixed 704-school panel.
Supplementary Figure S3. District patterns of sex-by-grade-standardized high-BMI prevalence, 2014–2024.
Supplementary Figure S4. School-level adjusted high-BMI burden-pattern classification and one-factor stability analyses.
Supplementary Figure S5. Exploratory 2024 school physical-education-resource associations.
Supplementary Methods SM1–SM9. Statistical specifications, validation, and sensitivity analyses

Tables SM1–SM2. Expanded quantile validation and residual-density sensitivity

Tables SM3–SM4. School classification sensitivity to monthly-age adjustment

Table SM5. District endpoint estimates and confidence intervals

Table SM6. Primary and subgroup changes with and without the four school codes

Table SM7. Internal numerical validation of P5, P10, and P25

Supplementary Table S1. Sample construction and analytic branches

The branch denominators differ and are not nested. The main analysis uses 7,187,779 records aged 6.0–12.99 years in 1,394 school clusters.

Results

Flow item	Records / schools	Reason / interpretation
Original records	7,278,755	All 143 district-year files
Primary-grade records	7,276,024	2,731 non-primary-grade records excluded
QC-valid before administrative exclusion	7,271,604	Original QC sample
Administratively excluded school records	71,532	Four codes changed district field at the 2019–2020 boundary
Final cleaned records	7,200,072	1,394 school codes
Unparseable birth-date exclusions	1	Removed from primary age-adjusted analyses
Outside 6.0–12.99 years	12,292	Removed from primary age-adjusted analyses
Primary age-restricted records	7,187,779	Main high-BMI, QR and subgroup analyses
Fixed panel	704	5,060,353 age-restricted records
Adjusted high-BMI burden-pattern-eligible schools	804	Exploratory
2024 PE-resource matched schools	951	Exploratory

Supplementary Table S2. Raw-file measurement consistency and protocol audit

Pre-administrative-exclusion audit of 143 raw district-year files; these quality-control denominators do not define an analytic sample.

Annual raw-file measurement audit (column block 1 of 3; first two columns repeated)

year	raw records	non primary excluded	qc valid records	unparseable birth dates	age valid records	outside 6 12 99	age restricted records	bmi diff gt 0 5	signature collision rows
2014	476,411	0	475,913	0	475,913	4,671	471,242	0	905
2015	511,401	1,546	509,588	1	509,587	1,089	508,498	0	963
2016	516,882	5	516,526	0	516,526	968	515,558	0	1,159
2017	561,950	0	561,630	1	561,629	751	560,878	0	1,088
2018	619,624	106	619,098	1	619,097	706	618,391	0	1,267
2019	669,329	0	669,009	1	669,008	386	668,622	0	1,209
2020	719,984	0	719,569	1	719,568	690	718,878	0	1,589
2021	756,627	43	756,185	0	756,185	323	755,862	0	1,542
2022	785,073	0	784,677	0	784,677	1,222	783,455	0	1,777
2023	827,251	0	826,575	0	826,575	629	825,946	0	2,172
2024	834,223	1,031	832,834	0	832,834	906	831,928	0	1,711

Annual raw-file measurement audit (column block 2 of 3; first two columns repeated)

year	raw records	signature duplicate excess	year field mismatch	height mean cm	height sd cm	weight mean kg	weight sd kg	bmi mean kg m2	bmi sd kg m2
2014	476,411	454	0	134.043	11.563	31.182	9.404	17.058	3.208
2015	511,401	482	0	135.084	11.573	31.994	9.735	17.217	3.228
2016	516,882	581	0	135.375	11.628	32.173	9.860	17.234	3.243
2017	561,950	545	0	135.707	11.756	32.547	10.083	17.343	3.319
2018	619,624	634	0	135.773	11.919	32.638	10.326	17.354	3.361
2019	669,329	606	0	136.183	11.993	32.912	10.572	17.386	3.434
2020	719,984	804	0	136.760	12.064	33.613	10.835	17.601	3.498
2021	756,627	772	0	137.546	12.141	34.112	10.930	17.656	3.458
2022	785,073	889	0	138.180	12.287	34.288	11.041	17.581	3.478
2023	827,251	1,087	0	138.183	12.500	34.204	11.300	17.515	3.551
2024	834,223	857	0	138.629	12.455	34.345	11.334	17.475	3.536

Annual raw-file measurement audit (column block 3 of 3; first two columns repeated)

year	raw records	height integer pct	height half unit pct	weight integer pct	weight half unit pct
2014	476,411	91.243	94.621	84.812	89.553
2015	511,401	90.948	94.822	80.289	86.029
2016	516,882	91.997	95.415	80.779	86.362
2017	561,950	90.484	93.863	79.224	84.849
2018	619,624	89.479	93.680	76.900	83.118

year	raw records	height integer pct	height half unit pct	weight integer pct	weight half unit pct
2019	669,329	89.592	93.772	77.464	83.390
2020	719,984	89.626	93.889	74.982	80.766
2021	756,627	89.175	94.391	74.499	80.285
2022	785,073	89.871	93.802	76.705	82.093
2023	827,251	90.808	94.854	78.861	83.634
2024	834,223	86.359	92.118	71.218	76.931

Annual terminal-digit distribution

year	measure	tenths digit	records	percent
2014	height	0	434,238	91.243
2014	height	1	3,555	0.747
2014	height	2	5,036	1.058
2014	height	3	4,013	0.843
2014	height	4	3,034	0.638
2014	height	5	16,075	3.378
2014	height	6	3,156	0.663
2014	height	7	2,678	0.563
2014	height	8	2,662	0.559
2014	height	9	1,466	0.308
2014	weight	0	403,633	84.812
2014	weight	1	6,356	1.336
2014	weight	2	7,603	1.598
2014	weight	3	6,456	1.357
2014	weight	4	6,514	1.369
2014	weight	5	22,562	4.741
2014	weight	6	6,721	1.412
2014	weight	7	5,522	1.160
2014	weight	8	6,013	1.263
2014	weight	9	4,533	0.952
2015	height	0	463,460	90.948
2015	height	1	3,890	0.763
2015	height	2	4,228	0.830
2015	height	3	3,610	0.708
2015	height	4	3,194	0.627
2015	height	5	19,739	3.874
2015	height	6	3,326	0.653
2015	height	7	2,931	0.575
2015	height	8	2,859	0.561

year	measure	tenths digit	records	percent
2015	height	9	2,351	0.461
2015	weight	0	409,143	80.289
2015	weight	1	9,107	1.787
2015	weight	2	9,782	1.920
2015	weight	3	9,326	1.830
2015	weight	4	8,896	1.746
2015	weight	5	29,250	5.740
2015	weight	6	9,236	1.812
2015	weight	7	8,606	1.689
2015	weight	8	8,711	1.709
2015	weight	9	7,531	1.478
2016	height	0	475,191	91.997
2016	height	1	3,543	0.686
2016	height	2	3,837	0.743
2016	height	3	3,016	0.584
2016	height	4	2,905	0.562
2016	height	5	17,654	3.418
2016	height	6	3,059	0.592
2016	height	7	2,567	0.497
2016	height	8	2,733	0.529
2016	height	9	2,021	0.391
2016	weight	0	417,242	80.779
2016	weight	1	9,461	1.832
2016	weight	2	9,494	1.838
2016	weight	3	8,840	1.711
2016	weight	4	8,785	1.701
2016	weight	5	28,839	5.583
2016	weight	6	9,043	1.751
2016	weight	7	8,309	1.609
2016	weight	8	8,689	1.682
2016	weight	9	7,824	1.515
2017	height	0	508,185	90.484
2017	height	1	4,603	0.820
2017	height	2	5,145	0.916
2017	height	3	4,133	0.736
2017	height	4	4,312	0.768
2017	height	5	18,976	3.379
2017	height	6	4,707	0.838

year	measure	tenths digit	records	percent
2017	height	7	3,946	0.703
2017	height	8	4,373	0.779
2017	height	9	3,250	0.579
2017	weight	0	444,945	79.224
2017	weight	1	10,816	1.926
2017	weight	2	11,341	2.019
2017	weight	3	10,940	1.948
2017	weight	4	10,346	1.842
2017	weight	5	31,591	5.625
2017	weight	6	11,073	1.972
2017	weight	7	10,428	1.857
2017	weight	8	10,701	1.905
2017	weight	9	9,449	1.682
2018	height	0	553,961	89.479
2018	height	1	5,266	0.851
2018	height	2	5,517	0.891
2018	height	3	4,905	0.792
2018	height	4	4,858	0.785
2018	height	5	26,009	4.201
2018	height	6	4,986	0.805
2018	height	7	4,768	0.770
2018	height	8	4,758	0.769
2018	height	9	4,070	0.657
2018	weight	0	476,089	76.900
2018	weight	1	13,234	2.138
2018	weight	2	13,813	2.231
2018	weight	3	13,269	2.143
2018	weight	4	12,829	2.072
2018	weight	5	38,491	6.217
2018	weight	6	13,675	2.209
2018	weight	7	12,660	2.045
2018	weight	8	13,133	2.121
2018	weight	9	11,905	1.923
2019	height	0	599,377	89.592
2019	height	1	5,409	0.809
2019	height	2	6,181	0.924
2019	height	3	5,482	0.819
2019	height	4	5,346	0.799

year	measure	tenths digit	records	percent
2019	height	5	27,965	4.180
2019	height	6	5,474	0.818
2019	height	7	4,667	0.698
2019	height	8	4,993	0.746
2019	height	9	4,115	0.615
2019	weight	0	518,242	77.464
2019	weight	1	14,005	2.093
2019	weight	2	14,481	2.165
2019	weight	3	14,224	2.126
2019	weight	4	13,848	2.070
2019	weight	5	39,642	5.925
2019	weight	6	14,440	2.158
2019	weight	7	13,625	2.037
2019	weight	8	13,960	2.087
2019	weight	9	12,542	1.875
2020	height	0	644,921	89.626
2020	height	1	6,036	0.839
2020	height	2	6,759	0.939
2020	height	3	5,732	0.797
2020	height	4	5,468	0.760
2020	height	5	30,678	4.263
2020	height	6	5,398	0.750
2020	height	7	5,075	0.705
2020	height	8	5,008	0.696
2020	height	9	4,494	0.625
2020	weight	0	539,549	74.982
2020	weight	1	17,511	2.434
2020	weight	2	18,461	2.566
2020	weight	3	17,787	2.472
2020	weight	4	17,288	2.403
2020	weight	5	41,617	5.784
2020	weight	6	17,906	2.488
2020	weight	7	16,762	2.329
2020	weight	8	16,878	2.346
2020	weight	9	15,810	2.197
2021	height	0	674,326	89.175
2021	height	1	5,351	0.708
2021	height	2	6,157	0.814

year	measure	tenths digit	records	percent
2021	height	3	5,468	0.723
2021	height	4	5,442	0.720
2021	height	5	39,441	5.216
2021	height	6	5,432	0.718
2021	height	7	5,090	0.673
2021	height	8	5,063	0.670
2021	height	9	4,415	0.584
2021	weight	0	563,353	74.499
2021	weight	1	18,606	2.461
2021	weight	2	19,617	2.594
2021	weight	3	19,165	2.534
2021	weight	4	18,426	2.437
2021	weight	5	43,752	5.786
2021	weight	6	19,401	2.566
2021	weight	7	18,256	2.414
2021	weight	8	18,644	2.466
2021	weight	9	16,965	2.243
2022	height	0	705,199	89.871
2022	height	1	6,514	0.830
2022	height	2	6,812	0.868
2022	height	3	6,214	0.792
2022	height	4	6,130	0.781
2022	height	5	30,845	3.931
2022	height	6	6,104	0.778
2022	height	7	5,682	0.724
2022	height	8	5,909	0.753
2022	height	9	5,268	0.671
2022	weight	0	601,886	76.705
2022	weight	1	17,370	2.214
2022	weight	2	18,684	2.381
2022	weight	3	17,846	2.274
2022	weight	4	17,374	2.214
2022	weight	5	42,282	5.388
2022	weight	6	18,449	2.351
2022	weight	7	17,446	2.223
2022	weight	8	17,639	2.248
2022	weight	9	15,701	2.001
2023	height	0	750,598	90.808

year	measure	tenths digit	records	percent
2023	height	1	5,469	0.662
2023	height	2	6,194	0.749
2023	height	3	5,629	0.681
2023	height	4	5,531	0.669
2023	height	5	33,445	4.046
2023	height	6	5,328	0.645
2023	height	7	4,975	0.602
2023	height	8	4,929	0.596
2023	height	9	4,477	0.542
2023	weight	0	651,848	78.861
2023	weight	1	16,688	2.019
2023	weight	2	18,101	2.190
2023	weight	3	17,373	2.102
2023	weight	4	16,961	2.052
2023	weight	5	39,450	4.773
2023	weight	6	17,616	2.131
2023	weight	7	16,424	1.987
2023	weight	8	16,971	2.053
2023	weight	9	15,143	1.832
2024	height	0	719,231	86.359
2024	height	1	8,042	0.966
2024	height	2	9,779	1.174
2024	height	3	8,991	1.080
2024	height	4	8,685	1.043
2024	height	5	47,955	5.758
2024	height	6	8,833	1.061
2024	height	7	7,476	0.898
2024	height	8	7,539	0.905
2024	height	9	6,303	0.757
2024	weight	0	593,130	71.218
2024	weight	1	23,823	2.860
2024	weight	2	25,973	3.119
2024	weight	3	25,680	3.083
2024	weight	4	24,714	2.967
2024	weight	5	47,581	5.713
2024	weight	6	24,683	2.964
2024	weight	7	22,984	2.760
2024	weight	8	23,399	2.810

year	measure	tenths digit	records	percent
2024	weight	9	20,867	2.506

School-year jump screen summary

to year	adjacent school transitions n ge 50	median abs mean bmi change	p95 abs mean bmi change	p99 abs mean bmi change	maximum abs mean bmi change	transitions above pooled p99 5
2015	964	0.452	2.221	4.257	8.234	10
2016	962	0.368	1.679	3.626	6.607	6
2017	930	0.376	1.638	3.519	16.204	5
2018	910	0.352	1.436	2.787	16.913	4
2019	911	0.361	1.573	2.822	15.357	4
2020	919	0.433	1.636	2.933	16.544	4
2021	948	0.431	1.868	3.057	4.903	2
2022	934	0.407	1.509	2.741	4.964	1
2023	920	0.353	1.559	3.362	10.817	4
2024	923	0.373	1.604	3.580	8.851	7

Supplementary Table S3. Terminal-digit and conservative measurement-resolution sensitivity

Post-exclusion analyses. Precision coarsening is a robustness test, not a correction of recorded measurements.

Annual terminal-digit distribution

year	measure	tenths digit	records	percent
2014	height	0	428,564	91.144
2014	height	1	3,555	0.756
2014	height	2	5,036	1.071
2014	height	3	4,013	0.853
2014	height	4	3,034	0.645
2014	height	5	16,040	3.411
2014	height	6	3,156	0.671
2014	height	7	2,678	0.570
2014	height	8	2,662	0.566
2014	height	9	1,466	0.312
2014	weight	0	398,022	84.649
2014	weight	1	6,356	1.352
2014	weight	2	7,603	1.617
2014	weight	3	6,456	1.373
2014	weight	4	6,514	1.385
2014	weight	5	22,464	4.778
2014	weight	6	6,721	1.429
2014	weight	7	5,522	1.174
2014	weight	8	6,013	1.279
2014	weight	9	4,533	0.964
2015	height	0	457,244	90.854
2015	height	1	3,889	0.773
2015	height	2	4,227	0.840
2015	height	3	3,610	0.717
2015	height	4	3,194	0.635
2015	height	5	19,644	3.903
2015	height	6	3,325	0.661
2015	height	7	2,931	0.582
2015	height	8	2,859	0.568
2015	height	9	2,351	0.467
2015	weight	0	403,146	80.105
2015	weight	1	9,107	1.810
2015	weight	2	9,778	1.943
2015	weight	3	9,323	1.852

year	measure	tenths digit	records	percent
2015	weight	4	8,894	1.767
2015	weight	5	28,946	5.752
2015	weight	6	9,233	1.835
2015	weight	7	8,606	1.710
2015	weight	8	8,711	1.731
2015	weight	9	7,530	1.496
2016	height	0	469,318	91.944
2016	height	1	3,538	0.693
2016	height	2	3,826	0.750
2016	height	3	3,005	0.589
2016	height	4	2,898	0.568
2016	height	5	17,504	3.429
2016	height	6	3,050	0.598
2016	height	7	2,553	0.500
2016	height	8	2,728	0.534
2016	height	9	2,019	0.396
2016	weight	0	411,688	80.654
2016	weight	1	9,435	1.848
2016	weight	2	9,449	1.851
2016	weight	3	8,795	1.723
2016	weight	4	8,752	1.715
2016	weight	5	28,634	5.610
2016	weight	6	8,989	1.761
2016	weight	7	8,257	1.618
2016	weight	8	8,654	1.695
2016	weight	9	7,786	1.525
2017	height	0	502,096	90.400
2017	height	1	4,602	0.829
2017	height	2	5,141	0.926
2017	height	3	4,127	0.743
2017	height	4	4,309	0.776
2017	height	5	18,880	3.399
2017	height	6	4,704	0.847
2017	height	7	3,936	0.709
2017	height	8	4,373	0.787
2017	height	9	3,248	0.585
2017	weight	0	439,118	79.061
2017	weight	1	10,799	1.944

year	measure	tenths digit	records	percent
2017	weight	2	11,317	2.038
2017	weight	3	10,909	1.964
2017	weight	4	10,332	1.860
2017	weight	5	31,396	5.653
2017	weight	6	11,048	1.989
2017	weight	7	10,395	1.872
2017	weight	8	10,666	1.920
2017	weight	9	9,436	1.699
2018	height	0	547,074	89.366
2018	height	1	5,266	0.860
2018	height	2	5,515	0.901
2018	height	3	4,904	0.801
2018	height	4	4,858	0.794
2018	height	5	25,974	4.243
2018	height	6	4,986	0.814
2018	height	7	4,768	0.779
2018	height	8	4,758	0.777
2018	height	9	4,070	0.665
2018	weight	0	469,918	76.762
2018	weight	1	13,189	2.154
2018	weight	2	13,757	2.247
2018	weight	3	13,220	2.160
2018	weight	4	12,777	2.087
2018	weight	5	38,154	6.233
2018	weight	6	13,634	2.227
2018	weight	7	12,618	2.061
2018	weight	8	13,059	2.133
2018	weight	9	11,847	1.935
2019	height	0	592,343	89.496
2019	height	1	5,405	0.817
2019	height	2	6,172	0.933
2019	height	3	5,478	0.828
2019	height	4	5,334	0.806
2019	height	5	27,913	4.217
2019	height	6	5,469	0.826
2019	height	7	4,660	0.704
2019	height	8	4,987	0.753
2019	height	9	4,104	0.620

year	measure	tenths digit	records	percent
2019	weight	0	511,603	77.297
2019	weight	1	13,961	2.109
2019	weight	2	14,423	2.179
2019	weight	3	14,184	2.143
2019	weight	4	13,807	2.086
2019	weight	5	39,472	5.964
2019	weight	6	14,392	2.174
2019	weight	7	13,595	2.054
2019	weight	8	13,922	2.103
2019	weight	9	12,506	1.890
2020	height	0	638,423	89.594
2020	height	1	6,034	0.847
2020	height	2	6,759	0.949
2020	height	3	5,732	0.804
2020	height	4	5,468	0.767
2020	height	5	30,186	4.236
2020	height	6	5,397	0.757
2020	height	7	5,074	0.712
2020	height	8	5,007	0.703
2020	height	9	4,493	0.631
2020	weight	0	534,156	74.962
2020	weight	1	17,341	2.434
2020	weight	2	18,278	2.565
2020	weight	3	17,618	2.472
2020	weight	4	17,126	2.403
2020	weight	5	41,303	5.796
2020	weight	6	17,741	2.490
2020	weight	7	16,624	2.333
2020	weight	8	16,722	2.347
2020	weight	9	15,664	2.198
2021	height	0	667,255	89.080
2021	height	1	5,351	0.714
2021	height	2	6,156	0.822
2021	height	3	5,467	0.730
2021	height	4	5,442	0.727
2021	height	5	39,382	5.258
2021	height	6	5,431	0.725
2021	height	7	5,090	0.680

year	measure	tenths digit	records	percent
2021	height	8	5,062	0.676
2021	height	9	4,415	0.589
2021	weight	0	557,204	74.388
2021	weight	1	18,520	2.472
2021	weight	2	19,512	2.605
2021	weight	3	19,069	2.546
2021	weight	4	18,352	2.450
2021	weight	5	43,486	5.805
2021	weight	6	19,303	2.577
2021	weight	7	18,153	2.423
2021	weight	8	18,556	2.477
2021	weight	9	16,896	2.256
2022	height	0	698,564	89.810
2022	height	1	6,514	0.837
2022	height	2	6,812	0.876
2022	height	3	6,214	0.799
2022	height	4	6,130	0.788
2022	height	5	30,629	3.938
2022	height	6	6,104	0.785
2022	height	7	5,682	0.730
2022	height	8	5,909	0.760
2022	height	9	5,268	0.677
2022	weight	0	597,048	76.759
2022	weight	1	17,193	2.210
2022	weight	2	18,436	2.370
2022	weight	3	17,607	2.264
2022	weight	4	17,149	2.205
2022	weight	5	41,903	5.387
2022	weight	6	18,247	2.346
2022	weight	7	17,242	2.217
2022	weight	8	17,428	2.241
2022	weight	9	15,573	2.002
2023	height	0	744,792	90.800
2023	height	1	5,466	0.666
2023	height	2	6,193	0.755
2023	height	3	5,628	0.686
2023	height	4	5,530	0.674
2023	height	5	32,940	4.016

year	measure	tenths digit	records	percent
2023	height	6	5,326	0.649
2023	height	7	4,975	0.607
2023	height	8	4,925	0.600
2023	height	9	4,477	0.546
2023	weight	0	647,635	78.956
2023	weight	1	16,503	2.012
2023	weight	2	17,857	2.177
2023	weight	3	17,153	2.091
2023	weight	4	16,737	2.040
2023	weight	5	39,097	4.766
2023	weight	6	17,375	2.118
2023	weight	7	16,172	1.972
2023	weight	8	16,763	2.044
2023	weight	9	14,960	1.824
2024	height	0	713,493	86.275
2024	height	1	8,040	0.972
2024	height	2	9,779	1.182
2024	height	3	8,989	1.087
2024	height	4	8,682	1.050
2024	height	5	47,880	5.790
2024	height	6	8,830	1.068
2024	height	7	7,472	0.904
2024	height	8	7,534	0.911
2024	height	9	6,299	0.762
2024	weight	0	588,490	71.160
2024	weight	1	23,711	2.867
2024	weight	2	25,855	3.126
2024	weight	3	25,569	3.092
2024	weight	4	24,596	2.974
2024	weight	5	47,234	5.712
2024	weight	6	24,569	2.971
2024	weight	7	22,891	2.768
2024	weight	8	23,312	2.819
2024	weight	9	20,771	2.512

Annual descriptive effects of precision coarsening

year	age restricted records	recorded high BMI pct	half unit coarsened high BMI pct	integer coarsened high BMI pct	difference pp	height integer pct	weight integer pct
2014	465,553	17.015	17.012	17.067	0.052	91.144	84.649
2015	502,190	18.051	18.052	18.129	0.078	90.854	80.105
2016	509,476	18.573	18.578	18.646	0.073	91.944	80.654
2017	554,667	19.620	19.621	19.706	0.085	90.400	79.061
2018	611,470	20.027	20.029	20.108	0.081	89.366	76.762
2019	661,480	20.186	20.185	20.256	0.070	89.496	77.297
2020	711,887	22.712	22.712	22.786	0.074	89.594	74.962
2021	748,731	22.604	22.607	22.666	0.062	89.080	74.388
2022	776,607	21.737	21.732	21.796	0.060	89.810	76.759
2023	819,624	21.144	21.143	21.191	0.047	90.800	78.956
2024	826,094	21.109	21.107	21.152	0.043	86.275	71.160

Adjusted high-BMI sensitivity (column block 1 of 2; first two columns repeated)

model	standard population	estimate 2014	estimate 2024	change percentage points	school cluster se	ci low	ci high	t value	df
half_unit_coarsened_all_schools	pooled_2014_2024	17.403	20.751	3.349	0.337	2.688	4.009	9.943	1,393
half_unit_coarsened_all_schools	fixed_2014	17.012	20.300	3.288	0.331	2.638	3.937	9.931	1,393
half_unit_coarsened_all_schools	equal_year	17.361	20.703	3.342	0.336	2.683	4.002	9.942	1,393
integer_coarsened_all_schools	pooled_2014_2024	17.457	20.801	3.344	0.335	2.687	4.002	9.975	1,393
integer_coarsened_all_schools	fixed_2014	17.067	20.351	3.284	0.330	2.637	3.931	9.963	1,393
integer_coarsened_all_schools	equal_year	17.415	20.753	3.338	0.335	2.681	3.994	9.974	1,393
integer_coarsened_fixed_704_schools	pooled_2014_2024	17.712	21.219	3.507	0.425	2.672	4.342	8.243	703
integer_coarsened_fixed_704_schools	fixed_2014	17.375	20.829	3.454	0.419	2.631	4.277	8.240	703
integer_coarsened_fixed_704_schools	equal_year	17.696	21.200	3.504	0.425	2.670	4.339	8.243	703
recorded_resolution_stable_p95_fixed_schools	pooled_2014_2024	17.669	20.669	3.000	0.524	1.969	4.031	5.723	377
recorded_resolution_stable_p95_fixed_schools	fixed_2014	17.373	20.332	2.959	0.517	1.942	3.977	5.719	377
recorded_resolution_stable_p95_fixed_schools	equal_year	17.655	20.652	2.998	0.524	1.968	4.028	5.723	377
recorded_resolution_stable_p90_fixed_schools	pooled_2014_2024	16.998	20.530	3.532	0.582	2.387	4.677	6.073	269
recorded_resolution_stable_p90_fixed_schools	fixed_2014	16.700	20.182	3.482	0.574	2.352	4.612	6.068	269
recorded_resolution_stable_p90_fixed_schools	equal_year	16.986	20.516	3.530	0.581	2.386	4.675	6.073	269

Adjusted high-BMI sensitivity (column block 2 of 2; first two columns repeated)

model	standard population	p value	records	school clusters
half_unit_coarsened_all_schools	pooled_2014_2024	<0.001	7,187,779	1,394
half_unit_coarsened_all_schools	fixed_2014	<0.001	7,187,779	1,394
half_unit_coarsened_all_schools	equal_year	<0.001	7,187,779	1,394
integer_coarsened_all_schools	pooled_2014_2024	<0.001	7,187,779	1,394

model	standard population	p value	records	school clusters
integer_coarsened_all_schools	fixed_2014	<0.001	7,187,779	1,394
integer_coarsened_all_schools	equal_year	<0.001	7,187,779	1,394
integer_coarsened_fixed_704_schools	pooled_2014_2024	<0.001	5,060,353	704
integer_coarsened_fixed_704_schools	fixed_2014	<0.001	5,060,353	704
integer_coarsened_fixed_704_schools	equal_year	<0.001	5,060,353	704
recorded_resolution_stable_p95_fixed_schools	pooled_2014_2024	<0.001	3,054,685	378
recorded_resolution_stable_p95_fixed_schools	fixed_2014	<0.001	3,054,685	378
recorded_resolution_stable_p95_fixed_schools	equal_year	<0.001	3,054,685	378
recorded_resolution_stable_p90_fixed_schools	pooled_2014_2024	<0.001	2,027,243	270
recorded_resolution_stable_p90_fixed_schools	fixed_2014	<0.001	2,027,243	270
recorded_resolution_stable_p90_fixed_schools	equal_year	<0.001	2,027,243	270

Adjusted 2019–2020 contrasts

scenario	early year	late year	estimate pp	school cluster se	ci low	ci high
half_unit_coarsened_all_schools	2019	2020	2.509	0.236	2.046	2.973
integer_coarsened_all_schools	2019	2020	2.512	0.236	2.049	2.976
integer_coarsened_fixed_704_schools	2019	2020	2.244	0.269	1.715	2.773
recorded_resolution_stable_p95_fixed_schools	2019	2020	2.058	0.332	1.406	2.711
recorded_resolution_stable_p90_fixed_schools	2019	2020	1.827	0.411	1.018	2.635

Conditional P50/P95 endpoints

model	quantile	conditional 2014 2024 change kg m2	school cluster se	records	school clusters	ci low	ci high
half_unit_coarsened_monthly_age_rcs	0.500	0.134	0.028	7,187,779	1,394	0.080	0.189
half_unit_coarsened_monthly_age_rcs	0.950	0.735	0.082	7,187,779	1,394	0.574	0.896
integer_coarsened_monthly_age_rcs	0.500	0.134	0.028	7,187,779	1,394	0.080	0.188
integer_coarsened_monthly_age_rcs	0.950	0.734	0.082	7,187,779	1,394	0.573	0.895
recorded_resolution_stable_p95_fixed_schools	0.500	0.133	0.045	3,054,685	378	0.045	0.220
recorded_resolution_stable_p95_fixed_schools	0.950	0.646	0.121	3,054,685	378	0.407	0.884
recorded_resolution_stable_p90_fixed_schools	0.500	0.178	0.052	2,027,243	270	0.076	0.281
recorded_resolution_stable_p90_fixed_schools	0.950	0.765	0.136	2,027,243	270	0.497	1.033

P95−P50 sensitivity (column block 1 of 2; first two columns repeated)

model	contrast	estimate difference kg m2	school cluster se	ci low	ci high	t value	df	p value	records
half_unit_coarsened_monthly_age_rcs	P95 minus P50	0.601	0.067	0.469	0.733	8.924	1,393	<0.001	7,187,779
integer_coarsened_monthly_age_rcs	P95 minus P50	0.599	0.068	0.467	0.732	8.873	1,393	<0.001	7,187,779
recorded_resolution_stable_p95_fixed_schools	P95 minus P50	0.513	0.096	0.325	0.701	5.355	377	<0.001	3,054,685
recorded_resolution_stable_p90_fixed_schools	P95 minus P50	0.587	0.106	0.377	0.796	5.523	269	<0.001	2,027,243

P95−P50 sensitivity (column block 2 of 2; first two columns repeated)

model	contrast	school clusters	multiplier bootstrap ci low	multiplier bootstrap ci high	multiplier bootstrap p value	bootstrap replicates	bootstrap seed
half_unit_coarsened_monthly_age_rcs	P95 minus P50	1,394	0.468	0.730	<0.001	19,999	20,260,711
integer_coarsened_monthly_age_rcs	P95 minus P50	1,394	0.468	0.729	<0.001	19,999	20,260,711
recorded_resolution_stable_p95_fixed_schools	P95 minus P50	378	0.328	0.697	<0.001	19,999	20,260,711
recorded_resolution_stable_p90_fixed_schools	P95 minus P50	270	0.379	0.794	<0.001	19,999	20,260,711

Quantile-model diagnostics (column block 1 of 2; first two columns repeated)

model	quantile	records	groups present	school clusters	parameters	iterations	converged	cycle	final difference
half_unit_coarsened_monthly_age_rcs	0.500	7,187,779	54,616	1,394	32	119	Yes	No	0.000
half_unit_coarsened_monthly_age_rcs	0.950	7,187,779	54,616	1,394	32	153	Yes	No	0.000
integer_coarsened_monthly_age_rcs	0.500	7,187,779	54,616	1,394	32	135	Yes	No	0.000
integer_coarsened_monthly_age_rcs	0.950	7,187,779	54,616	1,394	32	180	Yes	No	0.000
recorded_resolution_stable_p95_fixed_schools	0.500	3,054,685	45,722	378	32	137	Yes	No	0.000
recorded_resolution_stable_p95_fixed_schools	0.950	3,054,685	45,722	378	32	191	Yes	No	0.000
recorded_resolution_stable_p90_fixed_schools	0.500	2,027,243	41,998	270	32	134	Yes	No	0.000
recorded_resolution_stable_p90_fixed_schools	0.950	2,027,243	41,998	270	32	173	Yes	No	0.000

Quantile-model diagnostics (column block 2 of 2; first two columns repeated)

model	quantile	bandwidth	sparsity	score sum max abs	condition number	fit time seconds
half_unit_coarsened_monthly_age_rcs	0.500	0.065	5.962	117.500	1,287.7	70.061
half_unit_coarsened_monthly_age_rcs	0.950	0.062	50.130	14.050	1,287.7	96.179
integer_coarsened_monthly_age_rcs	0.500	0.065	5.970	51.500	1,287.7	77.377
integer_coarsened_monthly_age_rcs	0.950	0.062	50.336	10.050	1,287.7	95.844
recorded_resolution_stable_p95_fixed_schools	0.500	0.087	6.056	16.500	1,275.1	33.589
recorded_resolution_stable_p95_fixed_schools	0.950	0.082	50.387	12.750	1,275.1	43.385
recorded_resolution_stable_p90_fixed_schools	0.500	0.100	6.044	12.500	1,268.9	25.276
recorded_resolution_stable_p90_fixed_schools	0.950	0.094	50.937	13.850	1,268.9	27.675

Supplementary Table S4. Primary-model sensitivity and diagnostics, including joint exclusion of 2020–2021

Alternative standard populations, the fixed-school panel, joint year exclusion, and age-parameterization checks. In the analysis that removed 2020 and 2021 together while retaining the remaining model specification, the P95−P50 difference in 2014–2024 change was 0.612 kg/m² (95% CI 0.479–0.745; P<0.001; 5,727,161 records; 1,391 school codes), with a school-cluster multiplier-bootstrap interval of 0.483–0.744. Invalid full-age spline results were removed.

High-BMI standardization and fixed-panel analyses (column block 1 of 2; first two columns repeated)

model	standard population	estimate 2014	estimate 2024	change percentage points	school cluster se	ci low	ci high	t value	df
unadjusted_age_restricted	pooled_2014_2024	17.185	20.832	3.648	0.339	2.983	4.312	10.770	1,393
unadjusted_age_restricted	fixed_2014	17.015	20.634	3.620	0.337	2.959	4.280	10.756	1,393
unadjusted_age_restricted	equal_year	17.160	20.803	3.644	0.338	2.980	4.307	10.768	1,393
age_spline_age_restricted	fixed_2014	17.015	20.300	3.285	0.331	2.636	3.935	9.916	1,393
age_spline_age_restricted	equal_year	17.364	20.704	3.340	0.336	2.680	4.000	9.927	1,393
age_spline_fixed_schools	pooled_2014_2024	17.668	21.181	3.512	0.427	2.673	4.351	8.217	703
age_spline_fixed_schools	fixed_2014	17.328	20.786	3.458	0.421	2.632	4.285	8.214	703
age_spline_fixed_schools	equal_year	17.652	21.161	3.510	0.427	2.671	4.348	8.217	703

High-BMI standardization and fixed-panel analyses (column block 2 of 2; first two columns repeated)

model	standard population	p value	records	school clusters
unadjusted_age_restricted	pooled_2014_2024	<0.001	7,187,779	1,394
unadjusted_age_restricted	fixed_2014	<0.001	7,187,779	1,394
unadjusted_age_restricted	equal_year	<0.001	7,187,779	1,394
age_spline_age_restricted	fixed_2014	<0.001	7,187,779	1,394
age_spline_age_restricted	equal_year	<0.001	7,187,779	1,394
age_spline_fixed_schools	pooled_2014_2024	<0.001	5,060,353	704
age_spline_fixed_schools	fixed_2014	<0.001	5,060,353	704
age_spline_fixed_schools	equal_year	<0.001	5,060,353	704

Conditional upper-tail sensitivity (column block 1 of 2; first two columns repeated)

model	contrast	estimate difference kg m2	school cluster se	ci low	ci high	t value	df	p value	records
unadjusted_categorical_year	P95 minus P50	0.639	0.068	0.506	0.772	9.406	1,393	<0.001	7,187,779
monthly_age_rcs_conditional_qr_fixed_schools	P95 minus P50	0.599	0.083	0.437	0.761	7.253	703	<0.001	5,060,353
monthly_age_rcs_conditional_qr_excluding_2020_2021	P95 minus P50	0.612	0.068	0.479	0.745	9.051	1,390	<0.001	5,727,161

Conditional upper-tail sensitivity (column block 2 of 2; first two columns repeated)

model	contrast	school clusters	multiplier bootstrap ci low	multiplier bootstrap ci high	multiplier bootstrap p value	bootstrap replicates	bootstrap seed
unadjusted_categorical_year	P95 minus P50	1,394	0.507	0.769	<0.001	19,999	20,260,711
monthly_age_rcs_conditional_qr_fixed_schools	P95 minus P50	704	0.441	0.760	<0.001	19,999	20,260,711
monthly_age_rcs_conditional_qr_excluding_2020_2021	P95 minus P50	1,391	0.483	0.744	<0.001	19,999	20,260,711

Fully interacted overall-change sensitivity (column block 1 of 2; first two columns repeated)

model	age parameterization	age reference	estimate 2014	estimate 2024	estimate	school cluster se	ci low	ci high	t value
year_x_sex_x_grade_fully_interacted	centered_whitened_monthly_rcs	completed months as of 1 September, represented by month midpoint	17.426	20.696	3.270	0.338	2.607	3.933	9.668
year_x_sex_x_grade_fully_interacted	linear_monthly_age	completed months as of 1 September, represented by month midpoint	17.398	20.683	3.284	0.338	2.621	3.948	9.714

Fully interacted overall-change sensitivity (column block 2 of 2; first two columns repeated)

model	age parameterization	df	p value
year_x_sex_x_grade_fully_interacted	centered_whitened_monthly_rcs	1,393	<0.001
year_x_sex_x_grade_fully_interacted	linear_monthly_age	1,393	<0.001

Model diagnostics (column block 1 of 3; first two columns repeated)

model	age adjusted	age restricted	fixed schools	spline knots years	records	aggregate rows	patterns	school clusters	parameters
unadjusted_age_restricted	No	Yes	No		7,187,779	1,435,424	54,616	1,394	29
age_spline_age_restricted	Yes	Yes	No	6.291667, 7.958333, 9.708333, 11.625000	7,187,779	1,435,424	54,616	1,394	32
age_spline_fixed_schools	Yes	Yes	Yes	6.291667, 7.958333, 9.708333, 11.625000	5,060,353	1,063,107	51,590	704	32

Model diagnostics (column block 2 of 3; first two columns repeated)

model	age adjusted	iterations	converged	max score	condition number	source	name	n records	n clusters
unadjusted_age_restricted	No	5	Yes	0.000	404.712	high BMI			
age_spline_age_restricted	Yes	5	Yes	0.000	1,299.8	high BMI			
age_spline_fixed_schools	Yes	5	Yes	0.000	1,296.3	high BMI			
		5	Yes	0.000	1,854.3	subgroup	sex_interaction	7,187,779	1,394
		5	Yes	0.000	14,268.8	subgroup	grade_interaction	7,187,779	1,394
		5	Yes	0.000	75,729.6	subgroup	sex_grade_interaction	7,187,779	1,394
		5	Yes	0.000	72,580.8	subgroup	sex_grade_interaction_linear_month_age	7,187,779	1,394

Model diagnostics (column block 3 of 3; first two columns repeated)

model	age adjusted	n parameters
unadjusted_age_restricted	No	

model	age adjusted	n parameters
age_spline_age_restricted	Yes	
age_spline_fixed_schools	Yes	
		42
		82
		147
		145

Alternative linear age-in-months target contrasts (column block 1 of 2)

contrast	estimate difference (percentage points)	school-cluster SE	CI low	CI high	t value	df	P value	bootstrap CI low	bootstrap CI high
Boys minus girls	1.476	0.246	0.994	1.958	6.012	1,393	<0.001	0.998	1.954
Grade 6 minus Grade 5	0.665	0.434	−0.187	1.517	1.530	1,393	0.126	−0.171	1.527
Boys versus girls within Grades 4–6	2.638	0.326	1.999	3.277	8.101	1,393	<0.001	1.982	3.264

Alternative linear age-in-months target contrasts (column block 2 of 2)

contrast	bootstrap P value	bootstrap replicates	bootstrap seed	age parameterization	condition number
Boys minus girls	<0.001	9,999	20,260,812	linear age in months derived from age in days	72,580.8
Grade 6 minus Grade 5	0.127	9,999	20,260,813	linear age in months derived from age in days	72,580.8
Boys versus girls within Grades 4–6	<0.001	9,999	20,260,814	linear age in months derived from age in days	72,580.8

Supplementary Table S5. School-level adjusted high-BMI burden-pattern classification and stability

Exploratory school-level monitoring classifications; no school identifiers are shown. A grouped binomial logistic model of high-BMI events and records, aggregated by year × district × sex × grade with categorical main effects, supplied fitted stratum probabilities. For each school-year, expected events were the stratum-specific fitted probabilities multiplied by the corresponding school stratum counts and summed. The adjusted burden residual was 100 × (observed events/N − expected events/N), in percentage points. Annual P75 thresholds were calculated only among school-years with N≥50. Eligibility required at least 8 observed years, at least 2 observations in 2014–2016, and at least 2 in 2022–2024. Persistent high burden required high burden in ≥70% of observed years; emerging and declining high burden combined early/late counts with the sign of the N-weighted residual slope; stable low burden required ≤1 high-burden year overall and none in 2022–2024; all other eligible schools were fluctuating/intermittent. Categories describe contemporaneous relative burden and are neither forecasts, fixed school diagnoses, nor individual-child trajectories. The additional sensitivity analysis of age restriction and within-grade monthly-age adjustment is reported in Supplementary Methods SM4 and Tables SM3–SM4.

Baseline class counts

burden-pattern class	school count	percent of sample
Fluctuating/intermittent burden	313	38.930
Stable low burden	249	30.970
Emerging high burden	106	13.184
Declining high burden	93	11.567
Persistent high burden	43	5.348

One-factor stability analyses (column block 1 of 2; first two columns repeated)

scenario	burden-pattern-eligible schools	common eligible with baseline	agreement with baseline n	agreement with baseline pct	changed class n	cohen kappa	class n persistent high burden	class n emerging high burden	class n declining high burden
Baseline	804	804	804	100	0	1	43	106	93
High-burden P70	804	804	693	86.194	111	0.811	57	126	101
High-burden P80	804	804	647	80.473	157	0.724	23	96	67
Minimum 7 years	804	804	804	100	0	1	43	106	93
Minimum 9 years	804	804	804	100	0	1	43	106	93
Persistent share 60%	804	804	774	96.269	30	0.949	73	100	84
Persistent share 80%	804	804	781	97.139	23	0.960	20	115	98
Slope threshold 0.10	804	804	800	99.502	4	0.993	43	105	90
Slope threshold 0.20	804	804	791	98.383	13	0.977	43	103	83
Exclude 2020	804	804	773	96.144	31	0.947	51	105	91
Exclude 2020–2021	801	801	745	93.009	56	0.903	36	112	94

One-factor stability analyses (column block 2 of 2; first two columns repeated)

scenario	burden-pattern-eligible schools	class n fluctuating intermittent burden	class n stable low burden
Baseline	804	313	249
High-burden P70	804	324	196
High-burden P80	804	284	334
Minimum 7 years	804	313	249
Minimum 9 years	804	313	249
Persistent share 60%	804	298	249

scenario	burden-pattern-eligible schools	class n fluctuating intermittent burden	class n stable low burden
Persistent share 80%	804	322	249
Slope threshold 0.10	804	317	249
Slope threshold 0.20	804	326	249
Exclude 2020	804	291	266
Exclude 2020–2021	801	278	281

Supplementary Table S6. 2024 school PE-resource exploration

Cross-sectional exploratory associations. All four final resource exposures and model covariates were complete in the 951 matched schools. In the 996-school upstream resource report, average class size was undefined for 1 school (0.10%) because its class count was zero; that school was not in the matched model sample. Football-field count was blank for 4 schools (0.40%), and the unused current-year new-PE-teacher field for 12 schools (1.20%); the documented composite rule coded blank or non-numeric facility and partial-area components as zero. The 915-school binary model excluded 36 schools solely because their 2024 body-panel N was below 50. None of the eight tested resource-exposure associations met the 0.05 threshold after either BH-FDR (q=0.155–0.647) or Holm correction (adjusted P=0.155–1.000).

Sample flow

Stage	N	Note
Resource-report schools	996	Unique 2024 resource school codes
2024 body-panel schools after exclusion	963	Four schools excluded
Matched resource/body schools	951	2024 cross-sectional exploratory sample
Binary high-burden logistic sample	915	230 high-burden schools
QC-clean matched sample	706	No listed resource QC flag
QC-clean binary high-burden sample	704	Logistic sensitivity sample
Complete final model-variable table	951	No missing values in the four derived exposures, log school size, district, adjusted burden residual, or 2024 record count
Binary threshold exclusions	36	2024 body-panel N<50; threshold-based rather than missing-data deletion
Upstream undefined average class size	1/996 (0.10%)	Caused by a zero class count; school not present in the matched model sample
Upstream blank football-field count	4/996 (0.40%)	Archived facility composite deterministically coded blank/non-numeric components as zero
Upstream blank new-PE-teacher field	12/996 (1.20%)	Field not used in the four final PE-resource exposures

Primary and sensitivity models (column block 1 of 2; first two columns repeated)

scenario	model type	sample n	term	p value	estimate	ci low	ci high	odds ratio	events
WLS main	wls	951	z_students_per_class	0.019	1.442	0.233	2.651		
WLS main	wls	951	z_pe_per_1000	0.363	−0.618	−1.948	0.713		
WLS main	wls	951	z_log1p_area_per_student	0.097	−0.438	−0.955	0.080		
WLS main	wls	951	z_facility_count	0.192	−0.313	−0.784	0.157		
WLS main	wls	951	z_log_school_size	0.008	−1.442	−2.505	−0.379		
Logistic primary	logistic	915	z_students_per_class	0.045		1.008	2.240	1.503	230
Logistic primary	logistic	915	z_pe_per_1000	0.435		0.843	1.489	1.120	230
Logistic primary	logistic	915	z_log1p_area_per_student	0.647		0.882	1.223	1.039	230
Logistic primary	logistic	915	z_facility_count	0.062		0.686	1.009	0.832	230
Logistic primary	logistic	915	z_log_school_size	0.053		0.438	1.006	0.664	230
WLS QC-clean	wls	706	z_students_per_class	0.017	1.904	0.338	3.471		

scenario	model type	sample n	term	p value	estimate	ci low	ci high	odds ratio	events
WLS QC-clean	wls	706	z_pe_per_1000	0.044	−2.855	−5.638	−0.071		
WLS QC-clean	wls	706	z_log1p_area_per_student	0.052	−0.582	−1.171	0.006		
WLS QC-clean	wls	706	z_facility_count	0.429	−0.204	−0.708	0.301		
WLS QC-clean	wls	706	z_log_school_size	0.004	−1.825	−3.064	−0.586		
Logistic QC-clean	logistic	704	z_students_per_class	0.034		1.045	3.023	1.777	161
Logistic QC-clean	logistic	704	z_pe_per_1000	0.042		0.141	0.963	0.368	161
Logistic QC-clean	logistic	704	z_log1p_area_per_student	0.123		0.666	1.050	0.836	161
Logistic QC-clean	logistic	704	z_facility_count	0.489		0.729	1.163	0.921	161
Logistic QC-clean	logistic	704	z_log_school_size	0.005		0.289	0.804	0.482	161
WLS positive-area	wls	757	z_students_per_class	0.099	1.121	−0.211	2.452		
WLS positive-area	wls	757	z_pe_per_1000	0.287	−0.855	−2.430	0.720		
WLS positive-area	wls	757	z_log1p_area_per_student	0.110	−0.480	−1.069	0.109		
WLS positive-area	wls	757	z_facility_count	0.274	−0.279	−0.780	0.221		
WLS positive-area	wls	757	z_log_school_size	0.021	−1.442	−2.668	−0.216		
Logistic positive-area	logistic	757	z_students_per_class	0.160		0.883	2.119	1.368	192
Logistic positive-area	logistic	757	z_pe_per_1000	0.241		0.886	1.618	1.197	192
Logistic positive-area	logistic	757	z_log1p_area_per_student	0.667		0.789	1.164	0.958	192
Logistic positive-area	logistic	757	z_facility_count	0.146		0.690	1.057	0.854	192
Logistic positive-area	logistic	757	z_log_school_size	0.067		0.401	1.032	0.644	192
WLS without school size	wls	951	z_students_per_class	0.300	0.468	−0.417	1.354		
WLS without school size	wls	951	z_pe_per_1000	0.851	−0.125	−1.428	1.178		
WLS without school size	wls	951	z_log1p_area_per_student	0.285	−0.279	−0.790	0.232		
WLS without school size	wls	951	z_facility_count	0.018	−0.544	−0.994	−0.094		
Logistic without school size	logistic	915	z_students_per_class	0.379		0.864	1.467	1.126	230
Logistic without school size	logistic	915	z_pe_per_1000	0.283		0.883	1.534	1.163	230
Logistic without school size	logistic	915	z_log1p_area_per_student	0.433		0.907	1.255	1.067	230
Logistic without school size	logistic	915	z_facility_count	0.010		0.650	0.942	0.783	230

Primary and sensitivity models (column block 2 of 2; first two columns repeated)

scenario	model type	BH q	Holm p
WLS main	wls	0.155	0.155
WLS main	wls	0.484	1
WLS main	wls	0.194	0.486

scenario	model type	BH q	Holm p
WLS main	wls	0.307	0.768
WLS main	wls		
Logistic primary	logistic	0.166	0.318
Logistic primary	logistic	0.497	1
Logistic primary	logistic	0.647	1
Logistic primary	logistic	0.166	0.373
Logistic primary	logistic		
WLS QC-clean	wls		
WLS QC-clean	wls		
WLS QC-clean	wls		
WLS QC-clean	wls		
WLS QC-clean	wls		
Logistic QC-clean	logistic		
Logistic QC-clean	logistic		
Logistic QC-clean	logistic		
Logistic QC-clean	logistic		
Logistic QC-clean	logistic		
WLS positive-area	wls		
WLS positive-area	wls		
WLS positive-area	wls		
WLS positive-area	wls		
WLS positive-area	wls		
Logistic positive-area	logistic		
Logistic positive-area	logistic		
Logistic positive-area	logistic		
Logistic positive-area	logistic		
Logistic positive-area	logistic		
WLS without school size	wls		
WLS without school size	wls		
WLS without school size	wls		
WLS without school size	wls		
Logistic without school size	logistic		
Logistic without school size	logistic		

scenario	model type	BH q	Holm p
Logistic without school size	logistic		
Logistic without school size	logistic		

Supplementary Table S7. Annual observed nutritional status

Observed participating-school records after the four-school administrative exclusion; values are descriptive and not age-standardized.

Results

year	records	underweight n	underweight pct	overweight n	overweight pct	obesity n	obesity pct	high bmi n	high bmi pct
2014	470,204	37,879	8.056	48,412	10.296	31,654	6.732	80,066	17.028
2015	503,275	32,898	6.537	54,068	10.743	36,755	7.303	90,823	18.046
2016	510,439	34,017	6.664	56,586	11.086	38,210	7.486	94,796	18.571
2017	555,416	34,834	6.272	63,712	11.471	45,280	8.152	108,992	19.623
2018	612,173	38,595	6.305	70,695	11.548	51,902	8.478	122,597	20.027
2019	661,865	40,450	6.112	76,820	11.607	56,808	8.583	133,628	20.190
2020	712,573	40,008	5.615	91,801	12.883	70,041	9.829	161,842	22.712
2021	749,051	38,952	5.200	95,934	12.807	73,383	9.797	169,317	22.604
2022	777,826	45,961	5.909	96,680	12.430	72,359	9.303	169,039	21.732
2023	820,252	53,890	6.570	96,764	11.797	76,654	9.345	173,418	21.142
2024	826,998	59,637	7.211	97,297	11.765	77,256	9.342	174,553	21.107

Supplementary Table S8. Adjusted annual high-BMI prevalence and assessment of nonlinearity

Categorical year, a centered and whitened age-in-months spline, sex, grade and district; standardized to the pooled 2014–2024 covariate distribution with school-cluster inference.

Annual standardized prevalence

model	standard population	year	estimate percent	school cluster se	ci low	ci high	records	school clusters
age_spline_age_restricted	pooled_2014_2024	2014	17.406	0.244	16.927	17.886	7,187,779	1,394
age_spline_age_restricted	pooled_2014_2024	2015	18.386	0.197	18.000	18.772	7,187,779	1,394
age_spline_age_restricted	pooled_2014_2024	2016	18.818	0.214	18.398	19.238	7,187,779	1,394
age_spline_age_restricted	pooled_2014_2024	2017	19.807	0.224	19.368	20.246	7,187,779	1,394
age_spline_age_restricted	pooled_2014_2024	2018	20.184	0.231	19.732	20.637	7,187,779	1,394
age_spline_age_restricted	pooled_2014_2024	2019	20.245	0.221	19.811	20.680	7,187,779	1,394
age_spline_age_restricted	pooled_2014_2024	2020	22.754	0.231	22.301	23.208	7,187,779	1,394
age_spline_age_restricted	pooled_2014_2024	2021	22.405	0.234	21.946	22.864	7,187,779	1,394
age_spline_age_restricted	pooled_2014_2024	2022	21.512	0.210	21.100	21.925	7,187,779	1,394
age_spline_age_restricted	pooled_2014_2024	2023	20.899	0.205	20.497	21.300	7,187,779	1,394
age_spline_age_restricted	pooled_2014_2024	2024	20.752	0.223	20.315	21.190	7,187,779	1,394

Departure from a single linear log-odds trend

model	null hypothesis	df1	df2	wald f	p value	school clusters
age_spline_age_restricted	categorical year effects lie on one linear log-odds trend from 2014 to 2024	9	1,393	26.138	<0.001	1,394

Supplementary Table S9. Fixed-panel representation of retained records and school codes

Fixed-panel shares use age-valid retained records and school codes and describe representation within the analytic data, not population participation or reporting coverage. Primary fixed-panel analyses use 5,060,353 age-restricted records.

Results

year	all records age valid	fixed records age valid	fixed-panel record share (%)	all school codes	fixed schools	fixed-panel school-code share (%)	all high bmi pct	fixed high bmi pct
2014	470,204	384,487	81.770	1,092	704	64.469	17.028	17.346
2015	503,274	403,562	80.187	1,176	704	59.864	18.046	18.166
2016	510,439	415,826	81.464	1,137	704	61.917	18.571	18.673
2017	555,416	437,064	78.691	1,111	704	63.366	19.623	19.648
2018	612,173	460,116	75.161	1,023	704	68.817	20.027	20.086
2019	661,865	480,449	72.590	1,013	704	69.497	20.190	20.242
2020	712,573	493,894	69.311	1,011	704	69.634	22.712	22.472
2021	749,051	503,203	67.179	997	704	70.612	22.604	22.731
2022	777,826	500,666	64.367	998	704	70.541	21.732	21.723
2023	820,252	504,248	61.475	983	704	71.617	21.142	21.378
2024	826,998	485,898	58.754	963	704	73.105	21.107	21.533

Supplementary Table S10. Annual observed BMI percentiles

Empirical percentiles in the four-school-excluded primary sample aged 6.0–12.99 years; N=7,187,779 across 2014–2024.

Results

year	N	P50	P75	P90	P95	P95 minus P50	analytic sample
2014	465,553	16.392	18.395	21.208	23.139	6.746	primary_6.0_12.99_years
2015	502,190	16.479	18.611	21.429	23.438	6.958	primary_6.0_12.99_years
2016	509,476	16.517	18.647	21.482	23.469	6.953	primary_6.0_12.99_years
2017	554,667	16.568	18.808	21.720	23.725	7.157	primary_6.0_12.99_years
2018	611,470	16.562	18.848	21.809	23.808	7.246	primary_6.0_12.99_years
2019	661,480	16.562	18.861	21.879	23.951	7.389	primary_6.0_12.99_years
2020	711,887	16.760	19.204	22.222	24.305	7.544	primary_6.0_12.99_years
2021	748,731	16.826	19.230	22.313	24.342	7.516	primary_6.0_12.99_years
2022	776,607	16.741	19.204	22.222	24.295	7.553	primary_6.0_12.99_years
2023	819,624	16.644	19.150	22.230	24.395	7.751	primary_6.0_12.99_years
2024	826,094	16.599	19.150	22.299	24.402	7.804	primary_6.0_12.99_years

Supplementary Table S11. Conditional quantile results and internal validation

Covariate-adjusted changes in conditional BMI quantiles, with school-cluster inference and validation analyses; these estimates differ from changes in marginal population quantiles. The original P50/P95 validation and P95−P50 density sensitivity are retained below for continuity with the submitted results. Expanded validation at all four quantiles and full-sample covariance sensitivity are reported in Supplementary Methods SM3 and Tables SM1–SM2.

Conditional 2014–2024 quantile changes

model	quantile	conditional 2014 2024 change kg m2	school cluster se	records	school clusters	ci low	ci high
monthly_age_rcs_conditional_qr_main	0.500	0.134	0.028	7,187,779	1,394	0.079	0.188
monthly_age_rcs_conditional_qr_main	0.750	0.345	0.041	7,187,779	1,394	0.265	0.425
monthly_age_rcs_conditional_qr_main	0.900	0.620	0.062	7,187,779	1,394	0.498	0.742
monthly_age_rcs_conditional_qr_main	0.950	0.735	0.082	7,187,779	1,394	0.573	0.896

Formal cross-quantile contrasts (column block 1 of 2; first two columns repeated)

model	contrast	estimate difference kg m2	school cluster se	ci low	ci high	t value	df	p value	records
monthly_age_rcs_conditional_qr_main	P75 minus P50	0.211	0.021	0.171	0.252	10.226	1,393	<0.001	7,187,779
monthly_age_rcs_conditional_qr_main	P90 minus P50	0.486	0.045	0.397	0.575	10.736	1,393	<0.001	7,187,779
monthly_age_rcs_conditional_qr_main	P95 minus P50	0.601	0.068	0.468	0.734	8.867	1,393	<0.001	7,187,779

Formal cross-quantile contrasts (column block 2 of 2; first two columns repeated)

model	contrast	school clusters	multiplier bootstrap ci low	multiplier bootstrap ci high	multiplier bootstrap p value	bootstrap replicates	bootstrap seed
monthly_age_rcs_conditional_qr_main	P75 minus P50	1,394	0.171	0.251	<0.001	19,999	20,260,711
monthly_age_rcs_conditional_qr_main	P90 minus P50	1,394	0.398	0.573	<0.001	19,999	20,260,711
monthly_age_rcs_conditional_qr_main	P95 minus P50	1,394	0.469	0.731	<0.001	19,999	20,260,711

Original internal validation summary

Validation	Sample	Records	Strata / schools	Metric	Value	Upper value	Note
HiGHS exact LP	Balanced validation sample	51,480	1,716	P50 year−2024 coefficient absolute difference	0.002		
HiGHS exact LP	Balanced validation sample	51,480	1,716	P95 year−2024 coefficient absolute difference	0.000		
Local-density bread	Complete primary sample	7,187,779	1,394	P95−P50 SE range	0.065	0.065	Bandwidth 0.75-1.25
School pairs refit	Simple random validation sample	200,000	1,361	P95−P50 bootstrap SE	0.120		199 replicates

Original exact HiGHS validation details (column block 1 of 2; first two columns repeated)

quantile	validation sample n	validation design	random seed	custom converged	custom iterations	custom year 2024 coefficient	highs year 2024 coefficient	year coefficient absolute difference	maximum absolute coefficient difference
0.500	51,480	30 records per year x district x sex x grade stratum (1,716 strata)	20,260,711	Yes	1,726	0.137	0.139	0.002	0.002

quantile	validation sample n	validation design	random seed	custom converged	custom iterations	custom year 2024 coefficient	highs year 2024 coefficient	year coefficient absolute difference	maximum absolute coefficient difference
0.950	51,480	30 records per year x district x sex x grade stratum (1,716 strata)	20,260,711	Yes	869	0.809	0.809	0.000	0.000

Original exact HiGHS validation details (column block 2 of 2; first two columns repeated)

quantile	validation sample n	custom check loss	highs check loss	relative objective difference	highs status	highs message
0.500	51,480	59,001.1	59,001.1	0.000	0	Optimization terminated successfully. (HiGHS Status 7: Optimal)
0.950	51,480	22,458.8	22,458.8	0.000	0	Optimization terminated successfully. (HiGHS Status 7: Optimal)

Original local-density bread sensitivity

bread	bandwidth scale	p95 minus p50 estimate	school cluster se	ci low	ci high	df	p value	records	school clusters
observation-kernel-weighted conditional-density bread	0.750	0.601	0.065	0.473	0.729	1,393	<0.001	7,187,779	1,394
observation-kernel-weighted conditional-density bread	1	0.601	0.065	0.474	0.729	1,393	<0.001	7,187,779	1,394
observation-kernel-weighted conditional-density bread	1.250	0.601	0.065	0.474	0.728	1,393	<0.001	7,187,779	1,394

Supplementary Table S12. Adjusted high-BMI prevalence changes for all sex, grade, and sex-by-grade groups

Repeated cross-sectional subgroup changes from the categorical-year models using the uniform centered and whitened age-in-months spline and school-cluster inference. All 12 sex-by-grade estimates are reported, with changes expressed in percentage points.

Results (column block 1 of 2; first two columns repeated)

group type	group	rate 2014	rate 2024	estimate	school cluster se	ci low	ci high	t value	df
Sex	Boys	18.020	22.129	4.109	0.361	3.401	4.817	11.390	1,393
Sex	Girls	16.759	19.216	2.456	0.357	1.756	3.157	6.883	1,393
Grade	Grade 1	16.637	15.964	−0.673	0.595	−1.841	0.495	−1.130	1,393
Grade	Grade 2	17.309	18.458	1.148	0.547	0.075	2.222	2.099	1,393
Grade	Grade 3	17.306	20.345	3.040	0.446	2.165	3.914	6.816	1,393
Grade	Grade 4	18.662	23.148	4.486	0.452	3.599	5.372	9.927	1,393
Grade	Grade 5	16.257	22.356	6.099	0.463	5.191	7.006	13.183	1,393
Grade	Grade 6	18.446	25.338	6.892	0.499	5.913	7.871	13.816	1,393
Sex x grade	Boys Grade 1	15.784	15.070	−0.714	0.634	−1.958	0.530	−1.126	1,393
Sex x grade	Boys Grade 2	17.726	19.259	1.533	0.559	0.436	2.630	2.742	1,393
Sex x grade	Boys Grade 3	18.184	21.451	3.266	0.501	2.284	4.248	6.522	1,393
Sex x grade	Boys Grade 4	19.756	25.570	5.813	0.493	4.846	6.781	11.791	1,393
Sex x grade	Boys Grade 5	17.509	24.874	7.365	0.527	6.331	8.398	13.978	1,393
Sex x grade	Boys Grade 6	19.671	27.674	8.004	0.574	6.877	9.130	13.934	1,393
Sex x grade	Girls Grade 1	17.739	16.904	−0.836	0.625	−2.062	0.391	−1.337	1,393
Sex x grade	Girls Grade 2	16.901	17.546	0.645	0.605	−0.543	1.832	1.065	1,393
Sex x grade	Girls Grade 3	16.347	19.104	2.758	0.498	1.781	3.734	5.542	1,393
Sex x grade	Girls Grade 4	17.436	20.464	3.028	0.537	1.974	4.083	5.635	1,393
Sex x grade	Girls Grade 5	14.828	19.554	4.726	0.513	3.719	5.733	9.204	1,393
Sex x grade	Girls Grade 6	17.051	22.732	5.681	0.531	4.641	6.722	10.708	1,393

Results (column block 2 of 2; first two columns repeated)

group type	group	p value
Sex	Boys	<0.001
Sex	Girls	<0.001
Grade	Grade 1	0.259
Grade	Grade 2	0.036
Grade	Grade 3	<0.001
Grade	Grade 4	<0.001
Grade	Grade 5	<0.001
Grade	Grade 6	<0.001
Sex x grade	Boys Grade 1	0.260

group type	group	p value
Sex x grade	Boys Grade 2	0.006
Sex x grade	Boys Grade 3	<0.001
Sex x grade	Boys Grade 4	<0.001
Sex x grade	Boys Grade 5	<0.001
Sex x grade	Boys Grade 6	<0.001
Sex x grade	Girls Grade 1	0.182
Sex x grade	Girls Grade 2	0.287
Sex x grade	Girls Grade 3	<0.001
Sex x grade	Girls Grade 4	<0.001
Sex x grade	Girls Grade 5	<0.001
Sex x grade	Girls Grade 6	<0.001

Supplementary Table S13. Interaction tests and targeted prevalence-change contrasts

School-cluster joint interaction tests and targeted contrasts in changes in adjusted high-BMI prevalence. The year × sex × grade test supports heterogeneity across the 12 groups; the Grade 6−Grade 5 contrast has a 95% CI crossing zero.

Joint interaction tests

test	df1	df2	wald f	p value
Year x sex interaction	10	1,393	10.013	<0.001
Year x grade interaction	50	1,393	5.505	<0.001
Year x sex x grade interaction	50	1,393	2.258	<0.001

Targeted formal contrasts (column block 1 of 2; first two columns repeated)

contrast	estimate difference pp	school cluster se	ci low	ci high	t value	df	p value	bootstrap ci low	bootstrap ci high
Boys minus girls	1.652	0.247	1.169	2.136	6.699	1,393	<0.001	1.169	2.139
Grade 6 minus Grade 5	0.793	0.435	−0.061	1.647	1.822	1,393	0.069	−0.060	1.659
Boys versus girls within Grades 4–6	2.591	0.325	1.953	3.229	7.964	1,393	<0.001	1.960	3.229

Targeted formal contrasts (column block 2 of 2; first two columns repeated)

contrast	estimate difference pp	bootstrap p value	bootstrap replicates	bootstrap seed
Boys minus girls	1.652	<0.001	9,999	20,260,712
Grade 6 minus Grade 5	0.793	0.071	9,999	20,260,713
Boys versus girls within Grades 4–6	2.591	<0.001	9,999	20,260,714

Supplementary Table S14. District-year standardized high-BMI prevalence

Secondary sex-by-grade direct standardization in the primary 6.0–12.99-year sample; this analysis is not adjusted for continuous age in months. Pointwise school-cluster bootstrap intervals for endpoint prevalence and changes are reported in Supplementary Methods SM5 and Table SM5; intervals for all 143 district-year estimates are included in the source-data workbook.

Results

district en	year	standardized high BMI pct	observed N	analytic sample	standardization
Lintong	2014	16.720	33,441	primary_6.0_12.99_years	pooled primary-sample sex-by-grade distribution
Zhouzhi	2014	10.753	30,681	primary_6.0_12.99_years	pooled primary-sample sex-by-grade distribution
Xincheng	2014	19.187	32,549	primary_6.0_12.99_years	pooled primary-sample sex-by-grade distribution
Weiyang	2014	17.829	52,741	primary_6.0_12.99_years	pooled primary-sample sex-by-grade distribution
Baqiao	2014	16.159	35,899	primary_6.0_12.99_years	pooled primary-sample sex-by-grade distribution
Beilin	2014	19.300	34,768	primary_6.0_12.99_years	pooled primary-sample sex-by-grade distribution
Lianhu	2014	19.062	42,541	primary_6.0_12.99_years	pooled primary-sample sex-by-grade distribution
Lantian	2014	12.946	26,831	primary_6.0_12.99_years	pooled primary-sample sex-by-grade distribution
Huyi	2014	13.545	27,180	primary_6.0_12.99_years	pooled primary-sample sex-by-grade distribution
Chang'an	2014	17.401	54,644	primary_6.0_12.99_years	pooled primary-sample sex-by-grade distribution
Yanliang	2014	20.254	13,398	primary_6.0_12.99_years	pooled primary-sample sex-by-grade distribution
Yanta	2014	18.489	67,219	primary_6.0_12.99_years	pooled primary-sample sex-by-grade distribution
Gaoling	2014	18.185	13,661	primary_6.0_12.99_years	pooled primary-sample sex-by-grade distribution
Lintong	2015	17.187	35,389	primary_6.0_12.99_years	pooled primary-sample sex-by-grade distribution
Zhouzhi	2015	10.967	31,871	primary_6.0_12.99_years	pooled primary-sample sex-by-grade distribution
Xincheng	2015	20.586	32,400	primary_6.0_12.99_years	pooled primary-sample sex-by-grade distribution
Weiyang	2015	18.078	61,642	primary_6.0_12.99_years	pooled primary-sample sex-by-grade distribution
Baqiao	2015	19.536	41,145	primary_6.0_12.99_years	pooled primary-sample sex-by-grade distribution
Beilin	2015	20.487	36,288	primary_6.0_12.99_years	pooled primary-sample sex-by-grade distribution
Lianhu	2015	20.005	45,223	primary_6.0_12.99_years	pooled primary-sample sex-by-grade distribution
Lantian	2015	15.489	26,860	primary_6.0_12.99_years	pooled primary-sample sex-by-grade distribution
Huyi	2015	14.267	28,517	primary_6.0_12.99_years	pooled primary-sample sex-by-grade distribution
Chang'an	2015	18.223	60,553	primary_6.0_12.99_years	pooled primary-sample sex-by-grade distribution
Yanliang	2015	17.098	14,111	primary_6.0_12.99_years	pooled primary-sample sex-by-grade distribution
Yanta	2015	19.075	71,451	primary_6.0_12.99_years	pooled primary-sample sex-by-grade distribution
Gaoling	2015	20.547	16,740	primary_6.0_12.99_years	pooled primary-sample sex-by-grade distribution
Lintong	2016	18.361	32,500	primary_6.0_12.99_years	pooled primary-sample sex-by-grade distribution
Zhouzhi	2016	13.166	31,693	primary_6.0_12.99_years	pooled primary-sample sex-by-grade distribution
Xincheng	2016	20.966	32,204	primary_6.0_12.99_years	pooled primary-sample sex-by-grade distribution
Weiyang	2016	20.245	61,723	primary_6.0_12.99_years	pooled primary-sample sex-by-grade distribution
Baqiao	2016	19.642	44,740	primary_6.0_12.99_years	pooled primary-sample sex-by-grade distribution
Beilin	2016	20.079	37,302	primary_6.0_12.99_years	pooled primary-sample sex-by-grade distribution

district en	year	standardized high BMI pct	observed N	analytic sample	standardization
Lianhu	2016	19.175	46,226	primary_6.0_12.99_years	pooled primary-sample sex-by-grade distribution
Lantian	2016	15.457	25,564	primary_6.0_12.99_years	pooled primary-sample sex-by-grade distribution
Huyi	2016	14.385	24,873	primary_6.0_12.99_years	pooled primary-sample sex-by-grade distribution
Chang'an	2016	18.287	59,505	primary_6.0_12.99_years	pooled primary-sample sex-by-grade distribution
Yanliang	2016	18.686	14,622	primary_6.0_12.99_years	pooled primary-sample sex-by-grade distribution
Yanta	2016	19.267	80,932	primary_6.0_12.99_years	pooled primary-sample sex-by-grade distribution
Gaoling	2016	19.349	17,592	primary_6.0_12.99_years	pooled primary-sample sex-by-grade distribution
Lintong	2017	20.593	35,809	primary_6.0_12.99_years	pooled primary-sample sex-by-grade distribution
Zhouzhi	2017	11.940	33,123	primary_6.0_12.99_years	pooled primary-sample sex-by-grade distribution
Xincheng	2017	20.705	31,970	primary_6.0_12.99_years	pooled primary-sample sex-by-grade distribution
Weiyang	2017	21.126	81,305	primary_6.0_12.99_years	pooled primary-sample sex-by-grade distribution
Baqiao	2017	21.627	45,446	primary_6.0_12.99_years	pooled primary-sample sex-by-grade distribution
Beilin	2017	20.950	38,564	primary_6.0_12.99_years	pooled primary-sample sex-by-grade distribution
Lianhu	2017	20.194	45,232	primary_6.0_12.99_years	pooled primary-sample sex-by-grade distribution
Lantian	2017	17.824	25,753	primary_6.0_12.99_years	pooled primary-sample sex-by-grade distribution
Huyi	2017	15.468	29,911	primary_6.0_12.99_years	pooled primary-sample sex-by-grade distribution
Chang'an	2017	20.119	66,998	primary_6.0_12.99_years	pooled primary-sample sex-by-grade distribution
Yanliang	2017	19.537	15,418	primary_6.0_12.99_years	pooled primary-sample sex-by-grade distribution
Yanta	2017	19.686	87,231	primary_6.0_12.99_years	pooled primary-sample sex-by-grade distribution
Gaoling	2017	22.032	17,907	primary_6.0_12.99_years	pooled primary-sample sex-by-grade distribution
Lintong	2018	18.510	36,391	primary_6.0_12.99_years	pooled primary-sample sex-by-grade distribution
Zhouzhi	2018	12.257	33,582	primary_6.0_12.99_years	pooled primary-sample sex-by-grade distribution
Xincheng	2018	20.826	32,599	primary_6.0_12.99_years	pooled primary-sample sex-by-grade distribution
Weiyang	2018	21.960	97,447	primary_6.0_12.99_years	pooled primary-sample sex-by-grade distribution
Baqiao	2018	22.411	51,704	primary_6.0_12.99_years	pooled primary-sample sex-by-grade distribution
Beilin	2018	22.860	40,217	primary_6.0_12.99_years	pooled primary-sample sex-by-grade distribution
Lianhu	2018	20.206	49,698	primary_6.0_12.99_years	pooled primary-sample sex-by-grade distribution
Lantian	2018	18.812	25,082	primary_6.0_12.99_years	pooled primary-sample sex-by-grade distribution
Huyi	2018	17.100	30,808	primary_6.0_12.99_years	pooled primary-sample sex-by-grade distribution
Chang'an	2018	20.219	75,379	primary_6.0_12.99_years	pooled primary-sample sex-by-grade distribution
Yanliang	2018	19.517	16,389	primary_6.0_12.99_years	pooled primary-sample sex-by-grade distribution
Yanta	2018	20.110	99,849	primary_6.0_12.99_years	pooled primary-sample sex-by-grade distribution
Gaoling	2018	21.411	22,325	primary_6.0_12.99_years	pooled primary-sample sex-by-grade distribution
Lintong	2019	20.992	37,700	primary_6.0_12.99_years	pooled primary-sample sex-by-grade distribution
Zhouzhi	2019	14.033	34,668	primary_6.0_12.99_years	pooled primary-sample sex-by-grade distribution
Xincheng	2019	20.615	33,137	primary_6.0_12.99_years	pooled primary-sample sex-by-grade distribution
Weiyang	2019	21.099	113,356	primary_6.0_12.99_years	pooled primary-sample sex-by-grade distribution
Baqiao	2019	21.696	57,381	primary_6.0_12.99_years	pooled primary-sample sex-by-grade distribution

district en	year	standardized high BMI pct	observed N	analytic sample	standardization
Beilin	2019	23.119	42,271	primary_6.0_12.99_years	pooled primary-sample sex-by-grade distribution
Lianhu	2019	21.232	51,960	primary_6.0_12.99_years	pooled primary-sample sex-by-grade distribution
Lantian	2019	18.882	24,420	primary_6.0_12.99_years	pooled primary-sample sex-by-grade distribution
Huyi	2019	16.123	31,803	primary_6.0_12.99_years	pooled primary-sample sex-by-grade distribution
Chang'an	2019	20.452	86,849	primary_6.0_12.99_years	pooled primary-sample sex-by-grade distribution
Yanliang	2019	19.330	17,229	primary_6.0_12.99_years	pooled primary-sample sex-by-grade distribution
Yanta	2019	20.142	105,800	primary_6.0_12.99_years	pooled primary-sample sex-by-grade distribution
Gaoling	2019	22.456	24,906	primary_6.0_12.99_years	pooled primary-sample sex-by-grade distribution
Lintong	2020	21.996	38,653	primary_6.0_12.99_years	pooled primary-sample sex-by-grade distribution
Zhouzhi	2020	15.511	35,959	primary_6.0_12.99_years	pooled primary-sample sex-by-grade distribution
Xincheng	2020	26.380	34,201	primary_6.0_12.99_years	pooled primary-sample sex-by-grade distribution
Weiyang	2020	24.875	127,204	primary_6.0_12.99_years	pooled primary-sample sex-by-grade distribution
Baqiao	2020	24.298	63,252	primary_6.0_12.99_years	pooled primary-sample sex-by-grade distribution
Beilin	2020	23.541	43,618	primary_6.0_12.99_years	pooled primary-sample sex-by-grade distribution
Lianhu	2020	22.177	53,432	primary_6.0_12.99_years	pooled primary-sample sex-by-grade distribution
Lantian	2020	21.090	24,389	primary_6.0_12.99_years	pooled primary-sample sex-by-grade distribution
Huyi	2020	18.707	32,731	primary_6.0_12.99_years	pooled primary-sample sex-by-grade distribution
Chang'an	2020	24.209	96,317	primary_6.0_12.99_years	pooled primary-sample sex-by-grade distribution
Yanliang	2020	19.321	18,082	primary_6.0_12.99_years	pooled primary-sample sex-by-grade distribution
Yanta	2020	22.040	117,844	primary_6.0_12.99_years	pooled primary-sample sex-by-grade distribution
Gaoling	2020	25.587	26,205	primary_6.0_12.99_years	pooled primary-sample sex-by-grade distribution
Lintong	2021	23.002	39,976	primary_6.0_12.99_years	pooled primary-sample sex-by-grade distribution
Zhouzhi	2021	16.522	34,863	primary_6.0_12.99_years	pooled primary-sample sex-by-grade distribution
Xincheng	2021	23.859	35,960	primary_6.0_12.99_years	pooled primary-sample sex-by-grade distribution
Weiyang	2021	25.445	138,570	primary_6.0_12.99_years	pooled primary-sample sex-by-grade distribution
Baqiao	2021	23.715	68,168	primary_6.0_12.99_years	pooled primary-sample sex-by-grade distribution
Beilin	2021	24.244	45,393	primary_6.0_12.99_years	pooled primary-sample sex-by-grade distribution
Lianhu	2021	23.546	55,594	primary_6.0_12.99_years	pooled primary-sample sex-by-grade distribution
Lantian	2021	21.234	23,155	primary_6.0_12.99_years	pooled primary-sample sex-by-grade distribution
Huyi	2021	19.530	33,101	primary_6.0_12.99_years	pooled primary-sample sex-by-grade distribution
Chang'an	2021	22.375	102,610	primary_6.0_12.99_years	pooled primary-sample sex-by-grade distribution
Yanliang	2021	19.500	16,791	primary_6.0_12.99_years	pooled primary-sample sex-by-grade distribution
Yanta	2021	21.016	127,613	primary_6.0_12.99_years	pooled primary-sample sex-by-grade distribution
Gaoling	2021	22.508	26,937	primary_6.0_12.99_years	pooled primary-sample sex-by-grade distribution
Lintong	2022	22.987	38,102	primary_6.0_12.99_years	pooled primary-sample sex-by-grade distribution
Zhouzhi	2022	15.882	34,405	primary_6.0_12.99_years	pooled primary-sample sex-by-grade distribution
Xincheng	2022	23.896	36,726	primary_6.0_12.99_years	pooled primary-sample sex-by-grade distribution
Weiyang	2022	23.583	142,839	primary_6.0_12.99_years	pooled primary-sample sex-by-grade distribution

district en	year	standardized high BMI pct	observed N	analytic sample	standardization
Baqiao	2022	21.420	73,985	primary_6.0_12.99_years	pooled primary-sample sex-by-grade distribution
Beilin	2022	23.400	46,719	primary_6.0_12.99_years	pooled primary-sample sex-by-grade distribution
Lianhu	2022	21.408	57,596	primary_6.0_12.99_years	pooled primary-sample sex-by-grade distribution
Lantian	2022	18.448	22,051	primary_6.0_12.99_years	pooled primary-sample sex-by-grade distribution
Huyi	2022	20.089	33,455	primary_6.0_12.99_years	pooled primary-sample sex-by-grade distribution
Chang'an	2022	21.291	109,690	primary_6.0_12.99_years	pooled primary-sample sex-by-grade distribution
Yanliang	2022	19.534	18,340	primary_6.0_12.99_years	pooled primary-sample sex-by-grade distribution
Yanta	2022	20.985	134,581	primary_6.0_12.99_years	pooled primary-sample sex-by-grade distribution
Gaoling	2022	23.462	28,118	primary_6.0_12.99_years	pooled primary-sample sex-by-grade distribution
Lintong	2023	21.620	37,163	primary_6.0_12.99_years	pooled primary-sample sex-by-grade distribution
Zhouzhi	2023	17.395	33,978	primary_6.0_12.99_years	pooled primary-sample sex-by-grade distribution
Xincheng	2023	23.188	37,298	primary_6.0_12.99_years	pooled primary-sample sex-by-grade distribution
Weiyang	2023	23.414	153,703	primary_6.0_12.99_years	pooled primary-sample sex-by-grade distribution
Baqiao	2023	20.998	78,649	primary_6.0_12.99_years	pooled primary-sample sex-by-grade distribution
Beilin	2023	20.803	49,737	primary_6.0_12.99_years	pooled primary-sample sex-by-grade distribution
Lianhu	2023	21.545	59,198	primary_6.0_12.99_years	pooled primary-sample sex-by-grade distribution
Lantian	2023	21.168	20,348	primary_6.0_12.99_years	pooled primary-sample sex-by-grade distribution
Huyi	2023	19.122	33,512	primary_6.0_12.99_years	pooled primary-sample sex-by-grade distribution
Chang'an	2023	20.679	119,383	primary_6.0_12.99_years	pooled primary-sample sex-by-grade distribution
Yanliang	2023	19.308	19,481	primary_6.0_12.99_years	pooled primary-sample sex-by-grade distribution
Yanta	2023	19.238	148,461	primary_6.0_12.99_years	pooled primary-sample sex-by-grade distribution
Gaoling	2023	24.117	28,713	primary_6.0_12.99_years	pooled primary-sample sex-by-grade distribution
Lintong	2024	22.573	34,908	primary_6.0_12.99_years	pooled primary-sample sex-by-grade distribution
Zhouzhi	2024	17.046	30,772	primary_6.0_12.99_years	pooled primary-sample sex-by-grade distribution
Xincheng	2024	22.063	36,765	primary_6.0_12.99_years	pooled primary-sample sex-by-grade distribution
Weiyang	2024	23.295	152,922	primary_6.0_12.99_years	pooled primary-sample sex-by-grade distribution
Baqiao	2024	21.429	88,267	primary_6.0_12.99_years	pooled primary-sample sex-by-grade distribution
Beilin	2024	21.651	50,133	primary_6.0_12.99_years	pooled primary-sample sex-by-grade distribution
Lianhu	2024	18.816	58,726	primary_6.0_12.99_years	pooled primary-sample sex-by-grade distribution
Lantian	2024	19.277	18,486	primary_6.0_12.99_years	pooled primary-sample sex-by-grade distribution
Huyi	2024	19.080	32,335	primary_6.0_12.99_years	pooled primary-sample sex-by-grade distribution
Chang'an	2024	21.355	122,607	primary_6.0_12.99_years	pooled primary-sample sex-by-grade distribution
Yanliang	2024	21.315	19,120	primary_6.0_12.99_years	pooled primary-sample sex-by-grade distribution
Yanta	2024	19.113	152,898	primary_6.0_12.99_years	pooled primary-sample sex-by-grade distribution
Gaoling	2024	22.627	28,155	primary_6.0_12.99_years	pooled primary-sample sex-by-grade distribution

Supplementary Table S15. Exploratory changes in lower conditional BMI quantiles, 2014–2024.

Quantile	Change (kg/m²)	Pooled-density 95% CI	P value	Kernel-weighted 95% CI
P5	0.097	0.027 to 0.167	0.007	0.022 to 0.172
P10	0.047	−0.008 to 0.103	0.095	−0.009 to 0.103
P25	0.048	−0.002 to 0.097	0.059	−0.002 to 0.098

Note. Exploratory lower-quantile models were added during peer review and included the same 7,187,779 records and 1,394 school codes as the primary analysis. Estimates are covariate-adjusted 2024-versus-2014 changes in conditional BMI quantiles from models including categorical year, the age-in-months spline, sex, grade, and district. Both covariance methods used the same fitted coefficients and school-level scores. Kernel-weighted intervals use observation-level Epanechnikov weighting at the default bandwidth. Confidence intervals are pointwise 95% school-cluster intervals with 1,393 t degrees of freedom; P values are unadjusted and use the pooled-density covariance. These exploratory estimates supplement the primary quantile analyses. BMI, body mass index; CI, confidence interval. Supplementary Methods SM8 describes the validation and bandwidth sensitivity.

Supplementary Table S16. WHO classification sensitivity: endpoint changes and targeted contrasts.

Panel A. Adjusted high-BMI prevalence and 2014–2024 change

Classification / scenario	Records	2014 (%)	2024 (%)	Change, pp (95% CI)
National: primary sample	7,187,779	17.41	20.75	3.346 (2.685 to 4.007)
WHO: September 1	7,187,779	27.36	30.59	3.226 (2.473 to 3.979)
WHO: October 15	7,187,779	26.70	29.97	3.268 (2.521 to 4.016)
WHO: November 15	7,187,779	26.25	29.54	3.290 (2.545 to 4.036)
WHO: flags excluded	7,155,185	27.26	30.43	3.166 (2.426 to 3.906)
National: same flag-excluded sample	7,155,185	17.23	20.54	3.311 (2.668 to 3.954)

Panel B. WHO September 1 targeted differences in change

Contrast	Difference, pp	95% CI	P value
Boys − girls	1.376	0.785 to 1.967	<0.001
Boys − girls within Grades 4–6	2.898	2.111 to 3.686	<0.001
Grade 6 − Grade 5	0.439	−0.581 to 1.458	0.399

Note. High BMI is defined by the national sex–grade cut-points or WHO BMI-for-age Z score >+1, as labeled. Models retain the original covariates, school clustering, and pooled primary-sample standardization weights. All analyses include 1,394 school codes. October 15 and November 15 change classification age only (+44 and +75 days); primary age eligibility and the model age spline remain fixed. Flag-excluded rows omit WHO Z scores <−5 or >+5 at September 1. Panel B uses the original interaction models and subgroup reference weights in the full primary sample. Intervals are pointwise 95% school-cluster t intervals. The Grades 4–6 contrast compares boys with girls within these grades. pp, percentage points; CI, confidence interval. See SM9.

Supplementary Table S17. Annual high-BMI prevalence under national and WHO classifications.

Panel A. Observed prevalence (%) in the unchanged primary sample

Year	Records	National	WHO Sep 1	WHO Oct 15	WHO Nov 15
2014	465,553	17.01	27.33	26.66	26.22
2015	502,190	18.05	28.64	27.98	27.51
2016	509,476	18.57	29.00	28.33	27.87
2017	554,667	19.62	30.18	29.51	29.04
2018	611,470	20.03	30.33	29.69	29.23
2019	661,480	20.19	30.26	29.61	29.13
2020	711,887	22.71	33.37	32.68	32.19
2021	748,731	22.60	33.41	32.67	32.17
2022	776,607	21.74	32.40	31.69	31.22
2023	819,624	21.14	31.30	30.65	30.19
2024	826,094	21.11	30.90	30.27	29.83

Note. All columns use the same 7,187,779 primary-analysis records. National classification applies the sex–grade cut-points to BMI rounded to 0.1 kg/m². WHO classification applies Z score >+1 to unrounded BMI, with age evaluated at the indicated reference date. The alternative dates are classification-age scenarios, not individual measurement dates. These rows retain WHO extreme-Z-score flags; flag counts, obesity counts, and classification-agreement counts are provided in the code archive.

Supplementary Table S17 (continued). Panel B. Adjusted annual prevalence (%)

Year	National	WHO Sep 1 (95% CI)	WHO Oct 15	WHO Nov 15
2014	17.41	27.36 (26.81–27.92)	26.70	26.25
2015	18.39	28.64 (28.18–29.10)	27.98	27.51
2016	18.82	29.07 (28.55–29.58)	28.40	27.94
2017	19.81	30.28 (29.72–30.83)	29.60	29.14
2018	20.18	30.57 (30.04–31.11)	29.92	29.45
2019	20.25	30.52 (30.02–31.03)	29.86	29.38
2020	22.75	33.62 (33.10–34.14)	32.92	32.43
2021	22.40	33.35 (32.81–33.90)	32.62	32.12
2022	21.51	32.18 (31.68–32.68)	31.48	31.01
2023	20.90	31.12 (30.66–31.58)	30.48	30.01
2024	20.75	30.59 (30.07–31.11)	29.97	29.54

Note. Estimates use the categorical-year logistic model with the original age-in-months spline, sex, grade, and district, standardized to the original pooled primary-sample distribution. Confidence intervals use school-cluster covariance. All three WHO scenarios use the same records and model covariates; only classification age changes. Full annual intervals for all columns are provided in reviewer3_who_outputs/results/adjusted_annual.csv. The national estimates reproduce the primary analysis. CI, confidence interval.

Supplementary Figure S1. Conservative measurement-resolution sensitivity for the change in adjusted high-BMI prevalence and the conditional P95−P50 difference in 2014–2024 change.

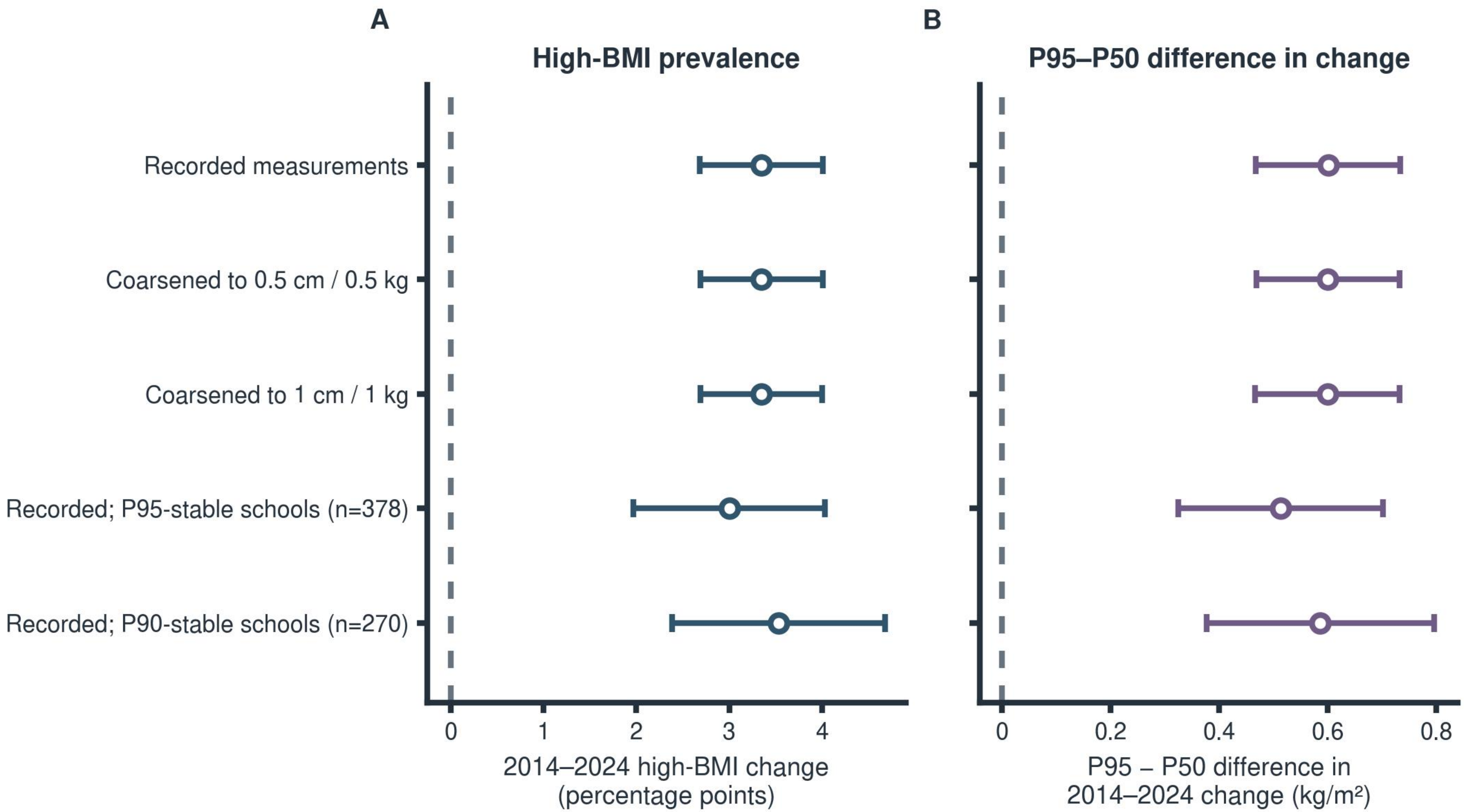

Points show 2014–2024 estimates and horizontal bars show 95% confidence intervals. Panel A presents standardized high-BMI prevalence changes from categorical-year logistic models adjusted for the centered and whitened age-in-months spline, sex, grade and district, with school-cluster inference. Panel B presents the conditional P95−P50 difference in 2014–2024 change using the same covariates and joint school-cluster cross-quantile inference. Recorded measurements are compared with rounding of height/weight to 0.5 cm/0.5 kg and 1 cm/1 kg and empirically resolution-stable school subsets; coarsening is a robustness analysis rather than a correction of recorded measurements.

Supplementary Figure S2. Fixed-panel representation and observed outcomes in all schools and the fixed 704-school panel.

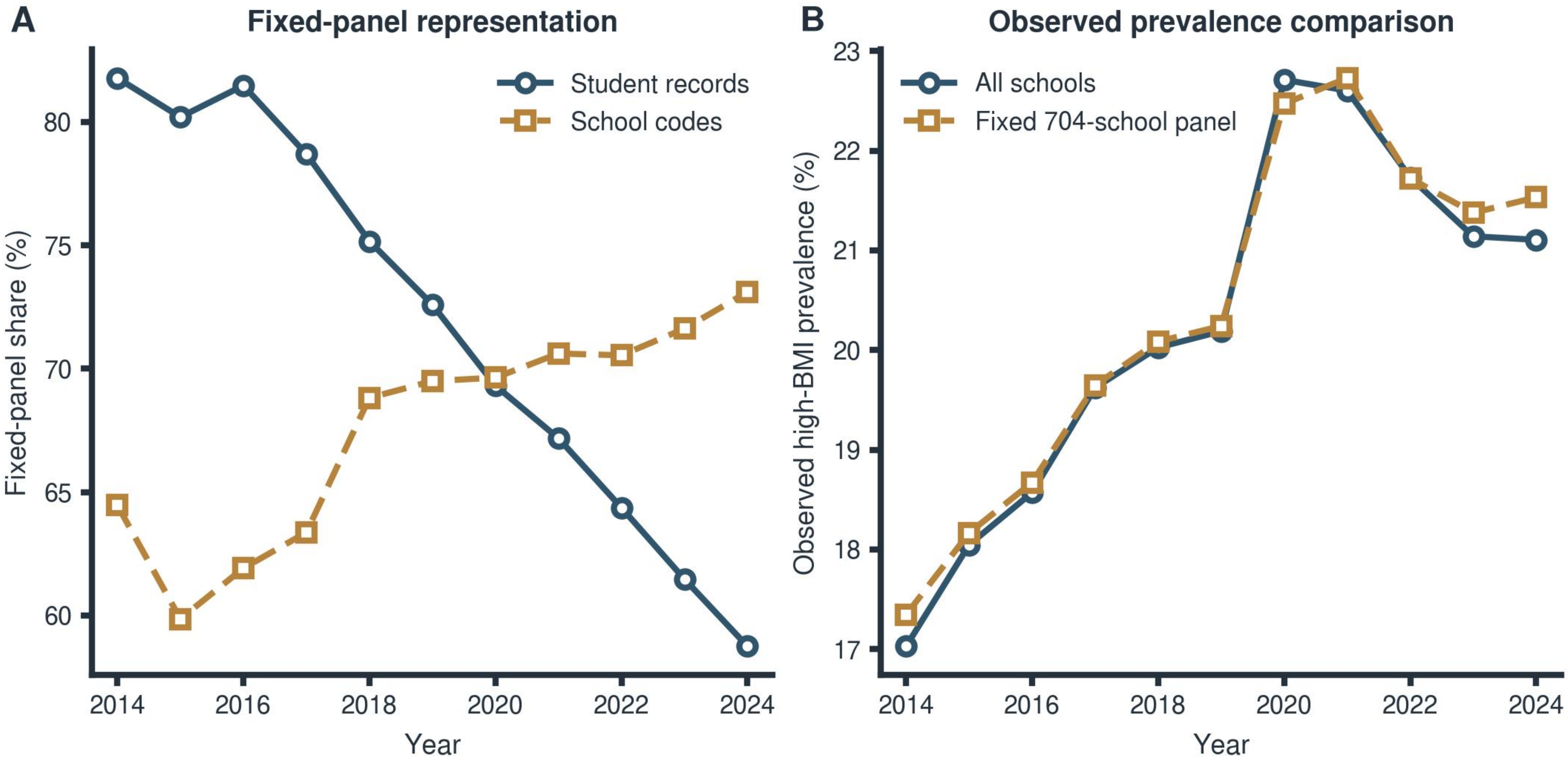

Panel A shows the annual shares of age-valid retained student records and school codes represented by the fixed panel. These shares describe representation within the retained analytic data, not population participation or reporting coverage. Panel B compares observed high-BMI prevalence in all schools and the fixed panel. Primary fixed-panel models use 5,060,353 age-restricted records from 704 schools. These descriptive comparisons assess sensitivity to school entry and exit; they do not establish population representativeness. No confidence intervals are displayed.

Supplementary Figure S3. District patterns of sex-by-grade-standardized high-BMI prevalence, 2014–2024.

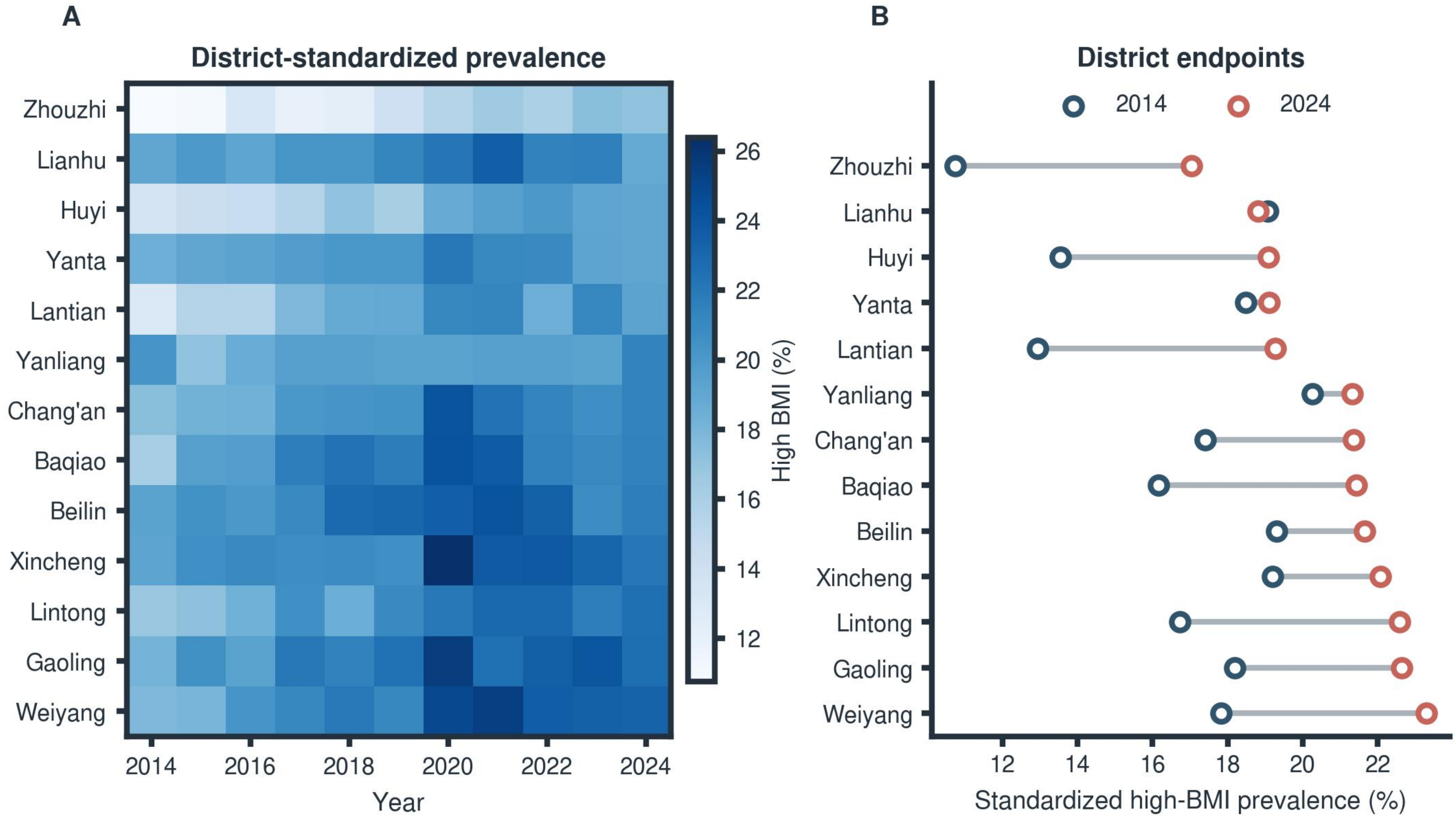

Panel A is a heatmap for 13 districts and Panel B compares 2014 and 2024 endpoints in the same district order. Rates are directly standardized to the pooled primary-sample sex-by-grade distribution and are not adjusted for continuous age in months. This is a secondary descriptive analysis. Pointwise confidence intervals are provided in Table SM5 and the source-data workbook; they are not displayed in this figure.

Supplementary Figure S4. School-level adjusted high-BMI burden-pattern classification and one-factor stability analyses.

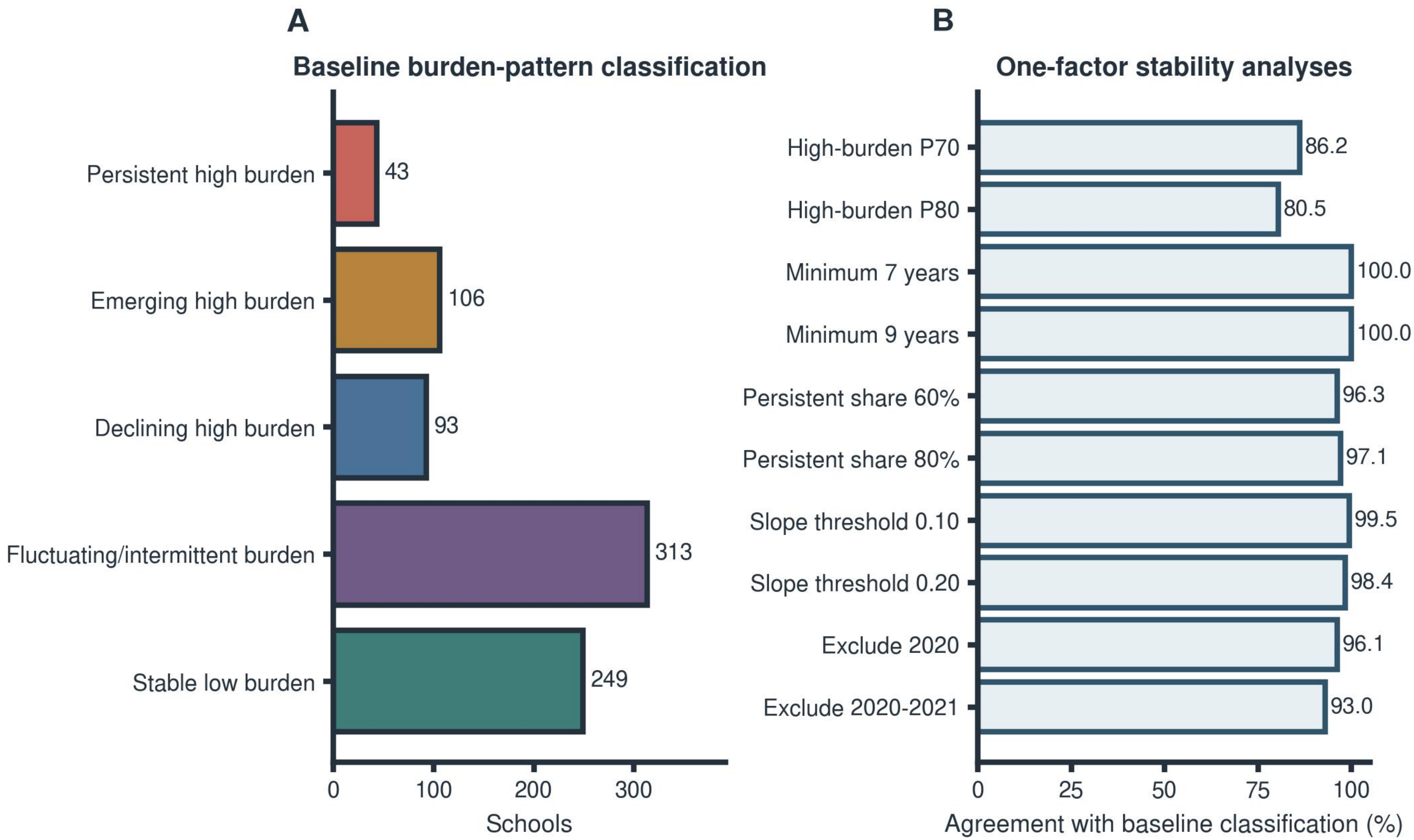

Panel A shows baseline counts among 804 adjusted high-BMI burden-pattern-eligible schools. Panel B shows agreement with baseline classification under alternative thresholds, minimum observed years and exclusions. Categories describe school-level annual relative burden patterns for monitoring and hypothesis generation; they are neither predictions of future risk, individual-child trajectories, nor fixed school diagnoses. No school identifiers are shown.

Supplementary Figure S5. Exploratory 2024 school physical-education-resource associations.

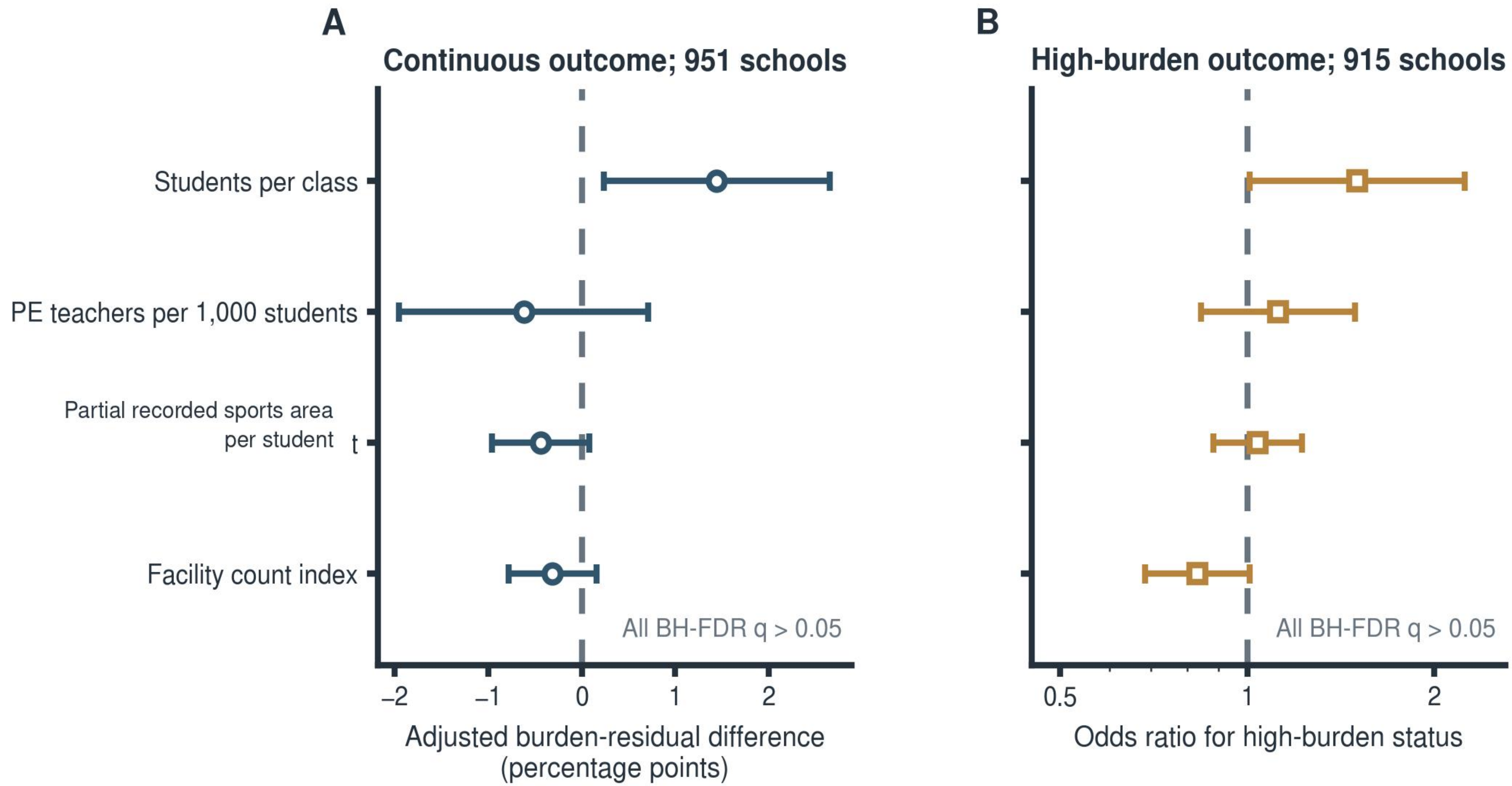

Panel A shows adjusted high-BMI burden-residual differences from student-record-weighted least-squares models in 951 matched schools. Panel B shows odds ratios for high-burden status in 915 schools with at least 50 records (230 high-burden schools). Models mutually include four standardized resource exposures, log school size and district indicators; bars show 95% confidence intervals. The eight tested resource-exposure associations (four per panel) were adjusted together by the Benjamini–Hochberg procedure, and none achieved BH-FDR q<0.05. Exposures were measured only in 2024; administrative counts do not measure resource use, quality, accessibility, PE lesson content, or individual physical activity. Accordingly, the findings neither identify a causal resource effect nor establish no relationship. None of these eight associations met the 0.05 threshold after Holm correction either (adjusted P=0.155–1.000).

Supplementary statistical methods

BMI Distribution and Sex- and Grade-Specific Trends in Primary School Health Surveillance Records in Xi'an, China, 2014–2024: A Repeated Cross-Sectional Study

SM1 Age spline and measurement precision sensitivity

The age-adjustment function used four restricted-cubic-spline knots at the 5th, 35th, 65th, and 95th percentiles of the primary-sample standardized-reference age-in-months midpoint distribution: 6.2917, 7.9583, 9.7083, and 11.6250 years (75.5, 95.5, 116.5, and 139.5 months). With four knots, the spline contributed 3 degrees of freedom (one linear and two nonlinear terms). Four knots were used as a parsimonious compromise: they allowed moderate curvature across the narrow 6.0–12.99-year age range while limiting instability from additional flexibility. Standardized-reference age in years was first scaled as $(\text{age}-9.5)/2.0$. The three raw basis columns were centered using their means on the fixed 72–155 completed-month grid, represented by 72.5–155.5-month midpoints, and whitened by the inverse square root of the grid-basis covariance. Eigenvalues were floored at $\max(10^{-10}\lambda_{\max}, 10^{-12})$. This nonsingular linear transformation preserved the fitted spline function space and was used solely for numerical conditioning. The analysis specification used the four stated percentile knots and included no outcome-driven knot search or comparative tuning step. The analysis was not preregistered.

The terminal-digit stability screens were empirical sensitivity analyses, not external quality-control standards or criteria for exclusion from the primary sample. For each school-year with at least 50 records, we calculated the percentages of height and weight values with a tenths digit of zero (that is, integer-valued records). Across all eligible consecutive-year transitions within schools, we then calculated separate empirical P90 and P95 limits for the absolute percentage-point change in the height and weight percentages. We restricted the analysis to the 704 schools observed in all 11 years and retained a school only if it had at least 50 records in every year and its maximum adjacent-year change in both percentages did not exceed the selected limits. The P90 limits were 27.157 percentage points for height and 46.274 for weight (270 schools; 2,027,243 records); the P95 limits were 43.714 and 70.416 percentage points, respectively (378 schools; 3,054,685 records). P90 and P95 were selected as familiar upper-percentile reference points that create nested sensitivity samples: the lower P90 limits impose a more stringent maximum-change requirement, whereas P95 provides a less stringent screen. Reporting both brackets the result under two degrees of empirical screening while retaining samples large enough for fixed-school analysis. No preregistration record or contemporaneous analysis plan was available to establish that the terminal-digit screening analysis or the P90/P95 choices had been specified before inspection of the data. These thresholds are therefore presented as empirical sensitivity criteria rather than as prespecified or externally validated quality-control standards, and failure to meet a threshold was not interpreted as proof of measurement error.

SM2 High BMI standardization and school clustered inference

The primary high-BMI analysis used the primary analysis sample. We fitted a logistic regression model with year as a categorical variable. The model included a consistently defined restricted cubic spline of the age-in-months interval midpoint, sex, grade, and district. We used a robust covariance estimator clustered by school code with a finite-cluster correction; general methodological support for sandwich covariance estimation and cluster-robust inference is provided elsewhere (1, 2). Annual prevalence was marginally standardized to the pooled 2014–2024 distribution of the age spline × sex × grade × district. The fixed 2014 distribution and a distribution that weighted each year equally were used as alternative standard populations in sensitivity analyses. Estimates for the fixed-school panel were standardized to the panel's own pooled 2014–2024 distribution of the

age spline, sex, grade, and district. We used a 9-df school-clustered Wald test to assess whether the categorical year effects departed from a single linear log-odds trend from 2014 to 2024. School code, rather than school-year, defined the cluster, so all observations from the same school across 2014–2024 contributed to one cluster. The finite-cluster multiplier was $[G/(G-1)]\times[(N-1)/(N-k)]$, where G is the number of schools, N the number of records, and k the number of fitted parameters. For the primary model, G=1,394, N=7,187,779, k=32, and the multiplier was 1.000722; confidence intervals and tests used a t distribution with G−1=1,393 degrees of freedom.

SM3 Conditional quantile estimation and internal validation

Point estimates were obtained by grouped iteratively reweighted least squares over year × district × sex × grade × age-month covariate patterns. Coefficients were initialized at 1, residuals smaller than 10^{-6} in absolute value were set to $\pm10^{-6}$, and fits used a maximum of 1,000 iterations, with convergence defined as a maximum absolute coefficient change below 10^{-5}. All four fits converged within 121–174 iterations.

For each quantile q, the Hall–Sheather probability bandwidth was calculated as Equation (1).

$$h_0=n^{-1/3}[\Phi^{-1}(0.975)]^{2/3}\left\{\frac{1.5\phi(z_q)^2}{2z_q^2+1}\right\}^{1/3}, \quad z_q=\Phi^{-1}(q) \tag{1}$$

Here, n=7,187,779, and Φ and ϕ denote the standard-normal distribution and density functions, respectively. The probability bandwidth in Equation (1) was converted to the BMI scale as Equation (2).

$$h_q=\min\left[SD(BMI),\frac{IQR(r_q)}{1.34}\right]\{\Phi^{-1}(q+h_0)-\Phi^{-1}(q-h_0)\} \tag{2}$$

where r_q denotes the vector of residuals from the model for quantile q, and IQR denotes the interquartile range. The residual density at zero was estimated as Equation (3).

$$\hat{f}_q(0)=\frac{1}{nh_q}\sum_{i=1}^{n}K\left(\frac{r_{iq}}{h_q}\right) \tag{3}$$

where r_{iq} is the residual for record i from the model for quantile q. The Epanechnikov kernel in Equation (3) was defined in Equation (4).

$$K(u)=0.75(1-u^2)I(|u|\leq1) \tag{4}$$

where $I(\cdot)$ denotes the indicator function. The resulting h_q values were 0.065020, 0.057937, 0.057535, and 0.061687 at P50, P75, P90, and P95, respectively. The matrix used for the sandwich bread was $A_q=\hat{f}_q(0)X'X$, where $X'X$ denotes the record-level design-matrix cross-product, evaluated in the grouped implementation using covariate-pattern frequency weights, and the prime denotes matrix transposition. School-level scores were stacked across the four quantiles, and their finite-cluster-corrected cross-products formed the joint covariance. Bootstrap inference used 19,999 independent school-level Rademacher-weight replicates (−1 or +1), batches of 500, seed 20260711, and the finite-replicate +1 correction for two-sided P values.

The pooled residual-density bread uses one density estimate at each quantile for all covariate patterns. School clustering accounts for dependence within schools but does not remove this approximation. The observation-level kernel-weighted sensitivity estimates the bread at each quantile as the sum of $K(r_i/h)x_ix_i'/h$ over records,

retaining the association between residual density and covariates. School-level scores are transformed by each inverse bread and stacked across all four quantiles. Their cross-products, with the same finite-cluster correction, give the joint covariance for the four 2014–2024 coefficients and the three upper-quantile versus P50 contrasts. These sensitivity intervals use the t distribution with 1,393 degrees of freedom.

Two internal samples were used for numerical and inferential validation. First, 51,480 records were sampled without replacement as 30 records from each of 1,716 year × district × sex × grade strata (1,308 schools; seed 20260711). Custom fits used a 2,000-iteration limit and a coefficient convergence tolerance of 10^{-8}. We compared custom P50, P75, P90, and P95 fits with SciPy HiGHS linear-programming solutions using the same observations, design matrix, and check-loss objective. All four custom fits met the convergence criterion, and HiGHS returned optimal solutions. Table SM1 reports the 2024 coefficients and relative objective differences; comparisons for all model coefficients are provided in the code archive.

Table SM1. Internal comparison of grouped IRLS and HiGHS quantile estimates.

Quantile	IRLS 2024 coefficient	HiGHS 2024 coefficient	Absolute difference	Relative objective difference
P50	0.137345	0.138738	0.001392	3.34×10^{-11}
P75	0.389766	0.387571	0.002195	4.02×10^{-10}
P90	0.992836	0.992835	0.000001	9.10×10^{-11}
P95	0.808838	0.808841	0.000003	1.26×10^{-10}

Note. All coefficients and absolute differences are in kg/m²; the 2024 coefficient compares 2024 with 2014. Differences were calculated before rounding. Relative objective difference is the absolute difference between check-loss values divided by the HiGHS check loss. Custom fits converged in 1691, 457, 364, 849 iterations at P50, P75, P90, and P95, respectively. The largest absolute difference across all model coefficients was 0.002963 kg/m². This internal comparison assesses point estimation and does not replace full-sample estimates or validate their covariance estimates.

Second, a simple random sample without replacement of 200,000 primary-sample records (1,361 schools; seed 20260721) underwent 199 school-cluster pairs-bootstrap replicates with complete P50 and P95 refitting. All 199 replicates converged at both quantiles. The standard error of the P95−P50 contrast was 0.120370 kg/m² from the pairs refits and 0.124610 kg/m² from the linearized calculation, giving a ratio of 0.966. These validation-sample values are distinct from the full-sample estimates.

The expanded density-bread sensitivity analysis used the full primary sample of 7,187,779 records and 1,394 school clusters. Models were refitted using the recovered point-estimation routine and its original convergence settings. The largest absolute difference between refitted and archived 2024 coefficients was 0.000033 kg/m²; all four retained the same values at three decimal places. Within this rerun, pooled-density and observation-level kernel-weighted covariances used identical fitted coefficients and school score vectors. Kernel bandwidths were 0.75, 1.00, and 1.25 times the quantile-specific Hall–Sheather bandwidth. All four quantile changes and all three upper-quantile versus P50 contrasts had confidence intervals above zero at every multiplier (all $P<0.001$). Table SM2 reports the default-bandwidth comparison. The code archive provides all 21 kernel-weighted estimates, the joint covariance matrices, and numerical diagnostics. These results assess sensitivity to the bread construction; they do not establish that residual density is constant or verify confidence-interval coverage.

Table SM2. Full-sample sensitivity to the residual-density bread at the default bandwidth.

Change	Estimate	Pooled SE	Kernel SE	Kernel 95% CI	SE change (%)
P50	0.133794	0.027708	0.027829	0.079–0.188	+0.4
P75	0.345129	0.040930	0.041009	0.265–0.426	+0.2
P90	0.620094	0.062391	0.060536	0.501–0.739	-3.0
P95	0.734832	0.082362	0.079913	0.578–0.892	-3.0
P75−P50	0.211335	0.020665	0.020478	0.171–0.252	-0.9
P90−P50	0.486300	0.045297	0.043175	0.402–0.571	-4.7
P95−P50	0.601038	0.067784	0.064985	0.474–0.729	-4.1

Note. Estimates, standard errors (SE), and confidence limits are in kg/m². All changes compare 2024 with 2014. Both SE columns use the same refitted coefficients and school scores. Kernel denotes observation-level Epanechnikov weighting at the default Hall–Sheather bandwidth. SE change is 100 × (kernel SE/pooled SE − 1). Confidence intervals use school-cluster covariance and a t distribution with 1,393 degrees of freedom; all seven kernel-weighted P values are <0.001. Historical main-analysis estimates remain in the main tables.

SM4 School burden classification

Annual school-level adjusted high-BMI burden was defined as a contemporaneous observed-minus-expected residual in percentage points. The original analysis used 7,200,071 records with calculable reference ages and fitted a grouped binomial logistic model to high-BMI events and records aggregated by year × district × sex × grade, with categorical main effects for year, district, sex, and grade. This original specification was a separate exploratory standardization model; individual predicted probabilities from the primary standardized-reference-age spline regression were not aggregated to construct the school-level residual. For school j in year t, expected events were the sum over sex–grade cells of $N_{jtsg} \times \hat{p}_{tdsg}$; expected prevalence was the expected-event sum divided by the school-year N, and the adjusted burden residual was 100 × (observed events/N − expected events/N). This residual describes a contemporaneous difference in high-BMI classification prevalence; it is neither a prediction of future risk nor a measure of adiposity or individual health burden, and it differs from the primary standardized-reference-age-spline marginal estimand. Within each year, the P75 threshold was calculated among school-years with at least 50 records; only school-years meeting that record threshold could be designated high burden. Schools were classifiable if observed for at least 8 years, including at least 2 observations in 2014–2016 and at least 2 in 2022–2024. Persistent high burden denoted high-burden status in at least 70% of observed years. Emerging high burden required at most one early high-burden year, at least two late high-burden years, and a positive N-weighted residual slope. Declining high burden used the converse early/late rule and a negative slope. Stable low burden required at most one high-burden year overall and none in 2022–2024; remaining eligible schools were classified as fluctuating/intermittent burden. School-years with fewer than 50 records could not be designated high burden but remained part of the observation history. Schools failing the observation-history requirements were unclassifiable. Classification rules and sensitivity analyses are reported in Supplementary Table S5. Archived file and variable names retain legacy ‘risk’ labels solely for version integrity.

The original standardization used the sex–grade categories of the national high-BMI classification and did not adjust for age differences within grades. We evaluated this limitation using three specifications: A, the original sample and model; B, the same model restricted to the 7,187,779 records aged 6.0–12.99 years; and C, the same

records as B with the primary age-in-months spline added. Model C retained categorical year, district, sex, and grade and added the centered and whitened four-knot spline with 3 degrees of freedom described in SM1. Expected school-year events were summed over sex × grade × age-month cells. The A–B comparison assessed age-range restriction, whereas B–C assessed monthly-age adjustment on identical records. Annual P75 thresholds were recomputed among school-years with at least 50 records in each specification; the observation-history, persistence, early/late, and slope rules were unchanged. We compared category counts, individual school assignments, unweighted Cohen's κ, and absolute changes in school-year residuals.

All three specifications classified the same 804 schools. Age restriction alone changed 12 classifications (A–B agreement, 98.51%; κ=0.979). Adding monthly-age adjustment on the restricted sample changed 24 classifications (B–C agreement, 97.01%; κ=0.959). The combined A–C comparison changed 30 classifications (agreement, 96.27%; κ=0.948). Tables SM3 and SM4 report category totals and the B–C transition matrix. Across 10,607 school-years with at least 50 records in both B and C, the median, 95th percentile, and maximum absolute residual changes were 0.093, 0.442, and 11.386 percentage points, respectively; 55 changes exceeded 1 percentage point and two exceeded 5 percentage points. Agreement in annual high-burden status was 99.09%. These descriptive comparisons show that most classifications were stable, with changes in a minority of schools. The 2024 PE-resource association models were not refitted in this sensitivity analysis.

Table SM3. School burden classifications under age restriction and monthly-age adjustment.

Classification	A<br>Original	B<br>Age restricted	C<br>Age adjusted
Persistent high burden	43	44	43
Emerging high burden	106	107	108
Declining high burden	93	91	95
Fluctuating/intermittent burden	313	314	314
Stable low burden	249	248	244
Total	804	804	804

Note. Entries are school counts. A uses 7,200,071 records with calculable reference ages and no monthly-age covariate. B uses 7,187,779 records aged 6.0–12.99 years with the same covariates as A. C uses the same records as B and adds the primary monthly-age spline. All models retain categorical year, district, sex, and grade. Annual P75 residual thresholds are recalculated separately; all other classification rules are unchanged. The same 804 schools are classifiable in all three models.

Table SM4. Classification transitions after monthly-age adjustment on the same age-restricted records.

Without age adjustment (B)	P	E	D	F	S	Total
Persistent high burden	42	1	0	1	0	44
Emerging high burden	0	106	0	1	0	107
Declining high burden	0	0	87	4	0	91
Fluctuating/intermittent burden	1	1	5	304	3	314
Stable low burden	0	0	3	4	241	248
Total	43	108	95	314	244	804

Note. Rows show Model B and columns show Model C. P, persistent high burden; E, emerging high burden; D, declining high burden; F, fluctuating/intermittent burden; S, stable low burden. The diagonal contains 780 schools retaining their category; 24 changed category (agreement, 97.01%; unweighted κ=0.959). Both models use the same 7,187,779 records and identical classification rules, with annual thresholds recomputed for each model.

SM5 District standardization and confidence intervals

District prevalence was the weighted sum of the 12 district-year sex–grade proportions in the 7,187,779 primary-analysis records. We retained the pooled 2014–2024 sex–grade proportions as a fixed reference. These directly standardized estimates did not include the age spline used in the primary regression.

We obtained pointwise 95% percentile confidence intervals using 9,999 nonparametric school-cluster bootstrap replicates (seed 20260922). Within each district, we sampled school codes with replacement from all codes observed during 2014–2024, drawing the same number of codes as in that district's observed pool. Each selected school contributed its full available multi-year record history, with one shared bootstrap multiplicity across years (2). We recomputed the 12 stratum proportions and their weighted sum in each replicate, and calculated the 2024–2014 difference from the two estimates in that same replicate. Interval limits were the 2.5th and 97.5th percentiles, using linear interpolation.

The resampling pools contained 26–229 school codes per district. All 9,999 replicates in each district had nonempty stratum denominators. Table SM5 reports endpoint prevalence and change intervals; aggregate code outputs include intervals for all 143 district-year estimates. These intervals are conditional on the fixed reference weights and the observed school records. They do not incorporate uncertainty from nonparticipation, changing reporting coverage, or the estimated reference weights. They are pointwise intervals and do not establish simultaneous coverage, district rankings, or a change in between-district dispersion.

Scope of the retained district analyses

A separate contextual panel had been assembled from Xi'an Statistical Yearbook series, but the original yearbook pages, row-level extraction record, and merge log were unavailable for final source verification. We therefore excluded the contextual-indicator, socioeconomic-ranking, and district ecological-association results. The retained district analysis uses surveillance-derived prevalence and observed record counts and supports no conclusion about socioeconomic concentration or changes in socioeconomic inequality.

Candidate contextual measures required a consistent definition and comparable annual values for all 13 districts during 2014–2024. The stored district panel and its source workbook contained no measure of broadband coverage, subscriptions, access speed, or individual internet use. The available public administrative series reported fixed-broadband subscribers for Xi'an as a whole (3). A citywide series has no within-year district variation and would be absorbed by categorical year indicators. It therefore cannot test whether districts with larger access gains experienced different changes in high-BMI burden. No broadband model was fitted.

Slope and relative indices of inequality and concentration indices are established approaches to ordered socioeconomic comparisons (4–7). These analyses were excluded because comparable district-year socioeconomic data could not be verified. The retained results describe geographic differences in standardized high-BMI prevalence rather than socioeconomic gradients.

Table SM5. District-standardized high-BMI prevalence and endpoint changes with 95% confidence intervals.

District	School codes	2014 prevalence, % (95% CI)	2024 prevalence, % (95% CI)	2014–2024 change, pp (95% CI)
Lintong	153	16.72 (13.70 to 20.61)	22.57 (21.41 to 24.23)	5.85 (1.68 to 9.37)
Zhouzhi	145	10.75 (9.31 to 12.12)	17.05 (15.60 to 18.59)	6.29 (4.47 to 8.43)
Xincheng	38	19.19 (17.94 to 20.34)	22.06 (20.66 to 23.51)	2.88 (0.84 to 4.90)
Weiyang	111	17.83 (16.97 to 18.77)	23.30 (21.94 to 24.80)	5.47 (3.82 to 7.11)
Baqiao	94	16.16 (14.93 to 17.28)	21.43 (20.16 to 22.71)	5.27 (3.77 to 6.85)
Beilin	38	19.30 (17.56 to 21.26)	21.65 (19.76 to 23.87)	2.35 (−0.48 to 4.96)
Lianhu	46	19.06 (17.56 to 20.50)	18.82 (17.86 to 19.80)	−0.25 (−1.96 to 1.64)
Lantian	208	12.95 (11.91 to 14.07)	19.28 (17.60 to 20.75)	6.33 (4.22 to 8.22)
Huyi	124	13.54 (12.06 to 14.95)	19.08 (17.54 to 20.59)	5.54 (3.81 to 7.29)
Chang’an	229	17.40 (16.43 to 18.29)	21.36 (20.37 to 22.42)	3.95 (2.62 to 5.44)
Yanliang	26	20.25 (16.61 to 25.02)	21.31 (19.66 to 22.79)	1.06 (−3.62 to 5.26)
Yanta	107	18.49 (17.32 to 19.69)	19.11 (18.28 to 20.07)	0.62 (−0.81 to 2.18)
Gaoling	75	18.18 (15.59 to 20.79)	22.63 (20.59 to 24.77)	4.44 (1.63 to 7.33)

Note. School codes are the district-specific resampling pools observed at any time during 2014–2024; annual counts may be smaller. Prevalence uses the fixed pooled primary-sample sex–grade distribution. Changes are calculated from unrounded estimates. Intervals are pointwise percentiles of 9,999 school-cluster bootstrap replicates and retain within-school dependence across years. They are not multiplicity-adjusted. pp, percentage points; CI, confidence interval.

SM6 Software

The primary record-level analyses were conducted in Python 3.12.13 using NumPy 2.3.5, pandas 2.2.3, SciPy 1.17.0, Matplotlib 3.10.8, and Pillow 12.2.0. Data import and aggregate-workbook output additionally used openpyxl 3.1.5. The categorical-year logistic, grouped conditional-quantile, cluster-sandwich, joint cross-quantile covariance, and multiplier-bootstrap routines were implemented in custom Python code rather than through a third-party quantile-regression package and were validated as described above. The expanded four-quantile validation, full-sample density-bread sensitivity analysis, school-classification age sensitivity analysis, district bootstrap analysis, and four-school inclusion sensitivity analysis used Python 3.12.14 with the same NumPy, pandas, and SciPy versions.

The supplementary code archive retains the recovered analysis scripts and aggregate validation files. The original HiGHS validation routine is validate_qr in primary_age_models.py; quantile_regression_validation.py contains the local-bread and school-cluster pairs-refit routines. The expanded four-quantile comparison was executed with stage3_additions/run_all_quantiles.py, which imports the recovered grouped IRLS and HiGHS routines. Its aggregate outputs and run metadata are supplied in stage3_validation_outputs. The full-sample density-bread sensitivity used stage4_additions/run_covariance_sensitivity.py; verification of its exported results used stage4_additions/verify_exported_results.py. Outputs, diagnostics, and execution metadata are supplied in stage4_covariance_outputs. The school-classification age sensitivity used stage5_additions/school_age_sensitivity.py, which imports the recovered primary-model functions and extracts the unchanged classification rule from round30_secondary_exclude4.py. Aggregate outputs and run metadata are provided in stage5_school_age_outputs; record-level data and school identifiers are excluded. The district confidence intervals were calculated with stage6_additions/district_cluster_intervals.py; aggregate estimates, diagnostics, and run metadata are provided in stage6_district_outputs. Historical outputs are retained separately. Input schemas and execution requirements are described in the archive README. The four-school inclusion analysis uses stage8_additions/run_four_school_sensitivity.py, with verification by stage8_additions/verify_and_summarize.py. Aggregate outputs are in stage8_inclusion_outputs. Supplementary Methods SM7 describes this additional analysis.

The current archive entry point is README_REVIEWER3_COMPLETE.md, and SHA256_REVIEWER3_COMPLETE.json is the complete checksum manifest for this documentation revision. Earlier stage-specific READMEs and logs are historical records; their pending-status statements describe those earlier stages. A runnable artificial-data example is provided in stage2_additions/check_recovered_outputs.py. It generates 1,200 records and 40 artificial school clusters and compares grouped IRLS with linear programming at all four quantiles. This is a software demonstration, separate from the study-sample validation in Table SM1. The README provides the command and identifies the current study outputs.

The lower-quantile extension used Python 3.12.14, NumPy 2.3.5, pandas 2.2.3, and SciPy 1.17.0. Scripts are in reviewer3_lower_additions; aggregate estimates, validation results, and diagnostics are in reviewer3_lower_outputs. Execution commands for the lower-quantile models are in README_REVIEWER3.md; README_REVIEWER3_COMPLETE.md is the current archive guide. The WHO extension used the same software versions. Its scripts, reference-source manifest, algorithm checks, aggregate outputs, and model diagnostics are supplied in reviewer3_who_additions and reviewer3_who_outputs.

SM7 Sensitivity to inclusion of the four school codes

The four school codes changed their recorded district once between 2019 and 2020. The historical analysis excluded all records for these codes to avoid uncertain cross-year district attribution. The available study records did not establish whether the changes represented administrative reassignment or reporting-field changes. They did not establish that the anthropometric measurements were invalid.

This additional, non-preregistered sensitivity analysis included their 71,532 QC-valid records. Four lacked a calculable birth date and 49 were outside the primary age range. The remaining 71,479 records increased the primary-analysis sample from 7,187,779 records and 1,394 school clusters to 7,259,258 records and 1,398 clusters. We retained the district recorded for each survey year and grouped all years of each school into one cluster. No retrospective district reassignment was applied.

High-BMI and subgroup models were refitted using the original routines, eligibility criteria, BMI cut-points, spline knots, and interaction specifications. The original pooled reference distribution and within-grade reference weights were held fixed to isolate the effect of adding records to the fitted models. Using the expanded sample's own pooled standard was a supplementary comparison. Quantile models used the same covariates and original convergence settings. School-cluster covariance and joint cross-quantile inference followed SM2–SM3, with 1,397 degrees of freedom for the expanded sample. The original 19,999 quantile and 9,999 subgroup multiplier checks were retained. Quantile intervals were also recalculated with observation-level kernel-weighted bread at the default bandwidth.

Table SM6 compares the principal estimates. The excluded-school quantile column uses the full-sample rerun reported in SM3, with identical input-array and core-code checksums verified; its estimates differ from the archived main-model coefficients by less than 0.000034 kg/m². The excluded-school logistic and subgroup models were refitted for this comparison. All new models converged. All four conditional quantile changes and all three upper-quantile versus median contrasts in the expanded sample had confidence intervals above zero with both pooled-density and default-bandwidth kernel-weighted covariance. Full annual and subgroup estimates, confidence intervals, multiplier checks, convergence diagnostics, and sample-count checks are supplied in the code archive.

The comparison assesses sensitivity of the primary and subgroup findings to this exclusion under the recorded district assignments. It does not verify why those fields changed or establish that all-year exclusion was necessary. District descriptive summaries, school burden classifications, and PE-resource associations were not refitted in this analysis.

Table SM6. Primary and subgroup changes with and without the four school codes.

Estimand	Unit	Four schools excluded Estimate (95% CI)	Four schools included Estimate (95% CI)	Change in estimate
High-BMI prevalence change	pp	3.346 (2.685 to 4.007)	3.316 (2.662 to 3.971)	−0.030
P50 change	kg/m²	0.134 (0.079 to 0.188)	0.133 (0.079 to 0.187)	−0.001
P75 change	kg/m²	0.345 (0.265 to 0.425)	0.342 (0.263 to 0.422)	−0.003
P90 change	kg/m²	0.620 (0.498 to 0.742)	0.617 (0.496 to 0.738)	−0.003
P95 change	kg/m²	0.735 (0.573 to 0.896)	0.729 (0.569 to 0.889)	−0.006
P75−P50 difference in change	kg/m²	0.211 (0.171 to 0.252)	0.210 (0.170 to 0.250)	−0.001
P90−P50 difference in change	kg/m²	0.486 (0.397 to 0.575)	0.484 (0.396 to 0.572)	−0.002
P95−P50 difference in change	kg/m²	0.601 (0.468 to 0.734)	0.596 (0.465 to 0.728)	−0.005
Boys−girls difference in change	pp	1.652 (1.169 to 2.136)	1.624 (1.136 to 2.111)	−0.029
Grade 6−Grade 5 difference in change	pp	0.793 (−0.061 to 1.647)	0.786 (−0.061 to 1.632)	−0.008
Boys−girls within Grades 4–6	pp	2.591 (1.953 to 3.229)	2.573 (1.932 to 3.214)	−0.018

Note. All estimates describe 2014–2024 change or a difference between changes. Standardized prevalence and subgroup estimates use the original primary-sample reference distribution. Both columns use school-cluster-robust 95% confidence intervals; the quantile columns use the pooled-density covariance for comparability. Change in estimate equals the included-school estimate minus the excluded-school estimate, calculated before rounding. The two columns contain overlapping samples and are not independent groups; this last column is descriptive. pp, percentage points; CI, confidence interval.

SM8 Exploratory lower-quantile analysis

To extend coverage of the conditional BMI distribution, exploratory models at P5, P10, and P25 were added during peer review. Their scope and checks were recorded before fitting these three quantiles; this was not a preregistered study analysis. The extension retained the primary sample, BMI values, categorical year, sex, grade, district, and centered and whitened monthly-age spline. At each quantile, the 2024 coefficient estimates the covariate-adjusted change in the conditional BMI quantile relative to 2014. Primary P50, P75, P90, and P95 estimates and their formal upper-quantile versus median contrasts were retained.

Full-sample fitting used the unchanged grouped IRLS routine, a 1,000-iteration limit, and a maximum coefficient-change tolerance of 10^{-5}. P5, P10, and P25 converged in 155, 137, 135 iterations, respectively, without detected cycles. Pooled-density school-cluster covariance, the finite-cluster correction, and t intervals with 1,393 degrees of freedom followed SM3. The intervals are pointwise; no additional cross-quantile comparisons were tested in this extension.

Sensitivity analyses replaced the pooled-density bread with observation-level Epanechnikov-kernel-weighted matrices at 0.75, 1.00, and 1.25 times the quantile-specific Hall–Sheather bandwidth. Coefficients and school scores were held fixed within each quantile. All bread matrices were positive definite and full rank; grouped matrix accumulation agreed with direct record-wise algebra in a 10,000-record deterministic subset. At all three bandwidth multipliers, the P5 interval excluded zero and the P10 and P25 intervals included zero. Default-bandwidth intervals are reported in Table S15; all bandwidth results are in the code archive.

An additional linearized check used 19,999 school-level Rademacher multiplier draws with seed 20260711 for each covariance variant. The multiplier-to-analytic standard-error ratios ranged from 1.002 to 1.008, with the same pattern of interval inclusion of zero. These checks assess sensitivity to covariance construction and linearized resampling; they are not full model-refit bootstrap validation or evidence of confidence-interval coverage.

Numerical validation of the lower quantiles

The same fixed internal sample used for Table SM1 was reused: 51,480 records, comprising 30 records from each year × district × sex × grade stratum and 1,308 schools. The sample-index and design/outcome checksums matched the archived validation sample. Custom fits used a 2,000-iteration limit and tolerance of 10^{-8} and were compared with HiGHS linear-programming solutions on identical data and model matrices. All three custom fits converged without detected cycles, and all three HiGHS solves returned successful optimal status. Table SM7 reports objective and endpoint-coefficient discrepancies separately.

Table SM7. Internal numerical comparison of lower-quantile estimates.

Quantile	IRLS coefficient	HiGHS coefficient	Absolute difference	Relative loss difference
P5	0.097114	0.097117	0.000003	3.543e-10
P10	0.017738	0.017737	0.000002	1.634e-10
P25	0.025340	0.026380	0.001040	6.601e-11

Note. Coefficients and absolute differences are in kg/m². These values describe the 51,480-record validation sample and must not replace the full-sample estimates in Table S15. Relative loss difference is the absolute check-loss difference divided by the HiGHS check loss. IRLS converged in 927, 682, and 1,025 iterations for P5, P10, and P25, respectively. The maximum absolute discrepancy across all coefficients was 0.003318 kg/m². This comparison validates numerical agreement of point estimators within the internal sample; it does not establish full-sample exact optimization or validate covariance estimates.

SM9 WHO classification and classification-age sensitivity

This sensitivity analysis was added during peer review to assess how prevalence levels and changes depended on BMI classification. Its scope was recorded before calculating study WHO outcomes. The National Student Physical Health Standard (2014 Revision) remained the primary classification for the school-surveillance analysis. The WHO 2007 sex-specific BMI-for-age reference defined high BMI as Z score >+1, including obesity, and obesity as Z score >+2 (8).

WHO classification used unrounded BMI recalculated in double precision from source height and weight. Record alignment, stored float32 BMI values, and national classifications matched the prepared data. Using float32 instead of double-precision BMI changed none of the WHO high-BMI classifications at September 1.

WHO age in months was calculated as reference age in days divided by 30.4375. L, M, and S were linearly interpolated between adjacent monthly reference values, following the WHO macro. The LMS transformation was used within ±3 SD, with WHO linear tail extension beyond those values (9). Unrounded Z scores were compared with the strict >+1 and >+2 cutoffs; Z scores were not rounded before classification. This differs from the legacy SAS macro's intermediate rounding to two decimals.

The Python implementation was checked against the 933-record example supplied with the WHO SAS macro: all 919 non-missing BMI-for-age Z scores agreed after rounding to the example's two-decimal precision, and the missing-value pattern matched. LMS values in the official Excel and SAS tables were identical over their common monthly age range. Reconstructed centiles agreed with the published three-decimal tables within 0.000501 kg/m². These checks validate the reference calculation; they do not validate the surveillance measurements.

Classification-age scenarios used September 1, October 15 (+44 days), and November 15 (+75 days), while retaining the original age-eligible sample of 7,187,779 records from 1,394 school codes. Eligibility, model age covariates, spline knots, and pooled standardization weights were held fixed to isolate the effect of classification age. The latter two dates represent the stated testing-window endpoints, not observed individual measurement dates. They do not reconstruct the effects of individual timing or changing eligibility.

The classification-only analyses retained the original sample. At September 1, 32,594 records (0.453%) had WHO BMI-for-age Z scores outside −5 to +5: 10,504 below −5 and 22,090 above +5. An additional sensitivity analysis excluded these records, leaving 7,155,185 records and all 1,394 school codes. We fitted both WHO and national outcomes in this same flag-excluded sample, using the original full-sample target weights, to distinguish classification sensitivity from sample-exclusion effects. The October 15 and November 15 scenarios flagged 31,186 and 30,229 records, respectively; their main estimates retained the original sample.

The archived categorical-year logistic routines were used unchanged for annual prevalence and endpoint changes, with the original covariates and school-cluster covariance. The three subgroup interaction models were also fitted under WHO classification at September 1, retaining empirical age distributions within grade and the original sex and grade reference weights. The targeted contrasts were boys−girls overall, boys−girls within Grades 4–6, and Grade 6−Grade 5. The upper-grade sex contrast used the same pooled Grades 4–6 weights; it did not compare Grades 4–6 with Grades 1–3. All nine overall or interaction fits converged in five iterations. Intervals were pointwise t intervals with 1,393 degrees of freedom; subgroup contrasts also retained the 9,999 school-score multiplier check.

Tables S16–S17 and the code archive provide annual estimates, all subgroup results, classification counts, diagnostics, and reproducibility materials. WHO sensitivity was restricted to prevalence and subgroup analyses; district summaries, school burden classifications, and PE-resource analyses retain national classification.

References

1. Liang KY, Zeger SL. Longitudinal data analysis using generalized linear models. Biometrika (1986) 73:13–22. doi: 10.1093/biomet/73.1.13

2. Cameron AC, Miller DL. A practitioner's guide to cluster-robust inference. J Hum Resour (2015) 50:317–72. doi: 10.3368/jhr.50.2.317

3. Shaanxi Communications Administration. Communications-service development statistics by prefecture-level city, December 2023 and December 2024. Available at: https://shxca.miit.gov.cn/xxgk/xyjd/xyfz/art/2024/art_68dac47846834edf95fe3247dba436c1.html; https://shxca.miit.gov.cn/xxgk/xyjd/xyfz/art/2025/art_fa2d78be59da4477a7dd86d6c11716a8.html [in Chinese] [Accessed August 19, 2026].

4. Wagstaff A, Paci P, van Doorslaer E. On the measurement of inequalities in health. Soc Sci Med (1991) 33:545–57. doi: 10.1016/0277-9536(91)90212-U

5. Kunst AE, Mackenbach JP. Measuring Socio-Economic Inequalities in Health. Copenhagen: WHO Regional Office for Europe (1994). Report No.: EUR/ICP/RPD 416 12234.

6. O'Donnell O, van Doorslaer E, Wagstaff A, Lindelow M. Analyzing Health Equity Using Household Survey Data: A Guide to Techniques and Their Implementation. Washington, DC: World Bank (2008). doi: 10.1596/978-0-8213-6933-3

7. Barros AJD, Victora CG. Measuring coverage in MNCH: determining and interpreting inequalities in coverage of maternal, newborn, and child health interventions. PLoS Med (2013) 10:e1001390. doi: 10.1371/journal.pmed.1001390

8. World Health Organization. BMI-for-age (5–19 years): WHO growth reference 2007. Available at: https://www.who.int/tools/growth-reference-data-for-5to19-years/indicators/bmi-for-age [Accessed September 27, 2026].

9. World Health Organization. Growth reference data for 5–19 years: application tools and computation of centiles and Z-scores. Available at: https://www.who.int/tools/growth-reference-data-for-5to19-years/application-tools; https://cdn.who.int/media/docs/default-source/child-growth/growth-reference-5-19-years/computation.pdf [Accessed September 27, 2026].
